# Post-2020 conservation priorities are threatened by global mining

**DOI:** 10.1038/s41467-026-77626-6

**Published:** 2026-09-21

**Authors:** Kaixuan Pan, Niels Raes, Wassim Le Lann, Leon Marshall, Ana Feijoo Quezada, Valerio Barbarossa, Merijn Moens, Sarah Anderson, Rene Kleijn, Jacobus C. Biesmeijer

**Affiliations:** 1 Naturalis Biodiversity Center, Leiden, The Netherlands; 2 Institute of Environmental Sciences, Leiden University, Leiden, The Netherlands; 3 Laboratoire d’Économie d’Orléans, University of Orléans, Orléans, France

**Keywords:** Biodiversity, Conservation biology, Biodiversity, Conservation biology, Environmental impact

## Abstract

The United Nations has committed to halting and reversing biodiversity loss by 2030. Despite this commitment, mining remains a major driver of biodiversity decline, particularly in conservation priority zones (CPZs) and areas with high species extinction risk. Regardless of growing awareness, the full extent of current and future mining threats to biodiversity remains inadequately quantified, limiting effective global conservation strategies. Here, we quantify the threats of 145,000 km² of current mining land to biodiversity. We find that 34% (49,000 km²) of these mining areas overlap with CPZs, intersecting the ranges of more than 2,300 species, including many classified as threatened. These threats span 14 biomes, with disproportionately high relative impacts in fragile ecosystems such as Mangroves and the Mediterranean. Furthermore, an additional 43% (12 million km²), 29% (2 million km²), and 37% (4 million km²) of land potentially exploitable for metals, non-metals, and coal, respectively, overlap with CPZs, highlighting the heightened risks from future mining expansion without consideration for biodiversity. Our findings offer actionable insights for policymakers and industry stakeholders, guiding conservation efforts to mitigate biodiversity loss and align mining practices with global conservation goals, including the UN Sustainable Development Goals and the Kunming-Montreal Global Biodiversity Framework.

## Abstract (web summary)

Mining threatens biodiversity worldwide, yet its full impact remains unclear. Here, authors reveal that extensive current and future mining areas overlap with conservation priority zones and threatened species.

## Introduction

Human activities are profoundly reshaping the planet, driving significant biodiversity loss and habitat degradation^1,2^. To address these challenges, the United Nations Sustainable Development Goals (SDGs) and the Post-2020 Kunming-Montreal Global Biodiversity Framework (GBF) set ambitious targets to restore and protect global biodiversity and to prosper with nature by 2050. A central goal is to prevent further loss of critical and vulnerable ecosystems and to halt human-induced extinctions of known threatened species by 2030^3^. However, the pursuit of economic growth persists in posing serious threats to biodiversity^4^. Managing the escalating demand for resources and mitigating their environmental impacts is essential to achieving the Kunming-Montreal global biodiversity targets.

In recent decades, global mining and quarrying (referred to as mining hereafter) has surged considerably, with raw material extraction nearly doubling since 2000^5^. This growth is driven by increased demand for consumer goods, infrastructure, construction and the energy transition^5^. Projections suggest a continued expansion of mining, primarily driven by the energy transition^5–8^. However, this rapid expansion poses a grave threat to global biodiversity. Mining causes habitat destruction, pollution and ecosystem fragmentation^9^, leading to both direct and indirect biodiversity loss^10–13^. The threats are particularly severe in the biodiversity hotspots of countries like China, Brazil, Congo and Indonesia, where environmental regulations may be less robust^14,15^. As outlined in GBF target 8—Minimise the Impacts of Climate Change on Biodiversity and Build Resilience—it is crucial to recognise that mining, particularly for energy transition, should not come at the expense of biodiversity^3^.

Despite advances in understanding mining’s threats to biodiversity^10–12,16,17^, key knowledge gaps persist. Global mining assessments typically rely on incomplete point-based data^11,12,16^, which fail to capture the full spatial extent of mining impacts and overlook site-specific variations in biodiversity effects^18^. Given the uneven global distribution of biodiversity^19^ and the spatially variable nature of mining-related disturbance^18^, there is a pressing need for high-resolution, spatially explicit analyses that integrate precise mining footprint data with biodiversity metrics. Although some studies have assessed the local impacts of mining, they are often geographically limited and do not offer a global perspective on its threats to biodiversity^9,20–22^. Recent assessments have used IUCN threat listings^17^ or modelled pressures based on deposit locations^11^, but these approaches are typically based on point data^11^ or expert-derived threat data applied at coarse spatial resolutions^17^. To date, no global analysis has combined spatially explicit mining footprint data with conservation priority zones (CPZs) and species-level extinction risk metrics. This gap limits our ability to develop effective conservation strategies that account for real-world spatial overlap between mining and biodiversity values.

In particular, our understanding of how mining overlaps with conservation priority areas and extinction risks across biomes remains limited, further hampering strategic conservation investments in critical habitats. Beyond current mining activities, however, the potential conflicts between future mining expansion and biodiversity conservation remain even less well understood. Addressing this gap is essential for anticipating and mitigating future threats to biodiversity and critical ecosystems, while informing proactive conservation planning.

Future mining is heavily influenced by socio-economic, geopolitical and technological trends, such as the global push for renewable energy and infrastructure development, which drive demand for specific materials like energy resources, metals and non-metals. Since different commodities follow distinct future demand trajectories and spatial extraction patterns, material-specific and spatially disaggregated analyses are essential. Quantifying the biodiversity impacts of different commodities can help identify where the greatest benefits may be achieved through demand-reduction policies and support more sustainable development. Furthermore, understanding where different minerals occur and how their extraction intersects with biodiversity can guide more targeted, risk-informed strategies. For instance, minimising biodiversity loss from coal versus emerging metal mining may require prioritising conservation action in different regions. Such differentiation is key to planning sustainable mining futures that avoid or reduce biodiversity impacts.

In this study, we aim to provide a comprehensive global, spatially explicit analysis of how current and future mining activities intersect with areas of high biodiversity value. Specifically, we address three key questions: (1) To what extent do current mining footprints overlap with international CPZs and areas of elevated species mean extinction risk (MER)? (2) How do current mining-related impacts on CPZ and MER vary across biomes? (3) Where are the most at-risk regions from projected future exploitable mining areas? By integrating high-resolution mining data with biodiversity and conservation datasets, we identify spatial priorities for mitigating biodiversity impacts and informing more sustainable mining practices. Our findings aim to support conservation planning by highlighting areas where trade-offs between mining and biodiversity protection are most pronounced, and where targeted management efforts may be needed to mitigate future ecological impacts.

## Results

### Global distribution of mining and biodiversity threats

**Fig. 1:**
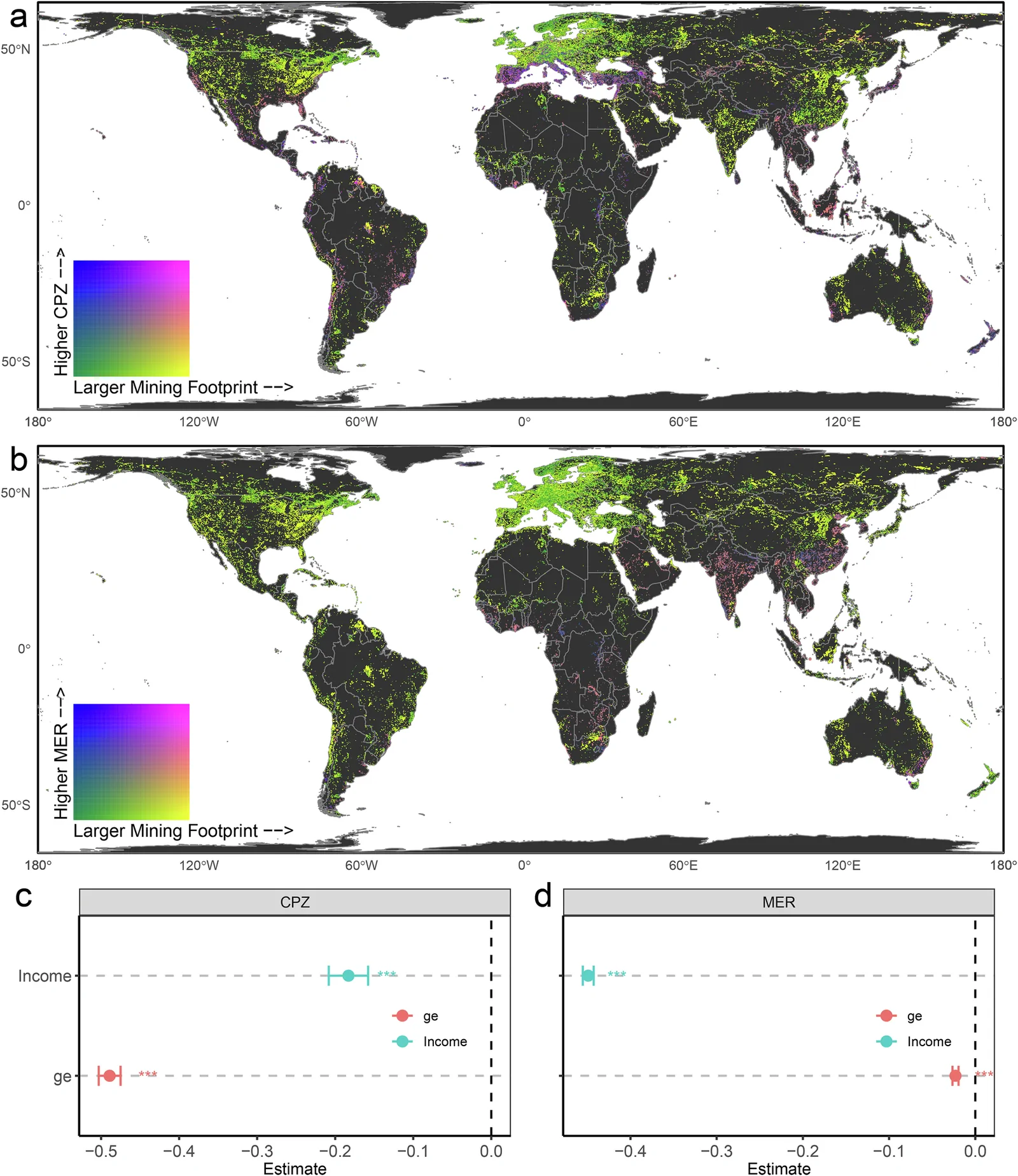
Global distribution of mining threats to conservation priority zones (CPZs) and mean extinction risk (MER) of IUCN Red List species. **a** Relationship between CPZ and mining footprint polygon size. **b** Relationship between MER and mining footprint polygon size. Colours represent: green, low conservation importance and small polygon size; blue, high conservation importance and small polygon size; yellow, low extinction risk and large polygon size; magenta, high extinction risk and large polygon size. Effects of income level and government effectiveness (ge) on mining-related biodiversity impacts (**c** probability of CPZ overlap; **d** MER). Points show model coefficient estimates from binomial and Gamma GLMs, respectively, and horizontal error bars represent 95% confidence intervals (estimate ± 1.96 × standard error). Income level and ge were estimated from separate models to avoid multicollinearity. No adjustment for multiple comparisons was applied because each predictor yields a single model coefficient. Significance: ***p < 0.001, **p < 0.01 and *p < 0.05 (two-sided Wald z-tests; exact *p*-values and full model outputs are provided in Supplementary Tables 5 and 6). *N*_total_ = 267,127 mining polygons for all models. Negative estimates indicate that higher income or ge are associated with lower probability of CPZ overlap and lower MER. For the maps, mining footprints were reclassified by quantiles and scaled without units ('Methods'). CPZ retained its original categorical classification (levels 1–4). MER was classified using natural breaks corresponding to ecologically meaningful intervals (0–1, 1–2, 2–3 and 3–4). This approach highlights absolute high-risk hotspots but may classify fewer areas as extreme and overlook lower-risk areas where few threatened species occur; a quantile-based MER map is provided in Supplementary Fig. 10 and may additionally support percentage-based conservation targets. Original mining footprints ranged from (0 to 2000] km^2^. MER and CPZ are unitless. MER ranges from [0 to 4], where 0 indicates no extinction risk and 4 represents the highest risk. CPZ is a categorical variable, with values ranging from [1 to 4], where 1 indicates no overlap with conservation priority zones and 4 represents overlap with the most critical conservation areas. All spatial data layers were reprojected to the World Behrmann equal-area projection (EPSG:54017).

Globally, mining footprints cover at least 145,000 km² (Supplementary Table 1), with 16% (24,000 km²) dedicated to fossil fuel mining (primarily coal), 38% (55,000 km²) to metal mining (e.g. copper, zinc and nickel), 14% (20,000 km²) to non-metal mining (e.g. limestone, garnet, sand and gravel), and 43% (62,000 km²) classified as unknown mining (encompassing minerals not identified as fuels, metals or non-metals). While most mining records are located in the Northern Hemisphere (Supplementary Fig. 1), there are several notable peaks of mining in certain regions of the Southern Hemisphere. Globally, approximately one-third of all mining areas, spanning ~50,000 km^2^, an area similar to the size of Costa Rica, overlap with international CPZs (excluding CPZ level 1, which indicates no overlap with global CPZs), with 1% (2000 km^2^) occurring within zero-extinction zones (CPZ level 4), which house some of the most critically endangered species (Fig. 1 and Supplementary Table 2). The overlap of mining activities with these zones threatens the no loss goal set by the GBF^3^, highlighting the need for stronger regulatory protection for these ecosystems. In addition, a total of 2311 species are impacted by mining, with 1057 species classified as critically endangered (CR), endangered (EN), or vulnerable (VU) (~9% of all threatened terrestrial vertebrates and plants identified by IUCN; Supplementary Table 3). Notably, mining activities can also trigger off-site and indirect ecological impacts beyond their immediate footprint, including not only deforestation and illegal hunting, but also air, water and soil contamination^9,23–25^. These effects can extend across landscapes and compromise biodiversity well beyond mined areas, particularly due to regulatory failures^9^. Under the scenario where these impacts spread just 5 km beyond the footprint, the area of overlap with various levels of CPZs and MER could increase by 28–55 times and 53–566 times, respectively, assuming total habitat destruction with no remaining ecological functionality (Supplementary Table 4). This underscores that insufficient legal and regulatory frameworks, together with the unplanned expansion of existing mines, can substantially amplify potential threats to biodiversity.

The spatial distribution of mining threats on CPZs and MER varies across regions (Fig. 1). The highest pressure on CPZs, defined as the overlap of mining activities with the highest-level CPZs, occurs in Southeast Asia (e.g. Indonesia, Malaysia and Philippines), Australia, New Zealand, Middle East, Southern Europe, North and West Africa and parts of the Americas (e.g. Brazil, Chile, Colombia, Mexico, and the eastern and western United States) (Fig. 1a). Supplementary Fig. 2 further illustrates how mining threatens CPZs both inside and outside existing protected areas. Most mining footprint occurs outside protected areas (Supplementary Fig. 2a, b), with a small percentage taking place within CPZs (Supplementary Fig. 3a). However, within protected areas, over 60% (~7000 km^2^) of mining footprints threaten CPZs, and 11% (~1000 km^2^) directly intersect zero-extinction zones (CPZ level 4) (Supplementary Fig. 3a). These findings underscore the weaknesses in current conservation management^26,27^. Extraction activities within protected areas can undermine conservation goals and reduce the effectiveness of these protected areas^28,29^.

In terms of species extinction risks, regions facing the highest MER include China, India, Southeast Asia, Australia, the Middle East, Southern and Western Africa and parts of South America (Fig. 1b). Although most mining activities occur outside protected areas, they still lead to greater extinction risks (Supplementary Fig. 3b; d.f. =  1, *χ*^2^ = 815, *p* < 0.001). Therefore, it is essential to acknowledge that evaluating and mitigating the local threats posed by industrial activities to biodiversity is crucial, even outside protected areas, as many unprotected areas serve as critical habitats for numerous threatened species^26^.

When summarising polygon-level mining threats to CPZs and MER at the national level, regions facing the highest pressures with the highest CPZs and MER are predominantly found in lower-income countries, including China, Indonesia, Brazil, Colombia and Mexico (Fig. 1, Supplementary Tables 5 and 6; CPZs: estimate = − 0.18, d.f. = 267,126, *p* < 0.001, 95% CI [−0.21, −0.16]; MER: estimate = − 0.45, d.f. = 267,126, *p* < 0.001, 95% CI [−0.45, −0.44]). This pattern aligns with previous studies showing that extinction risk tends to be higher in lower-income countries^30,31^. These regions are often biodiversity-rich, but face intersecting challenges, including a lack of stringent regulatory oversight, strong dependence on natural resource extraction, competing economic development priorities and higher levels of economic inequality, all of which can exacerbate species extinction risks^14,32–34^. We also found a significant influence of management effectiveness on mining biodiversity risks (Fig. 1c, d, Supplementary Tables 5 and 6; CPZs: estimate = − 0.49, d.f. = 267,126, *p* < 0.001, 95% CI [−0.5, −0.47]; MER: estimate = − 0.02, d.f. = 267,126, *p* < 0.001, 95% CI [−0.03, −0.02]), which appears to conflict with some previous findings reporting no effect of management^30^. Management effectiveness is highly correlated with national income, and more effective governance can substantially reduce illegal mining, which is a major driver of biodiversity loss^14,23^. This pattern likely reflects that both national income and governance constrain the extent to which mining activities overlap high-priority conservation areas and regions of high extinction risk. It may also indicate that many CPZs and habitats for threatened species are not fully integrated into national planning and regulatory frameworks globally^35^, particularly in lower-income or poorly governed countries, potentially leading to under-recognition of biodiversity risks and challenges for achieving global conservation targets such as the Global Biodiversity Framework. Together, these findings suggest that mining pressures on conservation priority areas and extinction risks are disproportionately severe in lower-income countries, highlighting the need for targeted mitigation and strengthened conservation efforts in these regions.

### Biome-specific threats

**Fig. 2:**
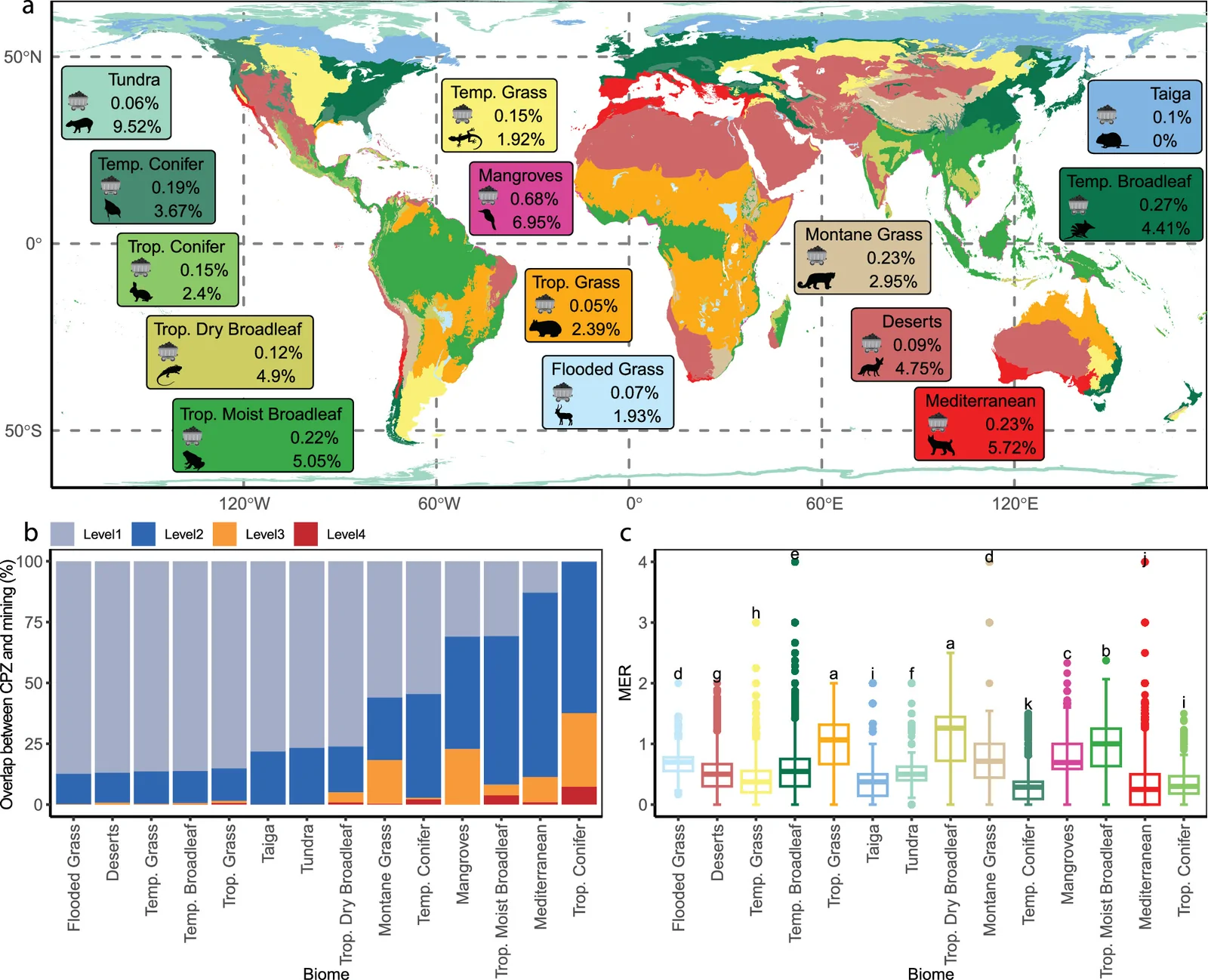
Biome-specific threats to biodiversity from mining. **a** Percentage of mining footprint (mining vehicle symbol) and the percentage of endemic vertebrate species threatened by mining (animal symbols) in each biome. Different colours on the map represent various biomes. **b** Percentage of mining footprint area in each biome that overlaps with each conservation priority zone (CPZ) level, relative to the total mining area in that biome. This panel presents proportional area summaries and does not involve inferential statistics. **c** Mean extinction risk (MER) of IUCN species impacted by mining across biomes. Letters above the boxplots indicate significant differences among biomes identified by post-hoc Dunn’s tests with Bonferroni correction (two-sided) (Kruskal–Wallis test, *χ*^2^ = 78,370, d.f. = 13, *p* < 0.001). Trop. Grass: Tropical and Subtropical Grasslands, Savannas and Shrublands; Trop. Dry Broadleaf: Tropical and Subtropical Dry Broadleaf Forests; Trop. Conifer: Tropical and Subtropical Coniferous Forests; Trop. Moist Broadleaf: Tropical and Subtropical Moist Broadleaf Forests; Temp. Broadleaf: Temperate Broadleaf and Mixed Forests; Temp. Grass: Temperate Grasslands, Savannas and Shrublands; Temp. Conifer: Temperate Conifer Forests; Mediterranean: Mediterranean Forests, Woodlands and Scrub; Montane Grass: Montane Grasslands and Shrublands; Taiga: Boreal Forests; Flooded Grass: Flooded Grasslands and Savannas; Deserts: Deserts and Xeric Shrublands; Mangroves: Mangroves; Tundra: Tundra. In **c**, boxplots (*N*_total_ = 267,127) show the 25th percentile (lower hinge), median (centre line) and 75th percentile (upper hinge). Whiskers extend to 1.5 times the interquartile range, and minima and maxima are defined as the smallest and largest values within this range, respectively. Data points beyond the whiskers are plotted individually as outliers. MER is a continuous variable ranging from [0 to 4], where 0 indicates no extinction risk and 4 represents the highest risk. CPZ is a categorical variable, with values ranging from [1 to 4], where 1 indicates no overlap with conservation priority zones and 4 represents overlap with the most critical conservation areas. All spatial data layers were reprojected to the World Behrmann equal-area projection (EPSG:54017). The endemic species icons used in (**a**) is from https://www.phylopic.org/.

Mining disproportionately impacts certain biomes. Mangroves have the highest proportional mining footprint, with 0.68% (~300 km^2^) of their total land area directly threatened by mining activities. This is followed by Temperate Broadleaf and Mixed Forests (0.27%, ~25,000 km^2^), Mediterranean (0.23%, ~6000 km^2^), Montane Grasslands and Shrublands (0.23%, ~9000 km^2^) and Tropical and Subtropical Moist Broadleaf Forests (0.22%, ~33,000 km^2^) (Fig. 2a). Despite being the smallest biomes (Supplementary Fig. 4), Mangroves and the Mediterranean biome show the highest and 3rd highest percentages of biome area affected by mining, respectively (Fig. 2a and Supplementary Fig. 4). Notably, Mangroves are among the most vulnerable and valuable ecosystems^26–29^, offering essential habitats for fish and wildlife and delivering vital ecosystem services such as carbon storage and protection against sea-level rise, tides and storm surges^36–39^. These ecosystems support millions of people in vulnerable communities worldwide, yet over half of the world’s Mangroves are at risk of collapse^36^. The overexploitation of these biomes can lead to the irreversible loss of unique and vital ecosystems and biodiversity^40–43^. Nevertheless, systematic and spatially explicit studies on the biodiversity impacts of mining in mangroves and other fragile biomes, as well as comprehensive spatial planning efforts, remain limited.

Conservation of vulnerable biomes is critical, as they support numerous endemic species. The highest percentages of endemic species threatened by mining are found in Tundra (9.5%, 2 endemic species), followed by Mangroves (6.9%, 23 endemic species), Mediterranean (5.7%, 21 endemic species), Tropical and Subtropical Moist Broadleaf Forests (5.1%, 426 endemic species) and Tropical and Subtropical Dry Broadleaf Forests (4.9%, 98 endemic species) (Fig. 2a and Supplementary Fig. 5). These endemic species, especially those with critical status, should be prioritised in conservation efforts to prevent exacerbating extinction risks^44,45^.

Across several biomes, more than 50% of the total mining footprint overlaps with CPZs. These include Tropical and Subtropical Coniferous Forests, Mediterranean, Tropical and Subtropical Moist Broadleaf Forests and Mangroves, with substantial areas in zero extinction zones (Fig. 2b). When comparing mining inside and outside protected areas within the same biome, the proportional overlap of mining footprints with CPZs, aggregated at the biome level, is significantly higher inside protected areas than outside (Kruskal–Wallis test: d.f. = 1, *χ*^2^ = 6.17, *p* = 0.01; Supplementary Fig. 6a). Polygon-level analyses further support this result, indicating that mining polygons are significantly more likely to overlap CPZs within protected areas than outside (Supplementary Table 5; estimate = − 1.36, d.f. = 267,126, *p* < 0.001, 95% CI [−1.58, −1.15]). This effect is consistent across most biomes (12 out of 14), where the probability of overlap is significantly higher within protected areas, while no significant differences are detected in Mangroves and Tropical and Subtropical Coniferous Forests (Supplementary Fig. 6b). These results indicate that mining activities disproportionately overlap with high-priority biodiversity areas within existing protected areas, highlighting potential challenges for conservation effectiveness and the implementation of mitigation strategies.

The threat of species extinction from mining, as measured by MER, is highest in Tropical and Subtropical Grasslands, Tropical and Subtropical Dry Broadleaf Forests, Tropical and Subtropical Moist Broadleaf Forests, Mangroves and Flooded Grasslands and Savannas (Fig. 2c; Kruskal–Wallis test: d.f. = 13, *χ*^2^ = 78,370, *p* < 0.001). Furthermore, when comparing mining inside and outside protected areas within the same biome, MER is significantly higher outside protected areas (Supplementary Table 6; estimate = 0.04, d.f. = 267,126, *p* < 0.001, 95% CI [0.02, 0.06]), particularly in Tropical Dry Broadleaf, Mangroves and Tropical Moist Broadleaf (Supplementary Fig. 7). This finding underscores that even areas outside protected boundaries require stringent regulation, as many unprotected landscapes still function as critical habitats for threatened species^26^.

### Future mining and biodiversity conflicts

**Fig. 3:**
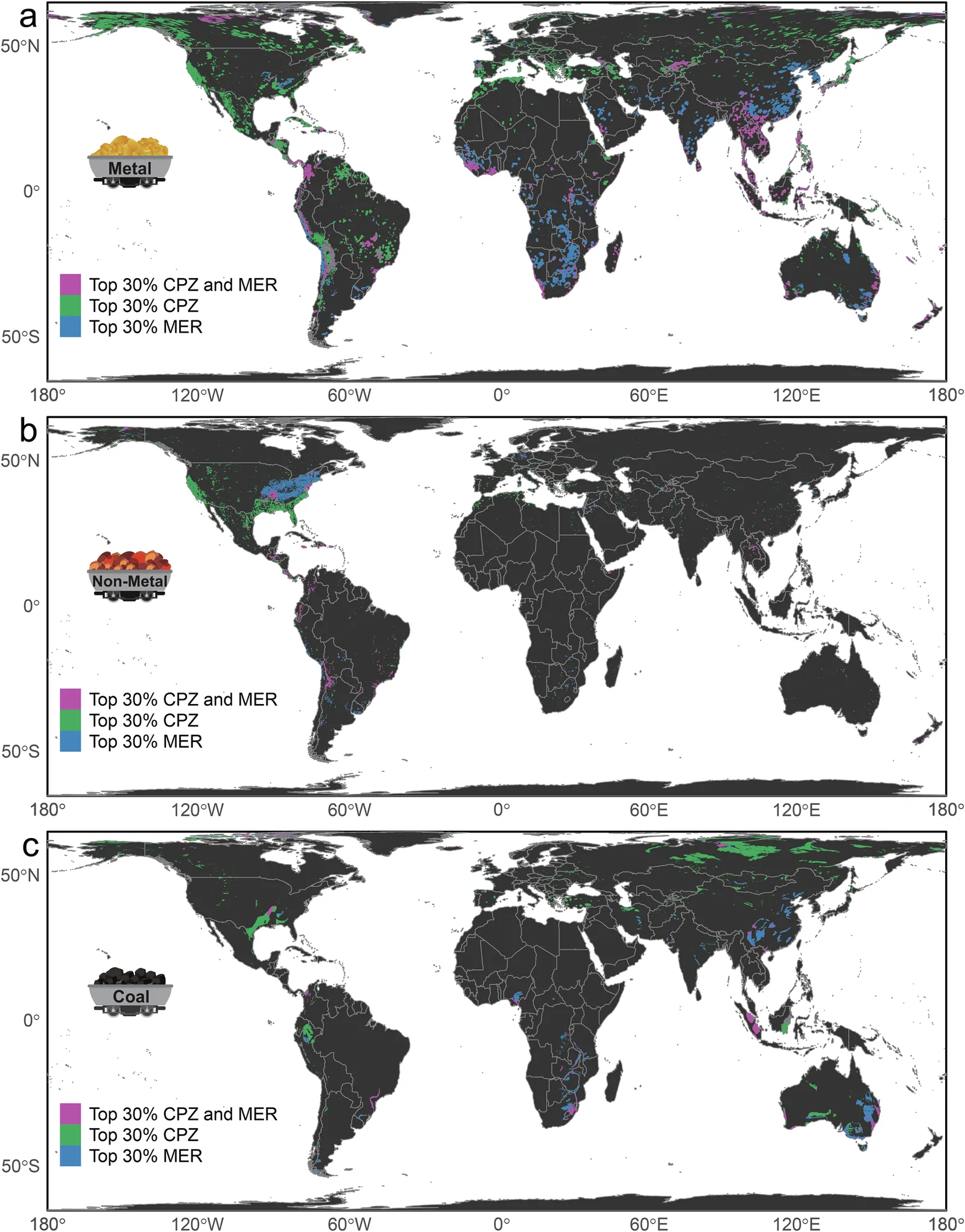
High-priority conservation areas to exclude from future mining expansion. **a** The top 30% of areas with the highest conservation priority zone (CPZ) and mean extinction risk (MER) priorities, overlapping with regions potentially exploitable for future metal mining. **b** The top 30% of areas with the highest CPZ and MER priorities, overlapping with regions potentially exploitable for future non-metal mining. **c** The top 30% of areas with the highest CPZ and MER priorities, overlapping with regions potentially exploitable for future coal mining. In each map, colours indicate CPZ-MER overlap: red indicates areas falling into both the top 30% for CPZ and MER, reflecting a high overlap of conservation priority and extinction risk; green represents areas in the top 30% for CPZ values; and blue shows regions in the top 30% for MER values. All spatial data were reprojected to the World Behrmann equal-area projection (EPSG:54017).

To assess potential conflicts between mining and biodiversity in the future, we used data from Oakleaf et al.^46^ to identify areas potentially exploitable for metals, non-metals and coal, overlapping these with CPZs and MER to pinpoint high-priority conservation areas for exclusion from future mining expansion. Our analysis demonstrates that future mining activities could significantly threaten biodiversity if left unplanned (Fig. 3). We find a strong spatial overlap between CPZ and areas potentially exploitable for metal, non-metal and coal mining. Specifically, 43% (12 million km²) of potential metal mining resources, 29% (2 million km²) of non-metal mining resources, and 37% (4 million km²) of coal mining resources overlap with CPZ (Supplementary Table 7), with many of these overlapped areas also exhibiting high MER (Fig. 3).

This spatial overlap with CPZ and MER varies substantially by mineral type (Fig. 3 and Supplementary Fig. 8). Metals pose the largest overlap (Fig. 3a), particularly in biodiversity hotspots across Southeast Asia, Australia, New Zealand, Southern Africa and South America, regions critical for global biodiversity, hosting numerous threatened species and ecosystems. The overlap between non-metal resources and biodiversity hotspots is notable in the Americas and Southeast Asia, where diverse ecosystems face significant risks (Fig. 3b). For coal, the overlap with biodiversity hotspots is primarily located in Russia, Indonesia, China, Australia, Southern Africa and parts of the Americas, including Brazil, Peru, Colombia, Ecuador, Chile and the United States (Fig. 3c).

These findings emphasise the urgency for coordinated global policies to reconcile mining expansion with biodiversity conservation, particularly as nations strive to meet ambitious energy transition targets^6,7,18^ and the GBF’s 30 × 30 goal, which aims to protect at least 30% of land and sea by 2030^3^. While the energy transition is vital for addressing climate change, the mining required for this shift must be managed responsibly to minimise its impact on ecosystems. Without proactive planning and management, mining expansion could drive significant biodiversity loss, compromising conservation efforts and threatening progress toward global biodiversity goals.

We acknowledge that the spatial distribution of non-metal mineral deposits in our analysis may reflect underlying biases in the Oakleaf et al.^46^ dataset. Countries with more extensive geological survey coverage, such as the United States, are likely overrepresented, while regions with limited or inconsistent reporting may be underrepresented. Consequently, the apparent concentration of future non-metal mineral deposits overlapping high biodiversity risk areas in North America may be partly attributable to data availability rather than actual resource distribution.

Our prioritisation maps are designed to serve as global screening tools that inform early planning rather than impose fixed development restrictions. In situations where high-risk areas cannot be avoided due to legal, economic, or geopolitical constraints, we recommend applying the mitigation hierarchy. This includes minimising impacts where possible, re-establishing or rehabilitating the damaged area on-site to restore its biodiversity, and offsetting unavoidable residual impacts.

We propose a flexible approach to applying our framework. Where planning is based on the Oakleaf et al.^46^ dataset, we recommend avoiding the top 30% of areas identified as high biodiversity risk. If avoidance is not possible, decisions should be guided by thorough site-level assessments and mitigation strategies. For developments in regions not covered by the Oakleaf dataset, we suggest benchmarking new sites against the biodiversity risk values in our maps to prioritise lower-impact alternatives.

Finally, we emphasise the need for future research to expand global deposit coverage and improve the spatial resolution and consistency of mineral resource data. Addressing current data gaps will help reduce regional biases and enable more equitable, biodiversity-sensitive resource planning worldwide.

## Discussion

### Challenges for biodiversity-inclusive mining

Mining poses one of the most persistent and geographically widespread threats to biodiversity, yet global assessments have often lacked spatial precision or relied on coarse threat proxies^11,12,16,17^. Our study advances this field by directly integrating spatially explicit mining footprints with global biodiversity datasets, including species extinction risk and CPZs, offering a more tangible and operational picture of where mining most threatens biodiversity, moving beyond coarse-scale or point-based assessments. This enables a shift from assessing potential exposure to quantifying realised spatial overlap between mining activities and biodiversity. By assessing footprint-level overlaps, our approach captures fine-scale spatial heterogeneity in biodiversity impacts^18^, avoiding potential over-estimation from coarse-resolution analyses^17^. It enables precise identification of high-risk sites and areas where conservation interventions are most needed. This also allows better attribution of impacts to specific mining sites and their operators, which is essential for guiding corporate and policy decisions aligned with frameworks such as the Taskforce on Nature-related Financial Disclosures (TNFD). Such geographic specificity and precision are particularly valuable as global conservation efforts increasingly prioritise spatially explicit strategies.

This study provides a comprehensive, spatially explicit assessment of mining’s current and future threats to global biodiversity, offering biome-specific insights to inform targeted conservation strategies. Our findings reveal that over one-third of mining areas are located within CPZs, leading to high extinction risks. These threats are particularly pronounced in countries such as China, Indonesia, Brazil, Colombia and Mexico, where inadequate legal frameworks often exacerbate the problem^14,23,47^. Without effective interventions, these threats are likely to escalate. Insufficient legal and regulatory frameworks exacerbate the issue, enabling illegal mining and expanding impacts far beyond designated mining areas^14,23^, amplifying threats to biodiversity by several orders of magnitude (Supplementary Table 4). Strengthening governance to confine mining within designated boundaries and implementing best practices to reduce impacts on biodiversity are critical to addressing these threats. Integrating biodiversity considerations into national conservation strategies, such as the National Biodiversity Strategies and Action Plans, is an essential step forward.

As global mineral demand surges, driven by infrastructure growth and the energy transition, biodiversity faces mounting pressure^7,18^. Alarmingly, critical minerals essential for green technologies^6^ have the highest overlap with CPZs and high MER values compared to other minerals such as non-metals (Supplementary Fig. 9), echoing the conclusions of Sonter et al.^11^. Without integrating biodiversity safeguards into the renewable energy agenda, these technologies risk undermining global conservation goals, directly conflicting with the GBF’s Target 8^3^. Strategies such as extending product lifetimes and increasing recycling rates of critical minerals could help reduce this demand and mitigate further biodiversity loss^47,48^.

### Solutions for biodiversity-inclusive mining

Spatial prioritisation and exclusion zones are essential for reducing biodiversity risks from mining. To meet UN SDGs and the biodiversity objectives of the Kunming-Montreal GBF adopted at COP15, mining activities should be excluded from biodiversity hotspots, areas with high conservation priority zone levels and mean extinction risk values identified in our analysis. These areas harbour irreplaceable species and provide vital ecosystem services such as carbon sequestration and flood risk reduction^36,49^. Mining-induced habitat destruction and biomass loss^10,50,51^ could significantly undermine climate mitigation efforts. Governments and industries must avoid overexploitation of fragile ecosystems, such as Mangroves and the Mediterranean, and prioritise the protection of endangered and endemic species.

Furthermore, our spatial analysis identifies specific regions where future mining would pose severe biodiversity risks, providing actionable guidance for exclusion zones. Several jurisdictions demonstrate that protective spatial planning is feasible: France’s 2024 ban on deep-sea mining within its territorial waters establishes an example for precautionary governance (https://www.legifrance.gouv.fr/jorf/id/JORFTEXT000049335073), while Botswana’s strategic environmental assessment excluded 20,000 km² of the Okavango Delta from potential mining concessions^52^. At the international level, the proposed moratorium on deep-sea mining, supported by over 20 countries and major corporations (https://deep-sea-conservation.org/), demonstrates that coordinated action can create space for industries to align extraction pathways with biodiversity targets. Implementing similar spatial exclusions for the high-priority areas identified in our study would prevent irreversible biodiversity losses while enabling responsible resource development in lower-risk zones. Implementing these measures will require action by national and subnational governments, with support from international bodies and the consent of Indigenous Peoples and local communities.

Strengthening governance and enforcement is critical in regions where mining is already established, particularly to address illegal and unregulated operations that amplify ecological damage. Advances in monitoring technologies have improved transparency and compliance. Chile’s Minera en Línea system provides real-time public tracking of mining operations^53,54^, while the Amazon Conservation Association employs satellite-based alerts via Global Forest Watch to detect illegal incursions into protected areas within days (https://www.globalforestwatch.org/?lang=en).

Regulatory frameworks must also evolve to require biodiversity integration throughout the mining lifecycle. For example, the Philippines’ Department of Environment and Natural Resources Administrative Order DAO 2022-04 mandates biodiversity assessments, mitigation plans and restoration programs from exploration through closure^55^. Additionally, participation in initiatives such as the Extractive Industries Transparency Initiative (EITI) further enhances governance by promoting public disclosure, reducing corruption risks and improving accountability^56^. These measures should be led by environmental ministries, supported by civil society organisations and judicial systems.

Advanced technology and restoration should be adopted in active mining areas, particularly where operations overlap with high-value biodiversity areas. Mining industries must also integrate nature-based solutions into restoration efforts. Alcoa’s bauxite rehabilitation program in Western Australia demonstrates this potential. Over 75% of mined land has been restored, with all pre-mining plant species re-established. (https://www.alcoa.com/australia/en/pdf/Alcoa-Australia-Rehabilitation-Factsheet.pdf?). Similarly, Vale S.A.’s Carajás Mine in Brazil integrates large-scale restoration, plant propagation and riparian rehabilitation, yielding measurable improvements in forest connectivity and wildlife corridors^57^. Despite these promising examples, systematic mine restoration remains the exception rather than the rule. Only a small fraction of inactive mines have undergone ecological rehabilitation, representing a missed opportunity to deliver lasting biodiversity gains^58^.

Well-designed biodiversity offset programs can also compensate for residual impacts that cannot be mitigated. The Ambatovy nickel mine in Madagascar, for example, offsets 2000 ha of direct forest loss through protection and restoration of 23,000 ha of equivalent habitat^59^. However, such programs must adhere to strict no-net-loss criteria and include affected communities from the outset to avoid social harm^60,61^. Implementation will depend primarily on mining companies, with government regulators enforcing standards and conservation NGOs supporting the management of offset sites.

Meaningful engagement and benefit-sharing with local and Indigenous communities are essential for effective biodiversity conservation in mining landscapes, yet remain inadequately implemented. Evidence demonstrates that community-led monitoring and co-management substantially improve conservation outcomes. At Voisey’s Bay nickel mine (Canada), a binding Impact and Benefit Agreement between the Nunatsiavut Government and mining operators ensures Inuit participation in environmental monitoring, guaranteed employment quotas, revenue-sharing and recognition of traditional land rights, demonstrating how co-management can balance biodiversity and livelihoods (https://landneedsguardians.ca/resources/from-standoff-to-stewardship). In contrast, the QMM ilmenite mine in Madagascar implemented a biodiversity offset program without adequate local consultation, resulting in loss of access to forest resources and social conflict that undermined conservation outcomes^62^. These contrasting outcomes underscore that effective conservation requires co-management arrangements with transparent benefit-sharing, incorporation of traditional ecological knowledge, recognition of community land rights, and equitable distribution of both costs and benefits. These approaches require mining companies and Indigenous and local communities to establish binding agreements, supported by governments and financial institutions.

Reducing global mineral demand is also essential to alleviate cumulative pressures on ecosystems. While site-level mitigation remains important, systemic strategies to curb global mineral demand are equally critical for reducing these broader environmental pressures. Circular-economy approaches, such as extending product lifespans, improving recycling and promoting material substitution, can significantly reduce the need for primary extraction^47,48^. The European Union’s Battery Regulation, for instance, mandates minimum recycled-content targets for key metals by 2030 (16% cobalt, 85% lead, 6% lithium and nickel), demonstrating how policy-driven circularity can directly reduce biodiversity pressure (https://eur-lex.europa.eu/eli/reg/2023/1542/oj). Achieving these reductions will require governments to set policy mandates, supported by manufacturing and recycling industries and coordinated internationally.

### Limitations and concluding remarks

While our study primarily focuses on threats to terrestrial biodiversity within the mining footprint, we recognise that its effects may extend far beyond these boundaries. Indirect threats, including habitat fragmentation from road development, illegal hunting and pollution (e.g. noise, light and chemical contamination), further threaten terrestrial and aquatic ecosystems, particularly in active and poorly regulated mining sites^9,23–25^. Quantifying these indirect impacts remains challenging and requires further research^9^. In addition, our analysis does not include freshwater and marine taxa (e.g. fishes), which have been identified as contributing substantially to global mining-related extinction risk patterns^17^. Assessing these aquatic systems requires distinct data and methods, such as hydrological, pollution and offshore extraction models. As a result, risks to aquatic biodiversity may be underrepresented in our study, highlighting the need for future research specifically integrating freshwater and marine taxa. Furthermore, emerging activities like deep-sea mining also present significant risks to marine biodiversity, though their full scope remains poorly understood^63,64^. Addressing these multifaceted threats will require concerted research efforts to elucidate and mitigate their impacts comprehensively. Furthermore, the data used in this study may underestimate the extent of mining’s threat due to the challenges in tracking illegal and small-scale mining activities^65,66^. These gaps highlight the urgent need to strengthen and harmonise biodiversity-specific monitoring within existing global mining transparency initiatives, such as the Intergovernmental Forum on Mining (IGF), the Extractive Industries Transparency Initiative (EITI) and the Initiative for Responsible Mining Assurance (IRMA). While these platforms address environmental and social safeguards, biodiversity considerations are often insufficiently integrated. Systematic, spatially explicit monitoring of mining’s impacts on biodiversity, particularly in terms of species extinction risk and critical habitat loss, would greatly enhance transparency, strengthen accountability and support conservation-aligned decision-making.

By grounding our analysis in actual spatial extents of mining, we provide a more tangible and operationally relevant picture of mining’s biodiversity impacts, one that better supports targeted conservation action and spatial planning. This level of geographic precision is particularly valuable as global conservation efforts increasingly prioritise spatially explicit strategies, such as area-based targets under the Kunming-Montreal Global Biodiversity Framework. Our findings contribute to the growing body of evidence underscoring the need for biodiversity-inclusive land-use planning and the importance of aligning mining activities with conservation priorities at both national and global scales.

## Methods

Supplementary Fig. 11 provides a visual representation of the data integration and analysis process. This workflow diagram illustrates how different data sources are combined to assess the spatial distribution of mining and its impact on biodiversity, specifically through its overlap with CPZs and mean extinction risk (MER). The diagram effectively visualises the key steps and data overlaps involved in our analysis.

### Current mining and quarrying (referred to as mining hereafter) data

To assess current biodiversity threats from mining, we compiled the most comprehensive global dataset to date on mining and quarrying land-use footprints (hereafter referred to as the mining footprint), representing the land area used for mining activities and associated infrastructure. This dataset combines polygons from Maus et al.^67^, Tang et al.^68^ and OpenStreetMap (OSM, as of March 30, 2024)^69^. Specifically, it includes 44,929 polygons from Maus et al.^67^ (accounting for 76% of the total footprint area, Supplementary Table 8), 74,548 from Tang et al.^68^ (64% of the total footprint area), and 218,101 from OSM^69^ (64% of the total footprint area), representing areas designated as quarries (https://wiki.openstreetmap.org/wiki/Tag:landuse%3Dquarry). Open data platforms like OSM have proven effective in addressing data gaps by providing valuable spatial information for monitoring land-use types^70–72^. By complementing mining footprint datasets derived from satellite imagery, such as those from Maus et al.^67^ and Tang et al.^68^, OSM^69^ enhances the spatial comprehensiveness of global mining assessments. To create a unified global mining footprint, we used vector polygon datasets from three sources: Maus et al.^67^, Tang et al.^68^ and OpenStreetMap^69^. The datasets were merged in QGIS (version 3.40.3)^73^, and spatial overlaps between mining polygons were resolved using the ‘dissolve’ function, ensuring that overlapping features across datasets were counted only once. This approach allowed us to integrate data from multiple sources without duplication. The reference years are as follows: Maus et al.^67^ and Tang et al.^68^ derive from Sentinel-2 imagery collected between 2019 and 2021, while the OSM dataset^69^ was downloaded in March 2024. While this dataset provides a high level of spatial detail, it may still underestimate mining activity, particularly small-scale and illegal operations that are harder to document.

Our analysis focuses exclusively on the direct threats of mining within the footprint, where habitat destruction, disturbance and pollution typically pose the most immediate and severe threats to biodiversity^9^. This includes both active and inactive sites, as long as the mining footprint is observable, indicating that mining has already threatened biodiversity through habitat destruction. The dataset does not account for indirect threats such as pollution, illegal hunting, or habitat fragmentation from road development, which are inherently more challenging to quantify^9^. In addition, these indirect effects vary significantly based on site-specific factors such as activity level, operational status (active or inactive), regulatory enforcement, proximity to sensitive ecosystems and overlap with other human activities (e.g. agriculture or other industrial activities), which complicates isolating mining’s contributions to broader threats. By focusing on direct habitat destruction and the most acute biodiversity risks, this approach provides a conservative estimate of mining-related impacts^9^. However, in our subsequent analysis, we explored how the impact on terrestrial biodiversity might expand if mining effects extend beyond the immediate footprint. This was achieved by establishing buffers around mining footprints and referencing the approach of Sonter et al.^11^ (see the section: Beyond the footprint: analysing mining’s broader biodiversity effects).

To explore how threats to biodiversity differ by mining type, we compiled global data on fossil fuel (primarily coal), metal and non-metal deposits (Supplementary Table 9). We intersected these deposit types with the mining footprint dataset using the st_join function from the sf package (version 1.0-16)^74^ to assign a deposit type to each mining footprint, following a similar approach to Ge et al.^43^. Deposit locations were provided as latitude and longitude coordinates, and we applied buffer zones to ensure spatial alignment with mining footprints. The buffer sizes were determined by deposit size, and where this information was unavailable, a default 500 m buffer was applied as a conservative estimate. Any mining footprint that did not overlap with a specific deposit type was categorised as unknown, accounting for approximately 43% (62,000 km^2^) of the total mining footprint (Supplementary Table 1). If a mining footprint overlapped with multiple mineral types (e.g. metal and non-metal, non-metal and coal, or metal, non-metal and coal), we retained all relevant categories. In cases where different datasets reported different commodity types at the same location, we treated these differences as complementary rather than conflicting and retained all reported commodities. This integration approach acknowledges that datasets may vary in completeness and geographic resolution, and helps to avoid overlooking relevant mineral associations.

Given the growing importance of critical minerals for the clean energy transition, we compiled data on mines and deposits for 38 key minerals (Supplementary Table 10) listed by the International Energy Agency (IEA). These minerals, primarily metals, were spatially matched to mining footprints using the same method as fuel and metal deposits.

While this method provides a global perspective on the potential biodiversity impacts of different mining commodities, several uncertainties remain. These include spatial mismatches between deposit maps and mining footprints, since the precise shapes and extents of many deposits are not available, as well as incomplete deposit and commodity records^18^. To account for these limitations, we introduced an unknown category for mining footprints where a commodity type could not be reliably assigned, representing approximately 43% of the total mining footprint area (see Supplementary Table 1). Future improvements in deposit data resolution and completeness will enable more accurate identification of commodity-specific impacts^18^.

In addition, multiple commodities are often extracted from the same deposit, particularly for non-metals and coal. By aggregating commodities into three broad categories, metals, non-metals and coal, we minimise classification uncertainty for this assessment. However, in our subsequent biodiversity impact assessment by mineral type, we acknowledge that assigning equal impacts across commodity categories within multi-commodity mining sites introduces potential bias, as different minerals may exert varying ecological pressure. Improved global reporting standards and company-level production disclosures would allow more refined attribution of biodiversity impacts, allowing for a more accurate assessment of risk across commodities.

### Conservation priority zones (CPZs)

Global biodiversity conservation priority frameworks: we selected four global biodiversity conservation frameworks, encompassing the three major groups of the irreplaceability-vulnerability gradient^75^: biodiversity hotspots (BH), Last of the Wild (LOW), Alliance for Zero Extinction (AZE) and key biodiversity areas (KBAs). These templates were selected because (i) they identify areas with the highest extinction risks and important terrestrial regions in consideration of at least one facet of biodiversity^75^; (ii) they are robust and widely used in global biodiversity modelling^11,75,76^; and (iii) the data are reliable and accessible. Among these frameworks, BH identifies areas with exceptional endemism and significant habitat loss^77^. LOW identifies areas free from industrial-scale activities and human pressures, essential for maintaining planetary life-support systems^78^. AZE focuses on sites harbouring the last remaining populations of one or more Critically Endangered or Endangered species (https://zeroextinction.org/), while KBAs represent globally important sites large enough to sustain viable populations (https://www.keybiodiversityareas.org/). BH data were obtained from Hoffman et al.^77^, LOW from Allan et al.^78^ and AZE and KBAs from BirdLife International^79^.

Categorisation of CPZs: we categorised areas into four CPZ levels based on their overlap with these global frameworks, following Yang et al.^35^:

(i) Level 4: extinction threat—AZE sites.
(ii) Level 3: high threat—areas identified by two or more important habitats (BH, LOW, KBAs).
(iii) Level 2: medium threat—areas identified by at least one important habitat (BH, LOW, KBAs).
(iv) Level 1: low threat—areas not covered by AZE, BH, LOW, or KBAs.

We further analysed which CPZs are currently covered by existing protected areas and which remain outside formal protection.

### Protected areas

Protected areas data were obtained from the March 2024 edition of the World Database on Protected Areas (WDPA)^80^. For China, additional protected area data were incorporated from the WDPA 2017 edition^81^ and the China Nature Reserve Specimen Resource Sharing Platform (https://zenodo.org/records/14875797), given recent gaps in national protected area reporting^5^. For Turkey, data were sourced from www.kursatozcan.com. It is important to note that national policies influence protected areas assignment, leading to spatial biases in protected areas coverage between countries^82^.

### Distribution of IUCN species threatened by mining and quarrying

Species range data were obtained from the IUCN Red List and BirdLife International^83,84^. Following previous studies^85,86^, we: (1) included all documented ranges, including passage and introduced areas, as these can represent ecologically critical habitats for migration corridors, seasonal refugia and ex situ conservation populations^87–89^; (2) excluded species classified as data deficient (DD) or extinct (EX, EW); and (3) identified species threatened by mining activities. The final dataset includes 3094 species across five taxa: 754 amphibians, 751 plants, 697 reptiles, 463 mammals and 429 birds. This dataset was used to assess species distributions that overlap with mining footprints and to evaluate species currently covered and not covered by protected areas.

It is important to note that, in principle, all species could be affected by habitat destruction due to mining. However, our focus here is on species most susceptible to mining threats to remain consistent with IUCN threat assessment standards.

### Endemic species in each biome

Endemic species data were collected from WWF Wildfinder to identify vertebrate species uniquely present in specific biomes and threatened by mining. While vertebrate species data were available, endemic plant species data were not accessible, limiting analysis to fauna.

### National socio-economic and governance factors

To assess the influence of national contexts on mining-related biodiversity threats, we incorporated country-level socioeconomic and governance factors. Income classifications were obtained from the World Bank (https://www.worldbank.org), which classifies countries into different groups based on gross national income per capita. As income level is an ordinal variable representing increasing levels of economic development, it was converted into a numeric ordered variable with three levels (1–3). This transformation allows income to be modelled as a continuous gradient rather than a set of independent categories. This approach reduces model complexity and facilitates the interpretation of overall trends across income levels. Among the six World Bank governance indicators (https://www.worldbank.org), we selected government effectiveness as a representative measure, as it reflects the quality of public services and policy implementation that can influence environmental management. In addition, a Spearman correlation analysis showed strong intercorrelations (∣*r*_s_∣ ≥ 0.7) between government effectiveness and the other governance indicators (Supplementary Fig. 12), supporting its use as a proxy. Therefore, income level and government effectiveness were used to represent national socio-economic and governance factors in this study.

### Mining overlap with CPZs and IUCN species extinction risks

All spatial data layers were reprojected to the World Behrmann equal-area projection (EPSG:54017). We assessed the overlap of current mining surfaces with CPZs and IUCN species distributions using the sf package (version 1.0-16)^74^. We assigned the value of CPZ that intersects with the mining footprint and calculated the mean extinction risk (MER) of imperilled species for this footprint. MER was calculated by averaging assigned values from IUCN Red List categories within each polygon:

1 $${{{\rm{MER}}}}=\frac{{N}_{{{{\rm{LC}}}}}\times 0+{N}_{{{{\rm{NT}}}}}\times 1+{N}_{{{{\rm{VU}}}}}\times 2+{N}_{{{{\rm{EN}}}}}\times 3+{N}_{{{{\rm{CR}}}}}\times 4}{{N}_{{{{\rm{LC}}}}}+{N}_{{{{\rm{NT}}}}}+{N}_{{{{\rm{VU}}}}}+{N}_{{{{\rm{EN}}}}}+{N}_{{{{\rm{CR}}}}}}$$

where MER represents the mean extinction risks for mining driven imperilled species, and *N*_LC_, *N*_NT_, *N*_VU_, *N*_EN_ and *N*_CR_ represent the number of least concern (LC), near threatened (NT), vulnerable (VU), endangered (EN) and critically endangered (CR) species, respectively. For calculating MER, we assigned values to each IUCN Red List category (LC: 0, NT: 1, VU: 2, EN: 3, CR: 4) according to Meng et al.^86^.

### Beyond the footprint: analysing mining’s broader biodiversity effects

Mining impacts often extend well beyond the immediate extraction footprint through pollution, habitat fragmentation and associated land-use changes^9–11^. However, quantifying these indirect effects is challenging due to site-specific variations such as different regulatory enforcement and operational practices. Rather than attempting to predict indirect effects across diverse contexts, we conducted a sensitivity analysis to examine how biodiversity threats escalate as mining impacts extend beyond the footprint into surrounding areas. Specifically, we evaluate scenarios where mining threats spill over into surrounding habitats at distances of 5, 10, 15, 20, 25 and 30 km, under the assumption that habitats within these buffers are entirely degraded and ecologically non-functional, which is a conservative worst-case scenario that establishes an upper bound on potential impacts. The selected distances encompass the range of off-site effects reported in previous studies. For example, Sonter et al.^11^ applied a 10 km buffer as a conservative estimate of mining’s impacts, while de Castro Pena et al.^90^ identified indirect effects typically extending 5–10 km from mining sites. This analysis aims to quantify how stringent regulatory measures can help reduce or prevent the broader threats of mining on biodiversity.

For each buffer distance, we recalculated the overlap with CPZs and mean extinction risk (MER). This approach quantifies how threats scale with distance and highlights the potential benefits of stringent regulations that effectively contain mining activities within their designated operational boundaries. The results of this sensitivity analysis are presented in Supplementary Table 4.

### Mapping current mining and biodiversity hotspots

We created global bivariate maps to visualise the relationship between mining footprints and biodiversity hotspots, specifically focusing on CPZs and MER. Importantly, CPZ and MER capture distinct dimensions of biodiversity, ranging from ecosystem-level to species-level values, and are therefore not necessarily spatially congruent. To do so, we reclassified mining footprint areas into ten equal categories based on quantiles. In contrast, CPZs were maintained in their original categorical structure (levels 1-4), reflecting increasing conservation priority. Similarly, for MER, we applied natural breaks corresponding to ecologically meaningful intervals (0–1, 1–2, 2–3, 3–4), which provides an interpretable representation of extinction risk gradients. We then scaled all values and overlaid the mining footprint areas with CPZ and MER separately, producing two maps: CPZ versus mining footprint areas and MER versus mining footprint areas. These maps highlight areas where mining overlaps with high-risk biodiversity regions, offering crucial insights for both government and private sector decision-makers. By identifying these intersections, we can guide efforts to mitigate or avoid biodiversity loss while contributing to the achievement of Sustainable Development Goals (SDGs) and the Global Biodiversity Framework (GBF) targets.

While all mining activities pose some threat on local biodiversity, our study emphasises areas where the potential threats are most severe. These findings can help inform targeted conservation actions aimed at minimising biodiversity loss and aligning with global biodiversity protection commitments, such as the SDGs and GBF.

### Statistical analysis

Variability in threats across biomes: to assess biodiversity threats across different biomes, we used the Kruskal–Wallis test to compare the MER across 14 biomes^91^. Post-hoc Dunn’s tests were then applied to identify significant pairwise differences. In addition, we calculated the percentage of mining footprint within each biome and analysed the overlap with CPZs. This approach allowed us to identify biomes where mining poses the greatest risk, providing further evidence of the disproportionate threats that mining activities can pose to certain ecosystems. We also calculated the difference between protected and unprotected areas to illustrate biome-level patterns.

Comparing mining impacts (CPZ) inside and outside protected areas: to evaluate whether mining overlap with CPZs at the biome level differs between protected and unprotected areas, we performed a Kruskal–Wallis rank-sum test comparing the proportion of mining footprints overlapping CPZs between protection categories across biomes (Supplementary Fig. 6a), using the agricolae package (version 1.3-7)^92^. As the analysis is conducted at the biome scale, with a single aggregated value per biome and protection category, formal statistical comparisons within individual biomes are not possible. To further evaluate the consistency of the observed pattern across biomes and enable statistical inference, we conducted a complementary analysis at the mining footprint (polygon) level. Specifically, we tested whether mining polygons are more likely to overlap CPZ within protected areas (PAs) than outside PAs. Each mining polygon was treated as a binary observation, coded as 1 if it intersected CPZ and 0 otherwise. We fitted a binomial generalised linear model (GLM) with a logit link, including protection status (Protection), biome (Biome) and their interaction. We also considered the potential effects of income level and government effectiveness (ge). However, these two variables are strongly correlated and capture overlapping dimensions of socioeconomic development (Supplementary Fig. 12). Including both variables in a single model would introduce multicollinearity and complicate the interpretation of their individual effects. Given prior evidence that national income influences biodiversity outcomes^30^, we included income level in the main model. To additionally assess whether governance exerts an independent effect, we fitted a separate supporting model including ge instead of income. This two-model approach allows for robust and interpretable inference by isolating the effects of income and governance, while avoiding instability arising from collinearity. The model formulations were as follows:

2 $${{{\rm{Main\; model}}}}:{{{\rm{Intersect\; CPZ}}}} \sim {{{\rm{Protection}}}}\times {{{\rm{Biome}}}}+{{{\rm{Income\; level}}}}$$

3 $${{{\rm{Supporting\; model}}}}:{{{\rm{Intersect\; CPZ}}}} \sim {{{\rm{Protection}}}}\times {{{\rm{Biome}}}}+{{{\rm{ge}}}}$$

To evaluate overall differences between protected and unprotected areas, we used estimated marginal means (EMMs) derived from the main model using the emmeans package (version 1.8.8)^93^, averaging the effect of protection status across all biomes (Supplementary Table 5). Statistical significance was determined using Wald tests. To assess biome-specific differences, we conducted pairwise comparisons of protected versus unprotected polygons within each biome using EMMs conditioned on biome identity (Supplementary Fig. 6b). The marginal effect of income level was evaluated from its main effect in the main model, while the marginal effect of ge was assessed from its main effect in the supporting model (Supplementary Table 5).

Comparing mining impacts (MER) inside and outside protected areas: To test whether mining within PAs poses greater extinction risk than mining outside PAs within the same biome, we fitted a generalised linear model with MER as the response variable, including protection status, biome and their interaction. Income level and ge were also considered as potential predictors. Due to the strong correlation between income and ge, only income was included in the main model, while a supporting model replaced income with ge to assess governance effects independently. The model formulations were as follows:

4 $${{{\rm{Main\; model}}}}:{{{\rm{MER}}}} \sim {{{\rm{Protection}}}}\times {{{\rm{Biome}}}}+{{{\rm{Income\; level}}}}$$

5 $${{{\rm{Supporting\; model}}}}:{{{\rm{MER}}}} \sim {{{\rm{Protection}}}}\times {{{\rm{Biome}}}}+{{{\rm{ge}}}}$$

Protection status (inside vs. outside PAs) and biome were treated as categorical fixed effects, with an interaction term to assess biome-specific differences in protection effects. Country-level socio-economic and governance variables were included as covariates to control for the national context. Because MER values ranged from 0 to 4 and were right-skewed, a small constant (0.001) was added to ensure positivity, and a Gamma GLM with a log-link function was applied, appropriate for continuous, right-skewed response data. To evaluate overall differences between protected and unprotected areas, we used estimated marginal means (EMMs) derived from the main model, averaging the effect of protection status across all biomes (Supplementary Table 6). Statistical significance was determined using Wald tests. Biome-specific differences were assessed using pairwise comparisons of protected versus unprotected polygons within each biome using EMMs conditioned on biome identity (Supplementary Fig. 7). The marginal effect of income level was derived from the main model, while the marginal effect of ge was derived from the supporting model (Supplementary Table 6).

All statistical analyses were performed in R version 4.4.0^94^ and RStudio version 2023.12.1+402^95^.

### Future mining threat projections

To assess potential future mining threats, we analysed 1-km² resolution indices from Oakleaf et al.^46^ that identified areas potentially exploitable for metals, non-metals and coal. These areas were categorised into six levels of development potential for each mining type in Oakleaf et al.^46^. For this study, which focuses on identifying overlaps between future exploitable areas and biodiversity hotspots, we did not consider the development potential but instead included all areas identified as exploitable. The data were therefore converted into spatial occurrence maps representing the global distribution of these minerals. This approach enabled us to quantify the potential threats of metal, non-metal and coal mining on biodiversity from future expansion.

To forecast future biodiversity threats from mining, we projected areas that could be mined in the future and mapped them against CPZs and MER data. By integrating these projections, we identified regions where future mining is likely to exacerbate biodiversity risks, should mining activities occur in these areas. This analysis highlights regions in urgent need of conservation interventions, aligning with international conservation goals. Specifically, we identified the top 30% of areas where mining is potentially exploitable but overlaps with either the highest CPZ or MER. These areas represent key priorities for future mitigation and protection efforts and should be avoided in future mining activities.

For the future distribution of critical minerals, the dataset used^46^ provides information for metals, non-metals and coal, but does not distinguish critical minerals as a separate category. Although we compiled data on current mines and deposits for 38 key critical minerals (Supplementary Table 10), generating a global prospectivity map by overlapping these deposits with potential future mining areas was not feasible. This limitation arises because many critical mineral datasets are point-based and largely based on currently exploited deposits rather than undiscovered or prospective resources. This highlights a key data gap: spatially explicit, globally consistent information on prospective critical mineral deposits is currently lacking. Addressing this gap will be important for assessing future expansion of critical minerals and their potential interactions with biodiversity conservation, particularly in the context of increasing demand for minerals required for the clean energy transition.

### Reporting summary

Further information on research design is available in the Nature Portfolio Reporting Summary linked to this article.

## Supplementary information

Supplementary Information

Reporting Summary

Transparent Peer Review File

## Data Availability

All data are provided in the Methods and Supplementary Information. Current mining footprint data were sourced from Maus et al.^67^, Tang et al.^68^ and OpenStreetMap (OSM, as of March 30, 2024)^69^. Data on future mining potential areas were obtained from Oakleaf et al.^46^. Biodiversity hotspots data were sourced from Hoffman et al.^77^; Last of the Wild data from Allan et al.^78^; and data on Alliance for Zero Extinction sites and Key Biodiversity Areas from BirdLife International^79^. Protected areas data were collected from the World Database on Protected Areas (WDPA)^80,81^, the China Nature Reserve Specimen Resource Sharing Platform (https://zenodo.org/records/14875797) and www.kursatozcan.com. Species range data were retrieved from the IUCN Red List and BirdLife International^83,84^. Endemic species data from the WWF Wildfinder database can be requested from WWF. Country income levels and management effectiveness were obtained from the World Bank (https://www.worldbank.org). Data on the distribution of deposits for various minerals are listed in Supplementary Table 9. The data generated in this study and required to reproduce all analyses and figures have been deposited in Zenodo under accession code https://doi.org/10.5281/zenodo.21769493^96^.

## Code availability

The code used to process the data, conduct the analyses and generate the figures is available in Zenodo under accession code https://doi.org/10.5281/zenodo.21769493^96^.
