# Supplementary material for: Post-2020 conservation priorities are threatened by global mining
Source: Nat Commun. 2026 Sep 21;17:11007. doi: 10.1038/s41467-026-77626-6 (PMC13687551; doi:10.1038/s41467-026-77626-6)
Supplement: Supplementary file 1 [file 41467_2026_77626_MOESM1_ESM.pdf]

Supplementary Information

Post-2020 conservation priorities are threatened by global mining

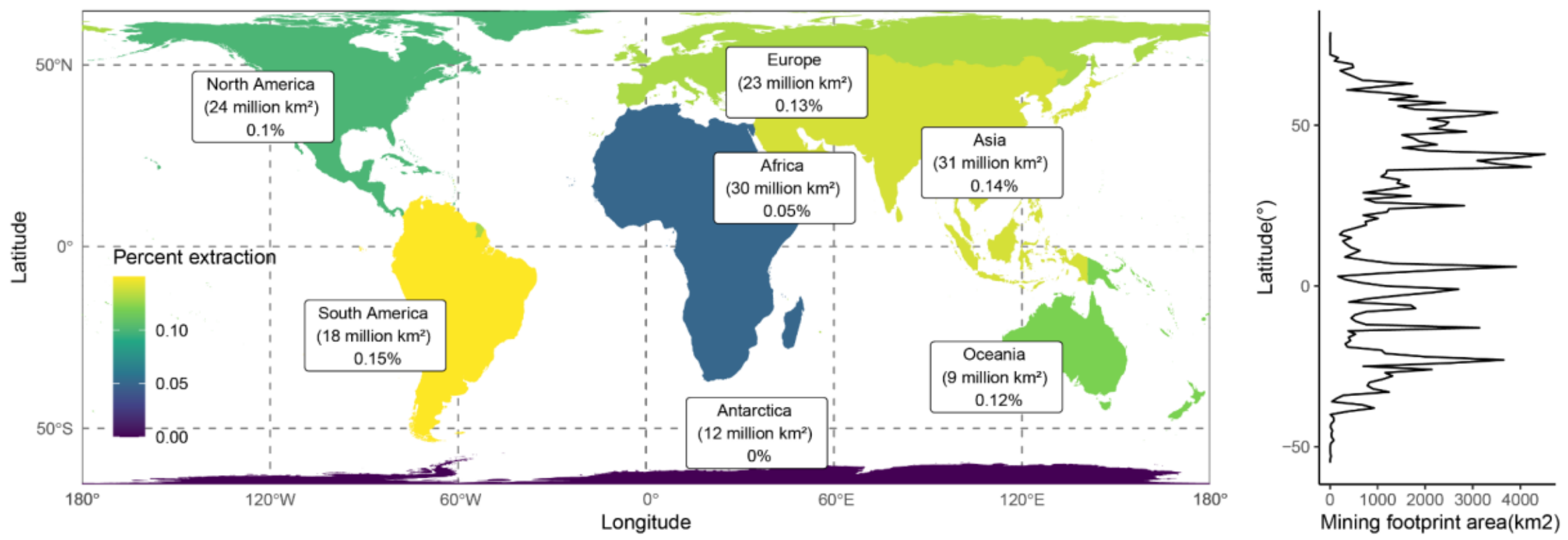

Supplementary Figure 1: Global distribution of mining footprints by continent. Mining footprints are aggregated by country and displayed along a latitudinal gradient. The figure highlights regional variations in mining intensity and extent.

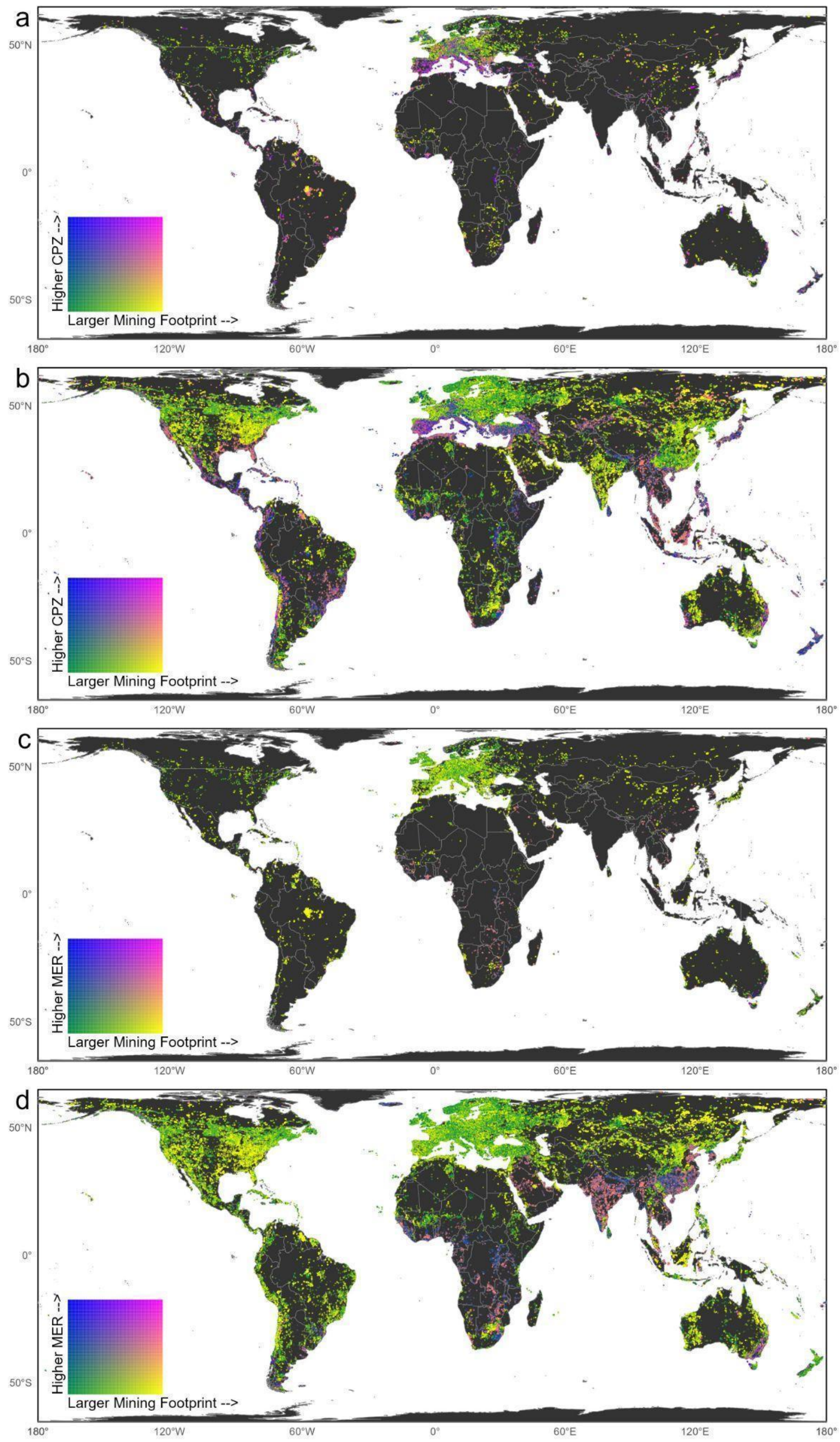

Supplementary Figure 2: **Global distribution of mining threats to conservation priority**

zones (CPZs) and mean extinction risk (MER) of IUCN Red List species. **a**, Spatial relationship between CPZs and the size of mining footprint polygons within protected areas. **b**, Spatial relationship between MER and the size of mining footprint polygons within protected areas. **c**, Spatial relationship between CPZs and the size of mining footprint polygons outside protected areas. **d**, Spatial relationship between MER and the size of mining footprint polygons outside protected areas. Colours represent: green, low conservation importance and small polygon size; blue, high conservation importance and small polygon size; yellow, low extinction risk and large polygon size; magenta, high extinction risk and large polygon size. To create the maps, mining footprints were reclassified based on quartiles and scaled without units (Methods). CPZ retained its original categorical classification (levels 1-4). MER was classified using natural breaks corresponding to ecologically meaningful intervals (0-1, 1-2, 2-3, 3-4). The original mining footprints ranged from (0 - 2,000] km^2. MER and CPZ are unitless. MER is a continuous variable ranging from [0 - 4], where 0 indicates no extinction risk and 4 represents the highest risk. CPZ is a categorical variable, with values ranging from [1 - 4], where 1 indicates no overlap with conservation priority zones and 4 represents overlap with the most critical conservation areas. All spatial data layers were reprojected to the World Behrmann equal-area projection (EPSG:54017).

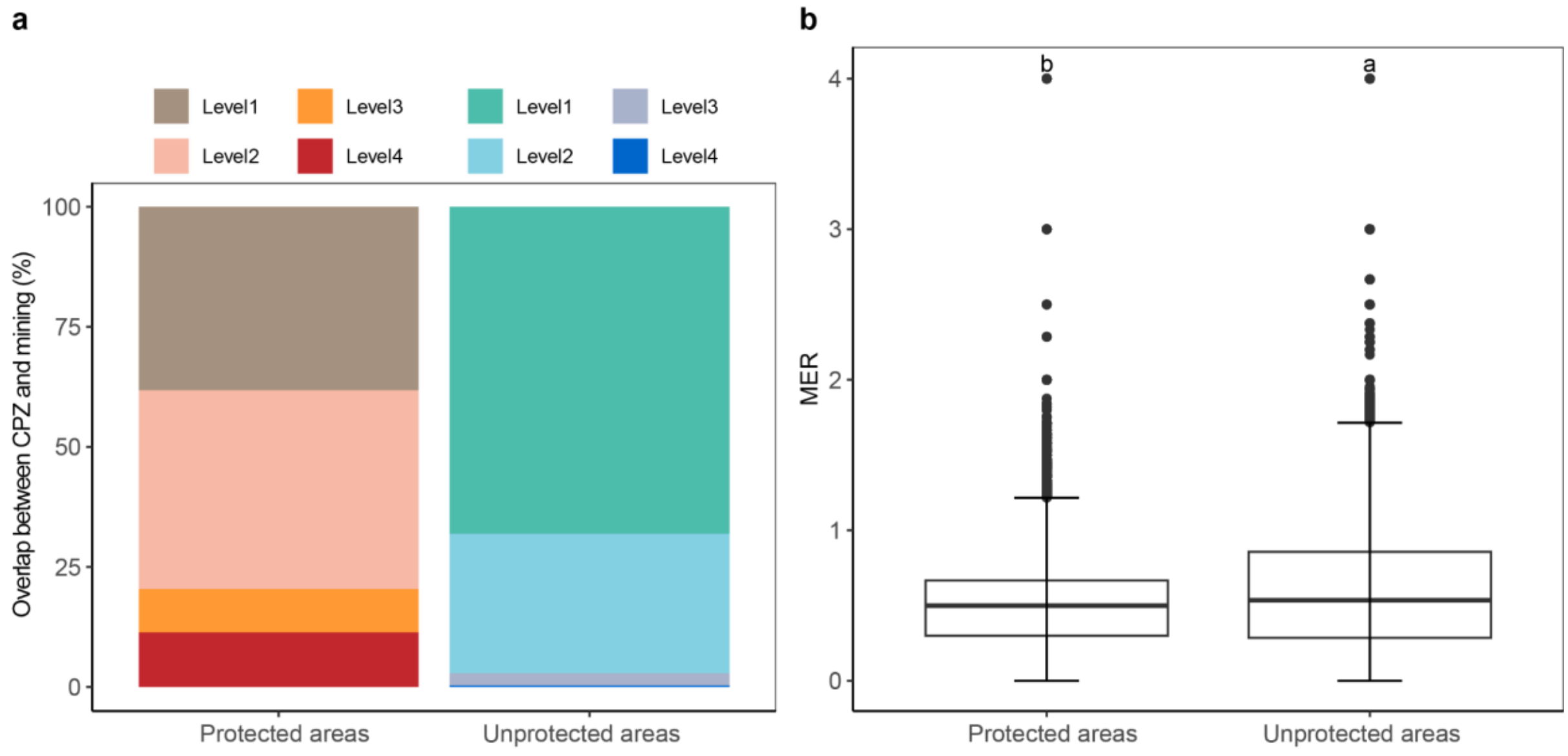

Supplementary Figure 3: Impacts of mining footprint on conservation priority zones (CPZs) and mean extinction risk (MER) inside and outside protected areas. a, Percentage of mining footprint overlapping with CPZ levels, categorised by location inside and outside protected areas. b, Comparison of MER between areas inside and outside protected areas (Kruskal-Wallis test, $\chi2 = 815$, d.f. = 1, $p < 0.01$). In b, boxplots ($N_{total} = 267{,}127$) show the 25th percentile (lower hinge), median (middle line) and 75th percentile (upper hinge). The vertical bars show the interquartile range times 1.5, and data points beyond these bars are outliers. CPZ is a categorical variable, with values ranging from [1 - 4], where 1 indicates no overlap with conservation priority zones and 4 represents overlap with the most critical conservation areas. The MER ranges from [0, 4], where 0 indicates no extinction risk and 4 represents the highest risk.

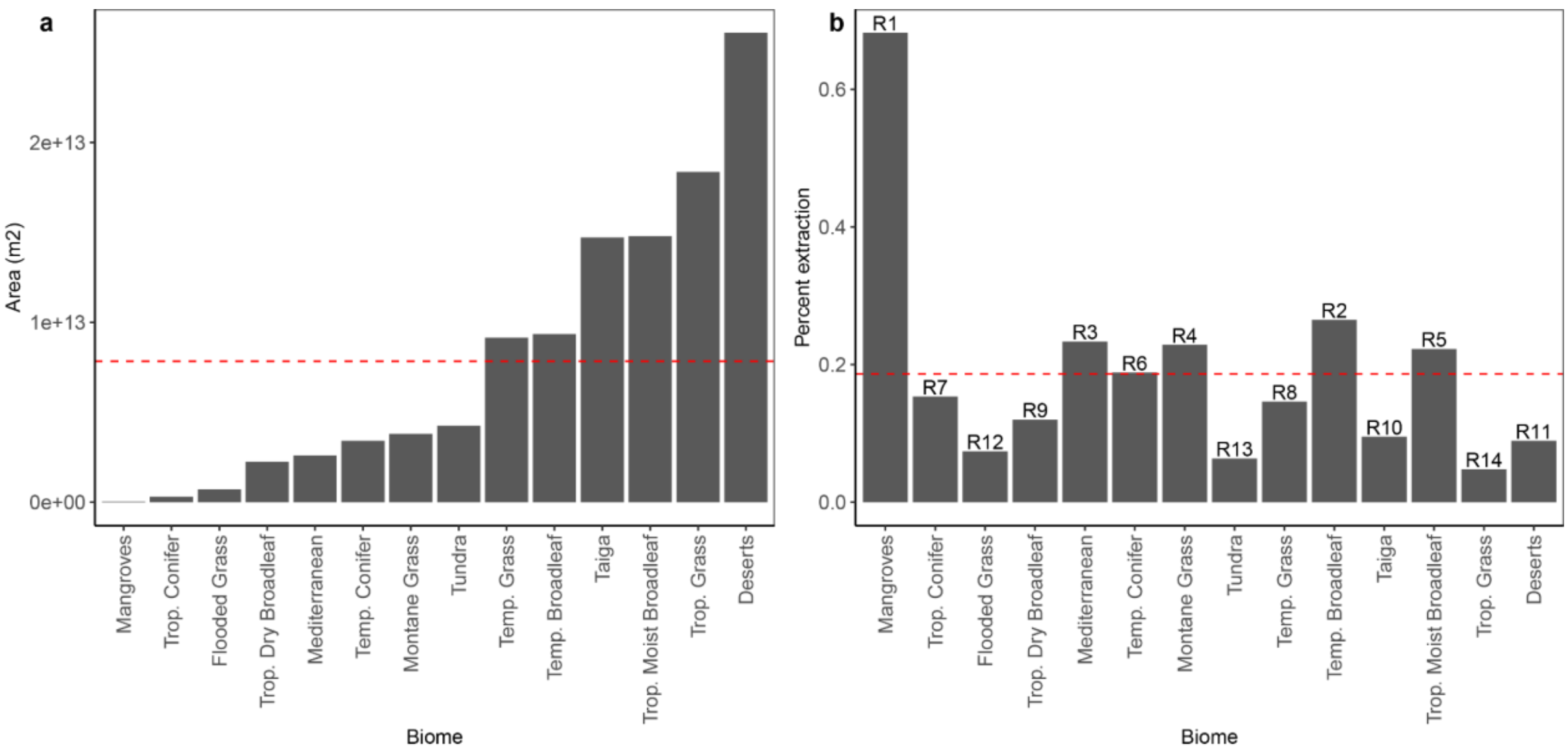

Supplementary Figure 4: Biome areas and the proportion of mining footprints within each biome. a, Total area of each biome. **b**, Percentage of mining footprint within each biome. Categories R1 to R14 indicate percentage ranks, with R1 representing the highest proportion of mining footprint and R14 the lowest. Red dashed lines denote the mean biome area or the mean extraction percentage. **Trop. Grass**: Tropical & Subtropical Grasslands, Savannas & Shrublands; **Trop. Dry Broadleaf**: Tropical & Subtropical Dry Broadleaf Forests; **Trop. Conifer**: Tropical & Subtropical Coniferous Forests; **Trop. Moist Broadleaf**: Tropical & Subtropical Moist Broadleaf Forests; **Temp. Broadleaf**: Temperate Broadleaf & Mixed Forests; **Temp. Grass**: Temperate Grasslands, Savannas & Shrublands; **Temp. Conifer**: Temperate Conifer Forests; **Mediterranean**: Mediterranean Forests, Woodlands & Scrub; **Montane Grass**: Montane Grasslands & Shrublands; **Taiga**: Boreal Forests; **Flooded Grass**: Flooded Grasslands & Savannas; **Deserts**: Deserts & Xeric Shrublands; **Mangroves**: Mangroves; **Tundra**: Tundra.

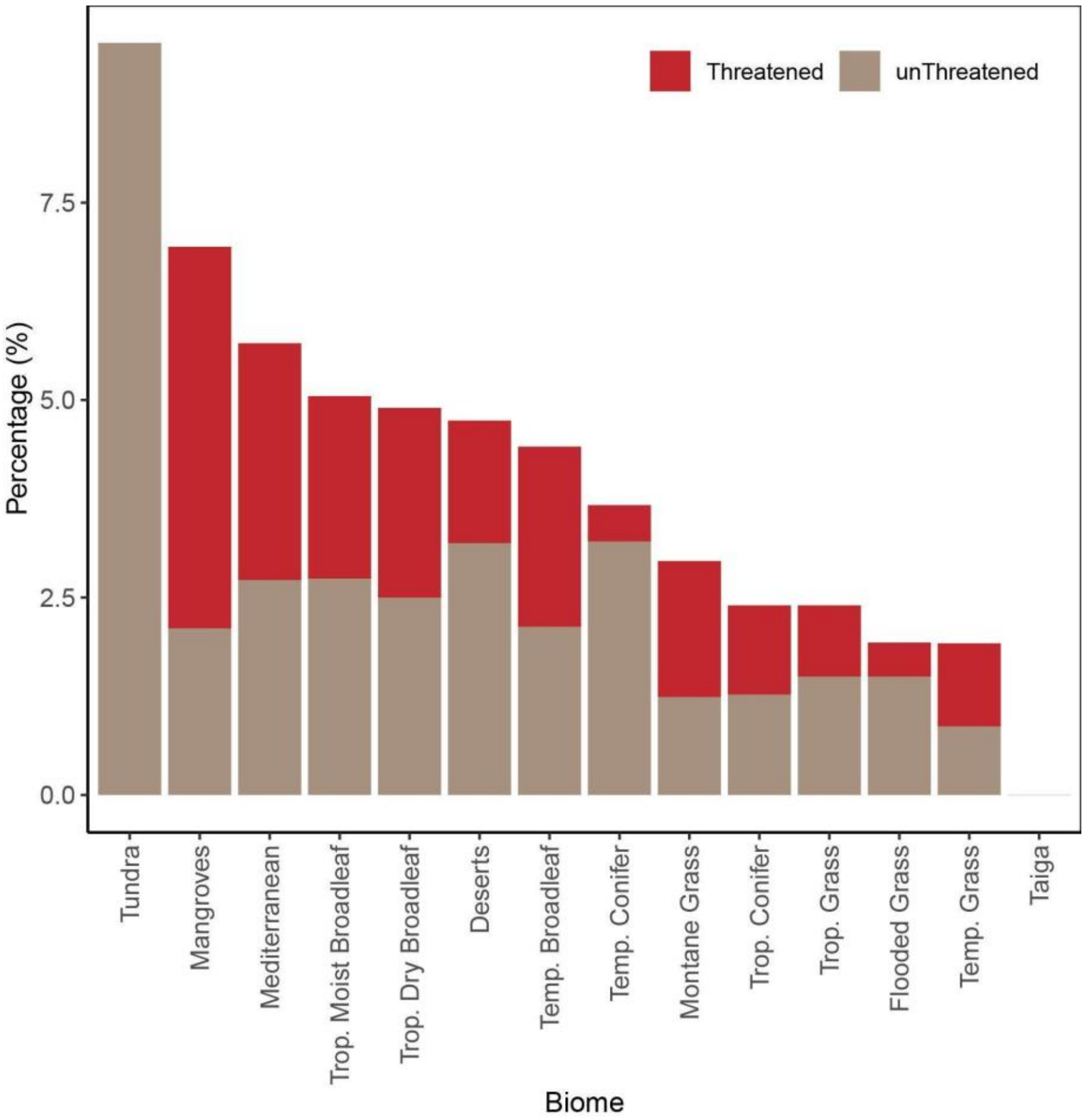

Supplementary Figure 5: Percentages of endemic vertebrate species among all vertebrate species impacted by mining within each biome. Bar chart showing the proportion of endemic vertebrate species relative to all species affected by mining activities in each biome. Biomes are ranked in descending order based on the percentage of endemic species impacted. **Trop. Grass**: Tropical & Subtropical Grasslands, Savannas & Shrublands; **Trop. Dry Broadleaf**: Tropical & Subtropical Dry Broadleaf Forests; **Trop. Conifer**: Tropical & Subtropical Coniferous Forests; **Trop. Moist Broadleaf**: Tropical & Subtropical Moist Broadleaf Forests; **Temp. Broadleaf**: Temperate Broadleaf & Mixed Forests; **Temp. Grass**: Temperate Grasslands, Savannas & Shrublands; **Temp. Conifer**: Temperate Conifer Forests; **Mediterranean**: Mediterranean Forests, Woodlands & Scrub; **Montane Grass**: Montane Grasslands & Shrublands; **Taiga**: Boreal Forests; **Flooded Grass**: Flooded Grasslands & Savannas; **Deserts**: Deserts & Xeric Shrublands; **Mangroves**: Mangroves; **Tundra**: Tundra.

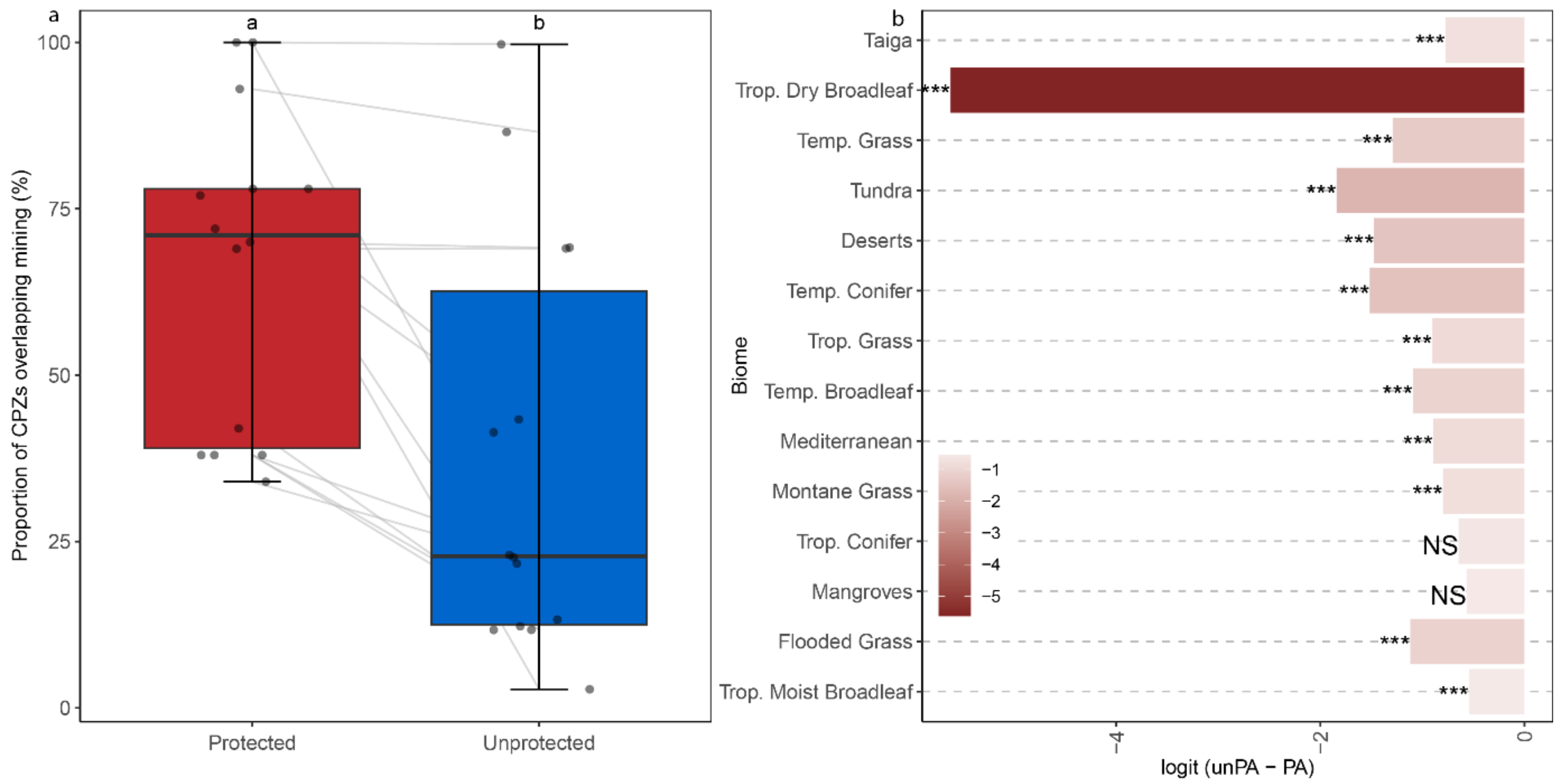

Supplementary Figure 6: Comparison of mining overlap with conservation priority zones (CPZs) inside and outside protected areas across biomes. a, Boxplots ($N_{total} = 28$) show the distribution of the proportion of mining footprints overlapping CPZs across global biomes, separated by protection status (red: protected areas; blue: unprotected areas). Each point represents a biome. The central line indicates the median, boxes show the interquartile range, and whiskers extend to 1.5× the interquartile range. Letters above boxplots indicate significant differences between groups based on Kruskal-Wallis post-hoc tests ($\chi 2 = 6.17$, d.f. = 1, $p = 0.01$). Grey lines connect paired values for the same biome between protected and unprotected areas. **b**, Bar plots showing differences in the probability of mining polygons overlapping CPZ between protected areas and unprotected areas across biomes. Negative (red) values indicate a higher probability of overlap within protected Areas. Estimates were obtained from estimated marginal means of binomial GLMs (Protection × Biome + Income or ge). Statistical significance was assessed using Wald tests (*p < 0.05; **p < 0.01; **p < 0.001). CPZ is a categorical variable, with values ranging from [1 - 4], where 1 indicates no overlap with conservation priority zones and 4 represents overlap with the most critical conservation areas. **Trop. Grass**: Tropical & Subtropical Grasslands, Savannas & Shrublands; **Trop. Dry**

Broadleaf: Tropical & Subtropical Dry Broadleaf Forests; **Trop. Conifer**: Tropical & Subtropical Coniferous Forests; **Trop. Moist Broadleaf**: Tropical & Subtropical Moist Broadleaf Forests; **Temp. Broadleaf**: Temperate Broadleaf & Mixed Forests; **Temp. Grass**: Temperate Grasslands, Savannas & Shrublands; **Temp. Conifer**: Temperate Conifer Forests; **Mediterranean**: Mediterranean Forests, Woodlands & Scrub; **Montane Grass**: Montane Grasslands & Shrublands; **Taiga**: Boreal Forests; **Flooded Grass**: Flooded Grasslands & Savannas; **Deserts**: Deserts & Xeric Shrublands; **Mangroves**: Mangroves; **Tundra**: Tundra. **unPA**: unprotected. **PA**: protected. Detailed model outputs for panels c-d are provided in Supplementary Table 5.

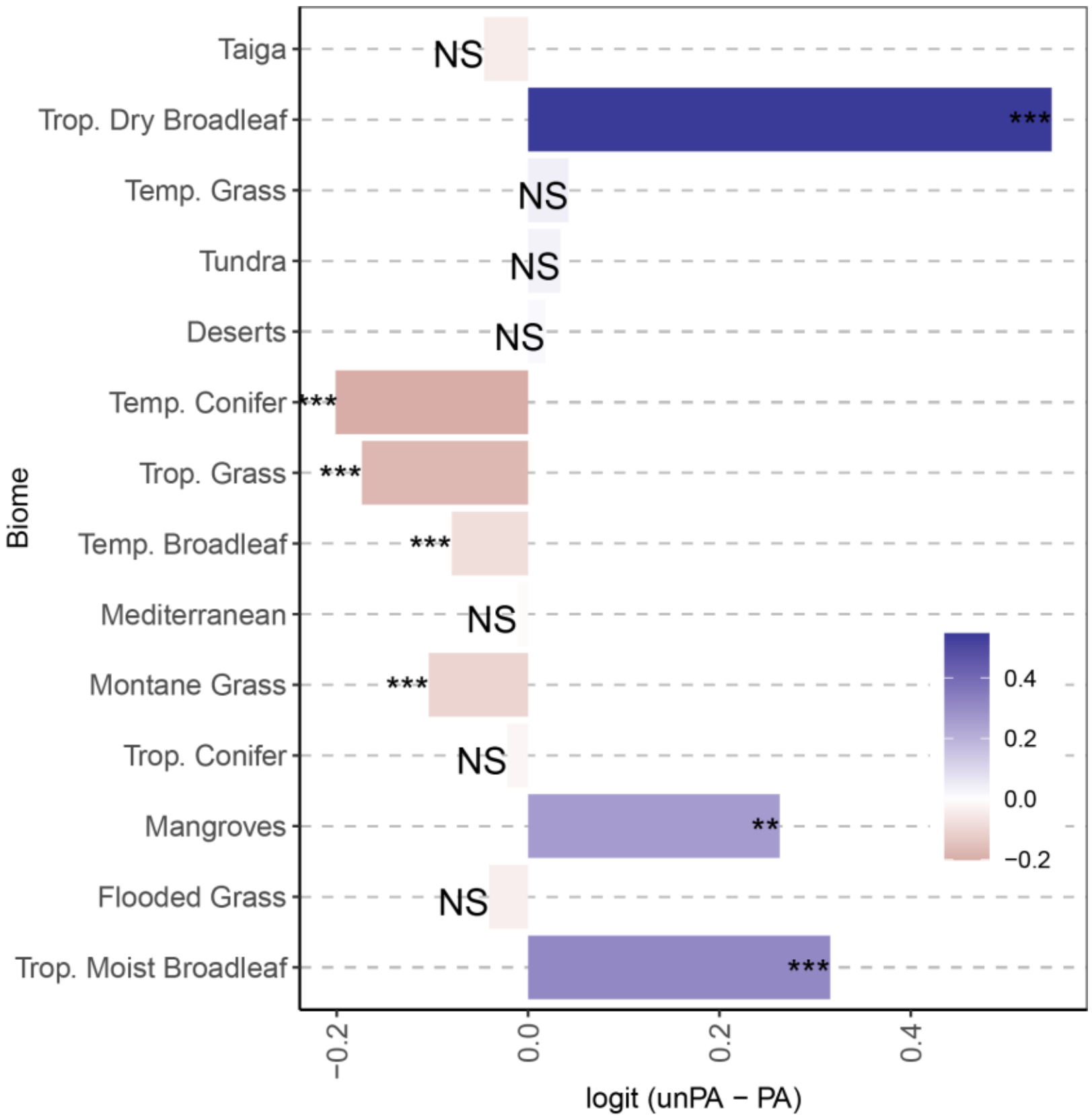

Supplementary Figure 7: Biome-specific differences in mining-related mean extinction risk (MER) between protected and unprotected areas. Positive values (blue) indicate higher MER in unprotected areas, while negative values (red) indicate higher MER in protected areas. Estimates were obtained from estimated marginal means of Gamma GLMs (Protection × Biome + Income or ge). Statistical significance was assessed using Wald tests (**p* < 0.05; ***p* < 0.01; ***p* < 0.001). **unPA**: unprotected. **PA**: protected. Detailed model outputs for panels c-d are provided in Supplementary Table 6.

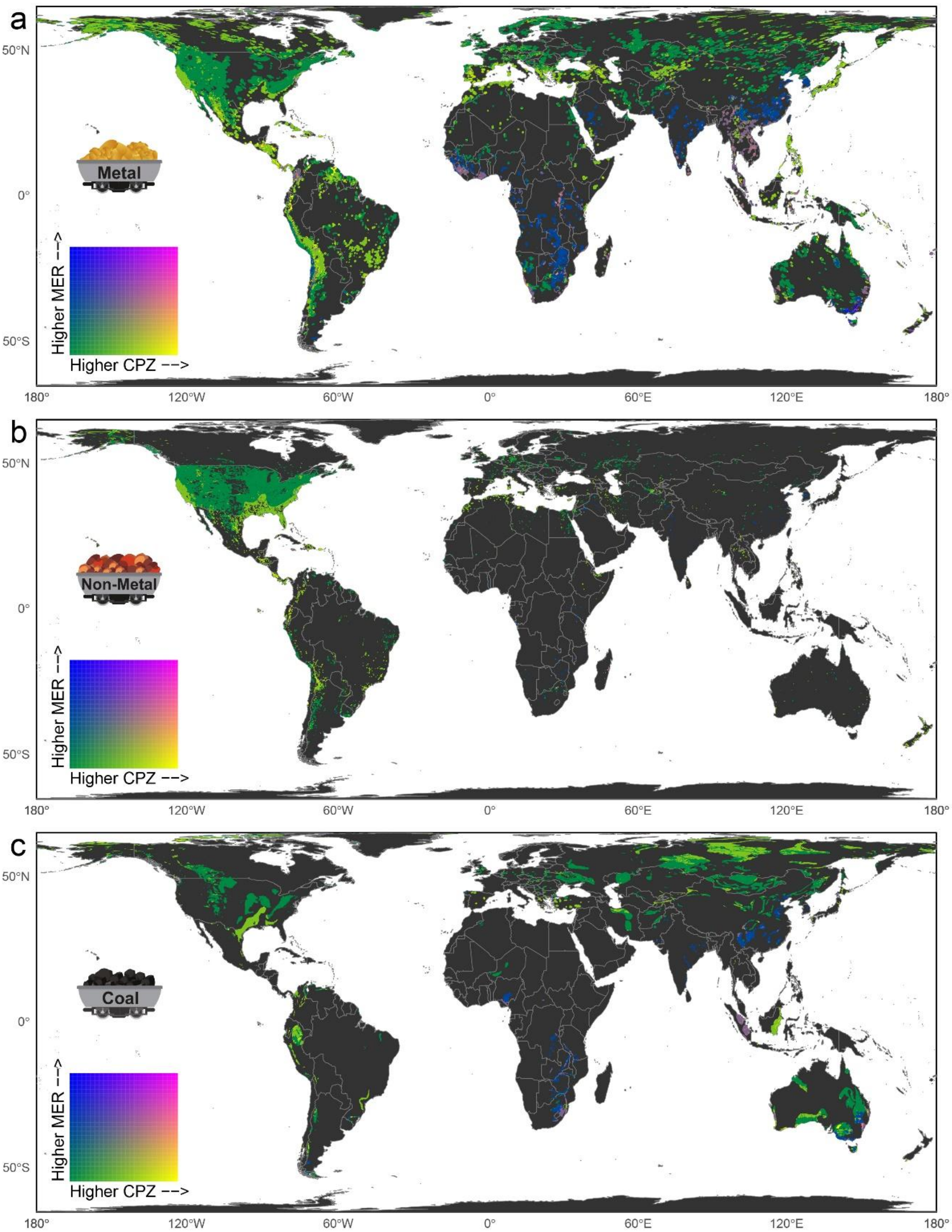

Supplementary Figure 8: Conservation priority zones (CPZs) versus the mean extinction risk (MER) of IUCN Red List species concerning regions potentially exploitable for the future. a, CPZs versus MER in regions potentially exploitable for future metal mining. b, CPZs

versus MER in regions potentially exploitable for future non-metal mining. c, CPZs versus MER in regions potentially exploitable for future coal mining. MER and CPZ are unitless. MER is a continuous variable ranging from [0 - 4], where 0 indicates no extinction risk and 4 represents the highest risk. CPZ is a categorical variable, with values ranging from [1 - 4], where 1 indicates no overlap with conservation priority zones and 4 represents overlap with the most critical conservation areas. All spatial data layers were reprojected to the World Behrmann equal-area projection (EPSG:54017).

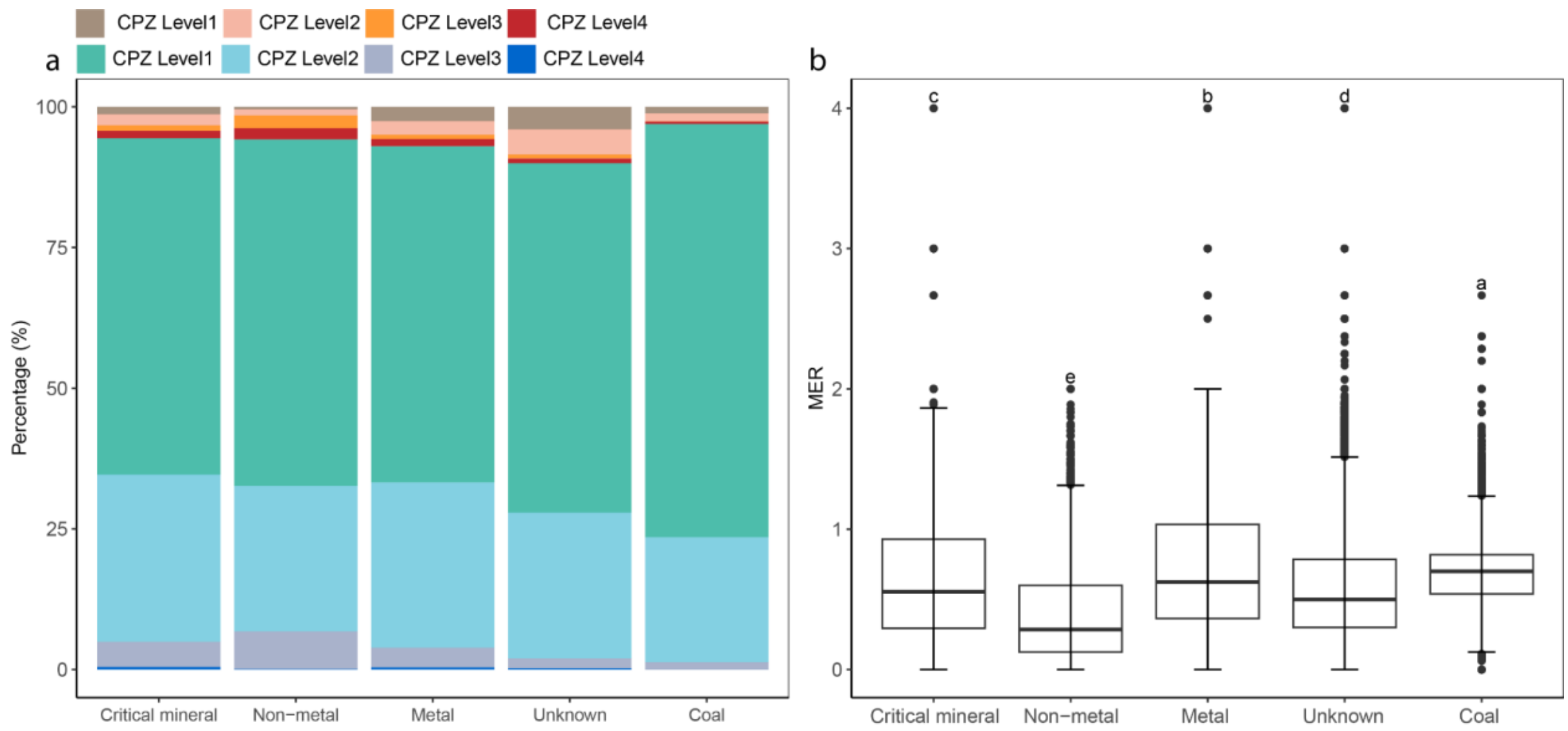

Supplementary Figure 9: Threats of mineral extraction (fuel, metal, critical minerals for renewable energy, and non-metal) to conservation priority zone (CPZs), mean extinction risk (MER) of IUCN red list Species. a, Percentage overlap between mining footprints for different mineral types and various CPZ levels. **b,** Mean extinction risk of IUCN species threatened by mining for different minerals (Kruskal-Wallis test, $\chi2 = 3{,}980$, d.f. = 4, $p < 0.01$). In panel b, boxplots ($N_{\mathrm{total}} = 280{,}430$) show the 25th percentile, median and 75th percentile. The vertical bars show the interquartile range times 1.5 and datapoints beyond these bars are outliers. The MER ranges from [0, 4], where 0 indicates no extinction risk and 4 represents the highest risk. CPZ is a categorical variable, with values ranging from [1 - 4], where 1 indicates no overlap with conservation priority zones and 4 represents overlap with the most critical conservation areas. This figure corresponds to current mining footprints (main text Fig. 1)

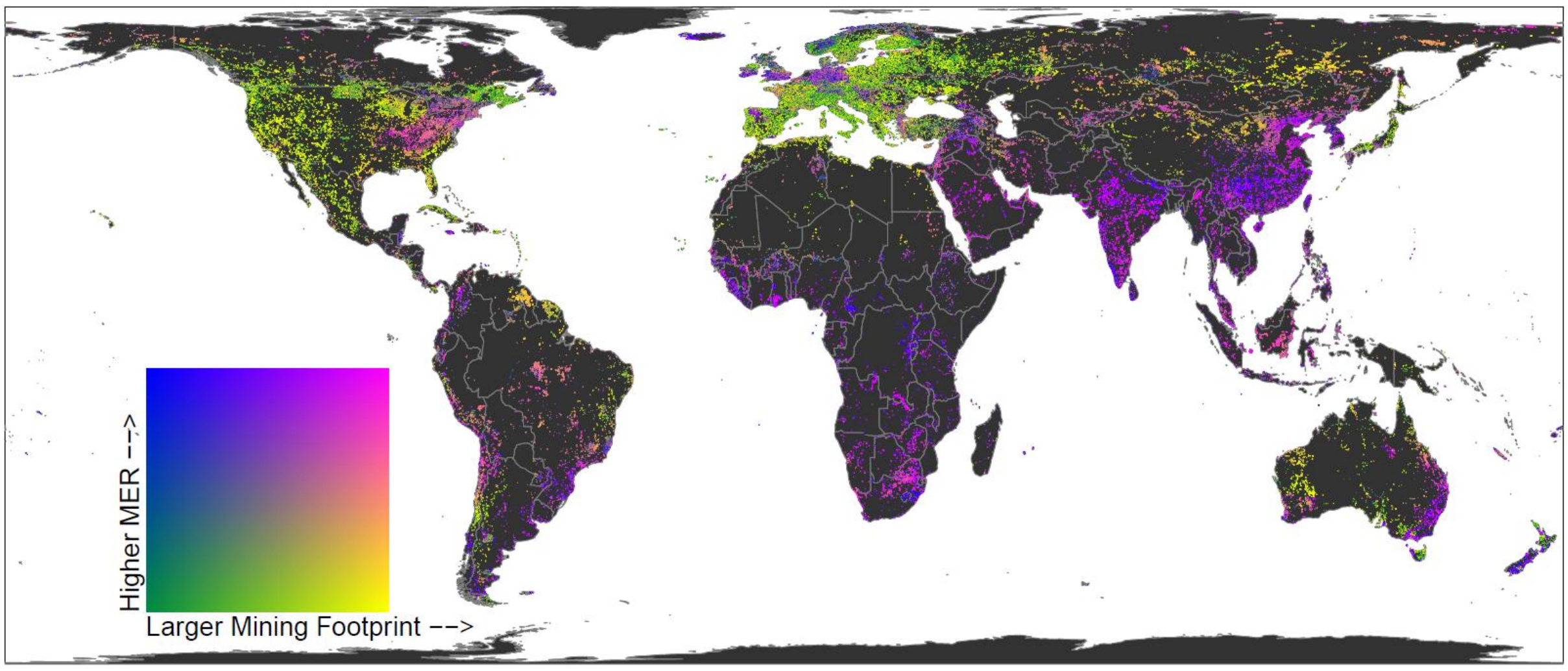

Supplementary Figure 10: Quantile-based classification of mean extinction risk (MER) across global mining footprints, provided for comparison with the natural-break classification in Fig. 1b. This approach highlights top-ranked MER risk areas but may include substantial variation within classes due to the right-skewed distribution of MER values.

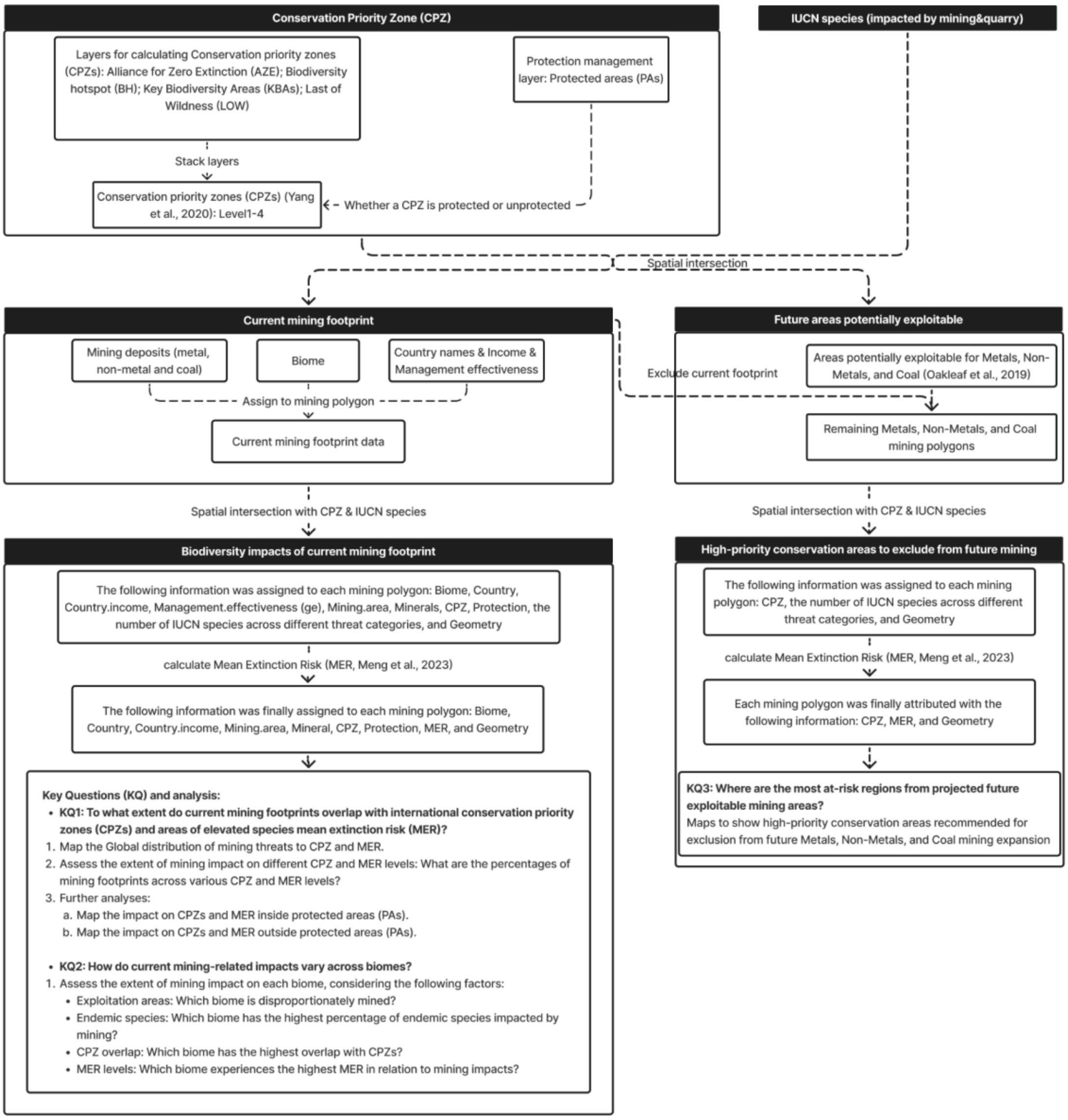

Supplementary Figure 11: Schematic diagram of the data integration and analysis workflow.

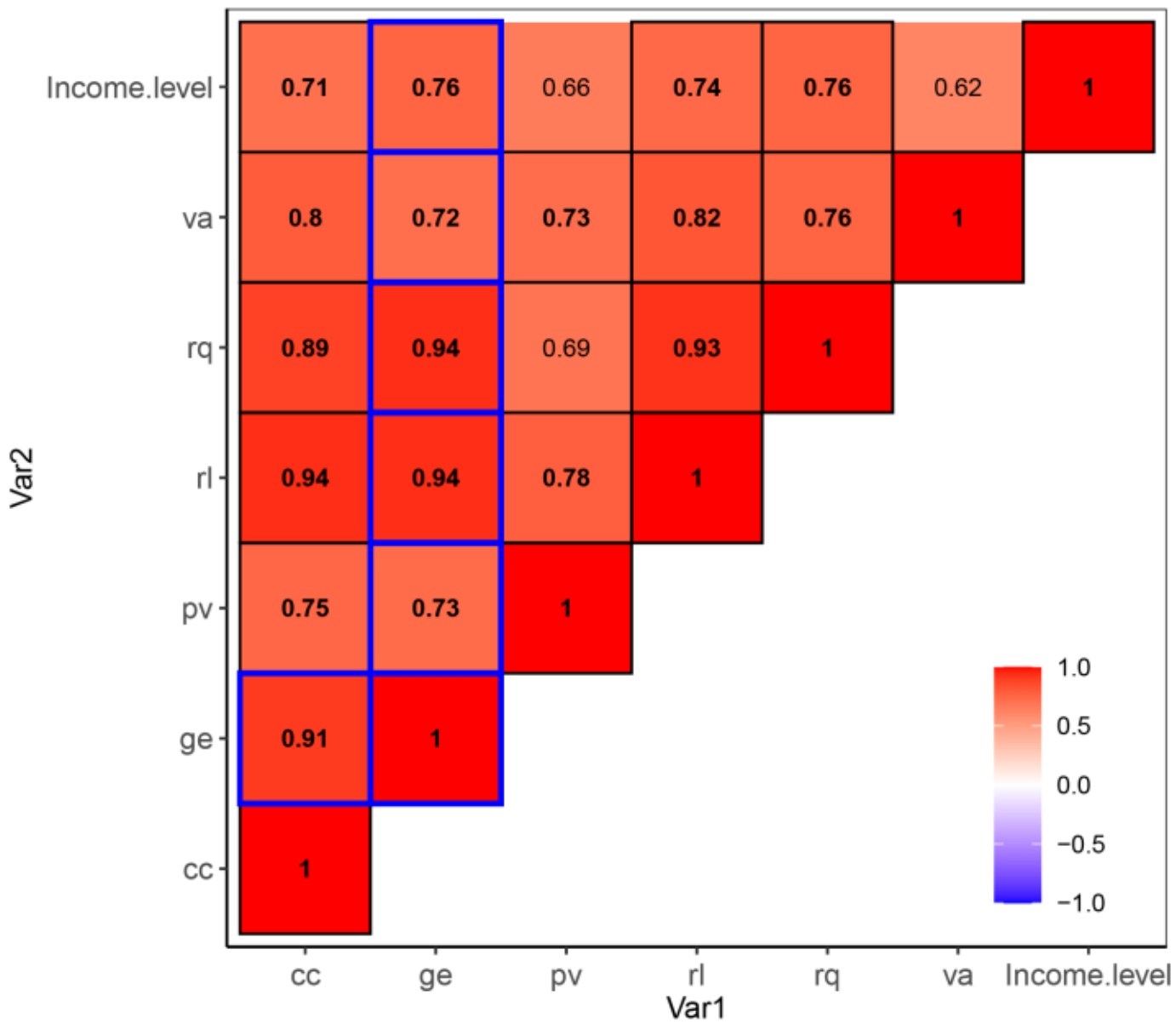

Supplementary Figure 12: Correlation among selected variables. cc, ge, pv, rl, rq, and va are Worldwide Governance Indicators (WGI), representing Control of Corruption, Government Effectiveness, Political Stability, Rule of Law, Regulatory Quality, and Voice and Accountability, respectively. Income level reflects the level of economic development. All variables were obtained from the World Bank**.**

Supplementary Table 1: Categorisation of mining footprint by mineral type. Fuels: includes coal, lignite, and peat, with coal being the primary focus. Metals: includes all metallic minerals. Non-metals: encompasses all minerals except fuels and metals. Unknown: refers to mining footprints that did not overlap with a specific deposit type, leaving their categorization undefined. Note that the total does not sum to 100%, as a single mining polygon may extract more than one mineral type (e.g., both metal and non-metal).

**Mineral**	**Area (km^2)**	**Percentage of area (%)**
Fuels	24,000	16
Metals	55,000	38
Non-metals	20,000	14
Critical minerals	39,000	27
Unknown	62,000	43

Supplementary Table 2: Percentage of mining footprint overlapping with different conservation priority zones (CPZs) and mean extinction risk (MER) globally. CPZs are classified into 4 levels, from no overlap (level 1) to overlap with a zero extinction zone (level 4), the most critical conservation area. MER is scaled from 0 (no extinction risk) to 4 (highest extinction risk). AZE: Alliance for Zero Extinction. BH: Biodiversity Hotspots, LOW: Last of the Wild. KBAs: Key Biodiversity Areas.

**Category**	**Sub-Category**	**Explanation**	**Area (km^2)**	**Percentage (%)**
CPZ level	Level 1	Areas not covered by AZE, BH, LOW, or KBAs	96,000	66
	Level 2	Areas identified by at least one important habitat (BH, LOW, KBAs)	43,000	30
	Level 3	Areas identified by two or more important habitats (BH, LOW, KBAs)	4,000	3
	Level 4	Areas covered by AZE	2,000	1

**Category**	**Sub-Category**	**Explanation**	**Area (km^2)**	**Percentage (%)**
MER	[0 - 1]	The extinction risk equals to areas where all species are classified as IUCN Least Concern (LC) to Near Threatened (NT)	122,000	84
	(1 - 2]	The extinction risk equals to areas where all species are classified as IUCN NT to Vulnerable (VU)	23,000	16
	(2 - 3]	The extinction risk equals to areas where all species are classified as IUCN VU to Endangered (EN)	100	0.1
	(3 - 4]	The extinction risk equals to areas where all species are classified as IUCN EN to Critically Endangered (CR)	4	0.003

Supplementary Table 3: Percentage of threatened species among all species impacted by mining in each biome and globally. The table presents the number and proportion of threatened species within each biome, highlighting variations in mining-related biodiversity risks across biomes. The total species count across all biomes exceeds the global species count, as some species occur in multiple biomes.

**Biome**	**Threatened species (n)**	**Total species (n)**	**Percentage (%)**
Tundra	6	34	18
Boreal Forests	9	47	19
Flooded Grasslands & Savannas	29	138	21
Temperate Conifer Forests	40	173	23
Deserts & Xeric Shrublands	158	564	28
Temperate Grasslands, Savannas & Shrublands	52	168	31
Mangroves	110	343	32
Tropical & Subtropical Coniferous Forests	61	192	32

Biome	Threatened species (n)	Total species (n)	Percentage (%)
Tropical & Subtropical Grasslands, Savannas & Shrublands	152	474	32
Montane Grasslands & Shrublands	155	436	36
Tropical & Subtropical Dry Broadleaf Forests	193	508	38
Temperate Broadleaf & Mixed Forests	110	262	42
Mediterranean Forests, Woodlands & Scrub	146	343	43
Tropical & Subtropical Moist Broadleaf Forests	661	1,492	44

Globe	1,057	2,311	46

Supplementary Table 4: Sensitivity analysis of mining threat escalation with distance from current extraction footprint. Surface area overlap with conservation priority zones (CPZs) and mean extinction risk (MER) for threatened species under scenarios where mining impacts extend 5, 10, 15, 20, 25, or 30 km beyond current footprint boundaries. Values represent cumulative impacts assuming complete habitat degradation within each buffer distance, relative to the impacts observed within the current mining footprint.. CPZs range from level 1 (no overlap with priority conservation areas) to level 4 (overlap with Alliance for Zero Extinction sites). MER ranges from 0 (no extinction risk) to 4 (critically endangered species present). Threat multipliers (relative to current footprint) demonstrate the potential biodiversity benefits of regulatory measures that contain mining within designated boundaries. Note: For MER (proportion-based metric reflecting species threat composition), multipliers may decrease at larger buffer distances as the expanding area incorporates habitats with lower proportions of highly threatened species, illustrating spatial heterogeneity in species extinction risk.

**Variable**	**CPZ**	**CPZ**	**CPZ**	**CPZ**	**MER**	**MER**	**MER**	**MER**
**Category**	**Level1**	**Level2**	**Level3**	**Level4**	**[0-1]**	**(1-2]**	**(2-3]**	**(3-4]**
5 km/footprint	55	50	47	28	53	53	110	566
10 km/footprint	113	117	113	72	109	136	544	1552
15 km/footprint	165	175	159	118	157	216	1131	2075
20 km/footprint	209	230	206	163	197	304	527	1603
25 km/footprint	252	279	247	200	236	381	461	290
30 km/footprint	286	324	285	240	269	439	751	347

Supplementary Table 5: Results of binomial generalised linear models (two-sided Wald z-tests) assessing the effects of protection, biomes, income level and government effectiveness (ge) on site-level overlap with conservation priority areas (CPZ) (n = 267,127). The main model was specified as: Intersect CPZ ~ Protection × Biome + Income level. A supporting model was fitted as: Intersect CPZ ~ Protection × Biome + ge, solely to evaluate the effect of governance. The supporting model was conducted separately due to the strong correlation between income level and ge (Supplementary Figure 12). Pairwise contrasts between protected and unprotected areas within each biome were derived using estimated marginal means. Each biome yielded a single pairwise contrast (protected vs. unprotected); no adjustment for multiple comparisons was therefore applied. Exact p-values are reported throughout.

**Term**	**Estimate**	**Std.error**	***P*.value**	**Model**
**Overall (marginal): Unprotected - Protected (EMM)**	**-1.36**	**0.11**	**0.00**	Main
(Intercept)	-0.24	0.06	0.00	
**Unprotected**	**-1.47**	**0.06**	**0.00**	
**Income.level**	**-0.18**	**0.01**	**0.00**	
Mangroves	2.30	0.30	0.00	
Mediterranean	4.50	0.12	0.00	
Montane Grass	1.02	0.09	0.00	
Taiga	-0.63	0.11	0.00	
Temp. Broadleaf	-0.35	0.06	0.00	
Temp. Conifer	1.37	0.06	0.00	
Temp. Grass	0.12	0.09	0.17	
Trop. Conifer	5.26	1.00	0.00	
Trop. Dry Broadleaf	5.56	1.00	0.00	
Trop. Grass	0.08	0.08	0.30	
Trop. Moist Broadleaf	1.61	0.07	0.00	
Tundra	0.83	0.16	0.00	
Flooded Grass	-1.14	0.23	0.00	

Unprotected:Flooded Grass	0.35	0.27	0.18	
Unprotected:Mangroves	0.89	0.31	0.00	
Unprotected:Mediterranean	0.58	0.12	0.00	
Unprotected:Montane Grass	0.67	0.10	0.00	
Unprotected:Taiga	0.70	0.11	0.00	
Unprotected:Temp. Broadleaf	0.38	0.06	0.00	
Unprotected:Temp. Conifer	-0.05	0.07	0.52	
Unprotected:Temp. Grass	0.18	0.09	0.05	
Unprotected:Trop. Conifer	0.83	1.03	0.42	
Unprotected:Trop. Dry Broadleaf	-4.15	1.00	0.00	
Unprotected:Trop. Grass	0.57	0.08	0.00	
Unprotected:Trop. Moist Broadleaf	0.93	0.07	0.00	
Unprotected:Tundra	-0.37	0.17	0.03	
(Intercept)	-0.59	0.05	0.00	Supporting
**ge**	**-0.49**	**0.01**	**0.00**	

Supplementary Table 6: Results of Gamma generalised linear models (two-sided Wald z-tests) assessing the effects of protection, biomes, income level and government effectiveness (ge) on site-level mean extinction risk (MER) (n=267,127). The main model was specified as: MER ~ Protection × Biome + Income level. A supporting model was fitted as: MER ~ Protection × Biome + ge, solely to evaluate the effect of governance. The supporting model was conducted separately due to the strong correlation between income level and ge (Supplementary Figure 12). Pairwise contrasts between protected and unprotected areas within each biome were derived using estimated marginal means. Each biome yielded a single pairwise contrast (protected vs. unprotected); no adjustment for multiple comparisons was therefore applied. Exact p-values are reported throughout.

Term	Estimate	Std.error	*P*.value	Vars
**Overall (marginal): Unprotected - Protected (EMM)**	**0.04**	**0.01**	**0.00**	Main
(Intercept)	0.35	0.02	0	
**Unprotected**	**0.02**	**0.02**	**0.33**	
**Income.level**	**-0.45**	**0.00**	**0**	
Mangroves	0.12	0.08	0.13	
Mediterranean	-0.38	0.02	0	
Montane Grass	0.34	0.03	0	
Taiga	0.00	0.03	0.9	
Temp. Broadleaf	0.32	0.02	0	
Temp. Conifer	-0.22	0.02	0	
Temp. Grass	-0.19	0.03	0	
Trop. Conifer	-0.45	0.07	0	
Trop. Dry Broadleaf	0.10	0.06	0.12	

Trop. Grass	0.57	0.03	0	
Trop. Moist Broadleaf	0.20	0.02	0	
Tundra	0.33	0.06	0	
Flooded Grass	0.28	0.06	0	
Unprotected:Flooded Grass	-0.06	0.06	0.35	
Unprotected:Mangroves	0.25	0.09	0	
Unprotected:Mediterranean	-0.03	0.02	0.19	
Unprotected:Montane Grass	-0.12	0.03	0	
Unprotected:Taiga	-0.06	0.03	0.06	
Unprotected:Temp. Broadleaf	-0.10	0.02	0	
Unprotected:Temp. Conifer	-0.22	0.02	0	
Unprotected:Temp. Grass	0.02	0.03	0.44	
Unprotected:Trop. Conifer	-0.04	0.08	0.61	
Unprotected:Trop. Dry Broadleaf	0.53	0.06	0	
Unprotected:Trop. Grass	-0.19	0.03	0	
Unprotected:Trop. Moist Broadleaf	0.30	0.02	0	
Unprotected:Tundra	0.02	0.06	0.79	
(Intercept)	-0.68	0.02	0	Supporting
**ge**	**-0.02**	**0.00**	**0**	

Supplementary Table 7: Percentage of areas potentially exploitable for future mining overlapping with conservation priority zones (CPZs) and mean extinction risk (MER) globally. CPZs are classified into 4 levels, ranging from no overlap (level 1) to overlap with the most critical conservation areas (level 4). MER is scaled from 0 (no extinction risk) to 4 (highest extinction risk). MER and CPZ are unitless.

Variable	Category	Area (km^2)	Percentage of area (%)	Mineral
CPZ	Level 1	16 million	57	Metal
CPZ	Level 2	10 million	38	Metal
CPZ	Level 3	1 million	4	Metal
CPZ	Level 4	0.3 million	1	Metal
MER	[0 - 1]	22 million	82	Metal
MER	(1 - 2]	5 million	18	Metal
MER	(2 - 3]	66,000	0.2	Metal
MER	(3 - 4]	9,000	0.03	Metal
CPZ	Level 1	6 million	71	Non-Metal
CPZ	Level 2	2 million	26	Non-Metal
CPZ	Level 3	0.2 million	2	Non-Metal
CPZ	Level 4	59,000	1	Non-Metal

Variable	Category	Area (km^2)	Percentage of area (%)	Mineral
MER	[0 - 1]	7 million	96	Non-Metal
MER	(1 - 2]	0.3 million	4	Non-Metal
ME	(2 - 3]	912	0.01	Non-Metal
MER	(3 - 4]	510	0.01	Non-Metal
CPZ	Level 1	7 million	63	Coal
CPZ	Level 2	4 million	35	Coal
CPZ	Level 3	0.2 million	2	Coal
CPZ	Level 4	51,000	0.5	Coal
MER	[0 - 1]	9 million	87	Coal
MER	(1 - 2]	1 million	13	Coal
MER	(2 - 3]	13,000	0.1	Coal
MER	(3 - 4]	34	0.0003	Coal

Supplementary Table 8: Contribution of different data sources to the total mining footprint. This table presents the percentage of mining footprint areas attributed to different combinations of data sources, including overlaps among Maus et al. (2022)[1], Tang et al. (2023)[2], and OpenStreetMap (OSM, 2024)[3]. Values represent the share of the global mining footprint that falls within each source or combination of sources. Because many polygons are captured by multiple datasets, the row values are not mutually exclusive. To calculate the total contribution of each dataset (e.g., Maus, Tang, OSM), one must sum all rows in which that dataset appears. These totals reflect the overall coverage from each source used in constructing the mining footprint dataset and correspond to the percentages cited in the main text.

**Data source**	**Percentage of mining footprint (%)**
Maus, 2022 & Tang, 2023 & OSM, 2024	38
Maus, 2022 & Tang, 2023	18
OSM, 2024	16
Maus, 2022	12
Maus, 2022 & OSM, 2024	8
Tang, 2023	6
Tang, 2023 & OSM, 2024	2

Supplementary Table 9: Data sources for mineral deposit locations. This table lists the data sources used to identify the locations of mineral deposits, categorised by mineral type (e.g., fuel, metals, non-metals and critical minerals). It includes references and details of each dataset utilized to compile a global distribution of mineral resources.

Data source	Fuel	Metal	Non-Metal	Critical minerals
SNL Metals & Mining[4]	✓	✓	✓	✓
WORLD Deposit Database for LEO[5]	✓	✓	✓	✓
Eikon[6]		✓		✓
Global Coal Mine Tracker[7]	✓			
Simon et al., 2023[8]	✓	✓		✓
Macklin et al., 2023[9]		✓		✓
USGS National Minerals Information Center[10]	✓	✓	✓	✓
Major mineral deposits of the world[11]	✓	✓	✓	✓
Mineral Resources Data System (MRDS)[12]	✓	✓	✓	✓
Dallaire-Fortier, 2024[13]	✓	✓	✓	✓

Data source	Fuel	Metal	Non-Metal	Critical minerals
Critical Minerals Mapping Initiative (CMMI)[14]		✓	✓	✓
USGS Deposits of critical minerals[15]		✓	✓	✓

Supplementary Table 10: Critical minerals for renewable and clean energy as defined by the International Energy Agency (IEA).

Name	Abbr.	Number of sites	Area (km^2)
Boron	B	10,433	38,000
Cadmium	Cd		
Chromium	Cr		
Copper	Cu		
Cobalt	Co		
Gallium	Ga		
Germanium	Ge		
Battery-grade graphite	C		
Hafnium	Hf		
Indium	In		
Iridium	Ir		
Lead	Pb		
Lithium	Li		
Magnesium	Mg		
Manganese	Mn		

Molybdenum	Mo		
Nickel	Ni		
Niobium	Nb		
PGMs (other than iridium)	PGE		
Selenium	Se		
Silicon	Si		
Silver	Ag		
Tantalum	Ta		
Tellurium	Te		
Tin	Sn		
Titanium	Ti		
Tungsten	W		
Vanadium	V		
Zinc	Zn		
Zirconium	Zr		
Arsenic	As		
Neodymium	Nd		

Dysprosium	Dy		
Praseodymium	Pr		
Terbium	Tb		
Yttrium	Y		
Lanthanum	La		
Total rare earth elements	REE		

Supplementary reference

1. Maus, V., Giljum, S., da Silva, D. M., Gutschlhofer, J., da Rosa, R. P., Luckeneder, S., ... & McCallum, I. (2022). An update on global mining land use. *Scientific data*, 9(1), 433.

2. Tang, L., & Werner, T. T. (2023). Global mining footprint mapped from high-resolution satellite imagery. *Communications Earth & Environment*, 4(1), 134.

3. OpenStreetMap Contributors. Openstreetmap wiki - organised editing activities. https://wiki.openstreetmap.org/wiki/Organised_Editing/Activities (2024). Accessed: 2024-03-30.

4. S&P. S&P Global Market Intelligence (S&P, New York, 2024).

5. WorldDeposit LEO: "*Proprietary dataset compiled by MinEx Consulting, dated late June 2022.*" (Global dataset of 7,059 moderate-sized deposits discovered since 1950)

6. Eikon database: "*Proprietary database compiled by LSEG's Metals research team and accessed through the Mine Production Screener and Physical Assets Screener of the Eikon Refinitiv platform on July 2024*" (LSEG stands for London Stock Exchange Group)

7. Global Coal Mine Tracker, Global Energy Monitor, September 2024 Supplement release.

8. Jasansky, S., Lieber, M., Giljum, S., & Maus, V. (2023). An open database on global coal and metal mine production. Scientific data, 10(1), 52.

9. Macklin, M. G., Thomas, C. J., Mudbhatkal, A., Brewer, P. A., Hudson-Edwards, K. A., Lewin, J., ... & Mangalaa, K. R. (2023). Impacts of metal mining on river systems: a global assessment. Science, 381(6664), 1345-1350.

10. USGS National Minerals Information Center. usgs.gov/centers/national-minerals-information-center

11. Major mineral deposits of the world. https://mrdata.usgs.gov/major-deposits/

12. Mineral Resources Data System (MRDS). https://mrdata.usgs.gov/mrds/

13. Dallaire-Fortier, C. (2024). A comprehensive historical and geolocalized database of

mining activities in Canada. Scientific Data, 11(1), 307.

14. Critical Minerals Mapping Initiative (CMMI). https://portal.ga.gov.au/persona/cmmi

15. Hammarstrom, J.M., Woodruff, L.G., and Dicken, C.L., 2023, Critical mineral deposits of the United States (ver. 2.0, April 2024): U.S. Geological Survey data release, https://www.usgs.gov/data/critical-mineral-deposits-united-states-ver-20-april-2024.
