# Supplementary material for: Post-2020 conservation priorities are threatened by global mining
Source: Nat Commun. 2026 Sep 21;17:11007. doi: 10.1038/s41467-026-77626-6 (PMC13687551; doi:10.1038/s41467-026-77626-6)
Supplement: Supplementary file 2 [file 41467_2026_77626_MOESM2_ESM.pdf]

nature portfolio

Corresponding author(s): Kaixuan Pan

Last updated by author(s): Aug 3, 2026

Reporting Summary

Nature Portfolio wishes to improve the reproducibility of the work that we publish. This form provides structure for consistency and transparency in reporting. For further information on Nature Portfolio policies, see our Editorial Policies and the Editorial Policy Checklist.

Statistics

For all statistical analyses, confirm that the following items are present in the figure legend, table legend, main text, or Methods section.

n/a	Confirmed	
☐	☒	The exact sample size (*n*) for each experimental group/condition, given as a discrete number and unit of measurement
☐	☒	A statement on whether measurements were taken from distinct samples or whether the same sample was measured repeatedly
☐	☒	The statistical test(s) used AND whether they are one- or two-sided<br>*Only common tests should be described solely by name; describe more complex techniques in the Methods section.*
☐	☒	A description of all covariates tested
☐	☒	A description of any assumptions or corrections, such as tests of normality and adjustment for multiple comparisons
☐	☒	A full description of the statistical parameters including central tendency (e.g. means) or other basic estimates (e.g. regression coefficient) AND variation (e.g. standard deviation) or associated estimates of uncertainty (e.g. confidence intervals)
☐	☒	For null hypothesis testing, the test statistic (e.g. *F*, *t*, *r*) with confidence intervals, effect sizes, degrees of freedom and *P* value noted<br>*Give P values as exact values whenever suitable.*
☒	☐	For Bayesian analysis, information on the choice of priors and Markov chain Monte Carlo settings
☒	☐	For hierarchical and complex designs, identification of the appropriate level for tests and full reporting of outcomes
☐	☒	Estimates of effect sizes (e.g. Cohen's *d*, Pearson's *r*), indicating how they were calculated

Our web collection on statistics for biologists contains articles on many of the points above.

Software and code

Policy information about availability of computer code

Data collection	No software or code is used to collect data in this study.

Data analysis

All spatial analyses were performed using the open-source software QGIS (version 3.40.3) and R (version 4.4.0), with RStudio (version 2023.12.1+402) as the integrated development environment. Spatial data processing and analysis in R were conducted using the sf package (version 1.0-16). Statistical analyses were performed in R using the agricolae package (version 1.3-7) and the emmeans package (version 1.8.8).

TThe code used to process the data, conduct the analyses, and generate the figures is available in Zenodo under accession code 10.5281/zenodo.21769493 (Pan et al. 2026) (Pan et al. 2026).

References:

-QGIS Development Team. QGIS Geographic Information System (2024).
-R Core Team (2024). R: A language and environment for statistical computing. R Foundation for Statistical Computing, Vienna, Austria. URL https://www.R-project.org/.
-Posit team (2023). RStudio: Integrated Development Environment for R. Posit Software, PBC, Boston, MA. URL http://www.posit.co/.
-Pebesma, E. Simple Features for R: Standardized Support for Spatial Vector Data. The R Journal 10, 439; 10.32614/RJ-2018-009 (2018).
-de Mendiburu F (2023). agricolae: Statistical Procedures for Agricultural Research. R package version 1.3-7, https://CRAN.R-project.org/package=agricolae.
-Lenth, R. emmeans: Estimated Marginal Means, aka Least-Squares Means_. R package version 1.8.5 (2023).
-Pan, K., Raes, N., Le Lann, W., Marshall, L., Feijoo Quezada, A., Barbarossa, V., Moens, M., Anderson, S., Kleijn, R.& Jacobus, C. B. (2026). Post-2020 conservation priorities are threatened by global mining. https://doi.org/10.5281/zenodo.21769493

For manuscripts utilizing custom algorithms or software that are central to the research but not yet described in published literature, software must be made available to editors and reviewers. We strongly encourage code deposition in a community repository (e.g. GitHub). See the Nature Portfolio guidelines for submitting code & software for further information.

Data

Policy information about availability of data

All manuscripts must include a data availability statement. This statement should provide the following information, where applicable:

- Accession codes, unique identifiers, or web links for publicly available datasets
- A description of any restrictions on data availability
- For clinical datasets or third party data, please ensure that the statement adheres to our policy

All data are provided in the Methods and Supplementary Information. Current mining footprint data were sourced from Maus et al. (2022)67, Tang et al. (2023)68, and OpenStreetMap (OSM, as of March 30, 2024)69. Data on future mining potential areas were obtained from Oakleaf et al. (2019)46. Biodiversity hotspots data were sourced from Hoffman et al. (2016)77; Last of the Wild data from Allan et al. (2017)78; and data on Alliance for Zero Extinction sites and Key Biodiversity Areas from BirdLife International79. Protected areas data were collected from the World Database on Protected Areas (WDPA)80,81, the China Nature Reserve Specimen Resource Sharing Platform (http://www.papc.cn/), and www.kursatozcan.com. Species range data were retrieved from the IUCN Red List and BirdLife International83,84. Endemic species data were gathered from WWF Wildfinder (https://www.worldwildlife.org/pages/wildfinder). Country income levels and management effectiveness were obtained from the World Bank (https://www.worldbank.org). Data on the distribution of deposits for various minerals are listed in Supplementary Table 9. The data generated in this study and required to reproduce all analyses and figures have been deposited in Zenodo under accession code 10.5281/zenodo.21769493.96

Research involving human participants, their data, or biological material

Policy information about studies with human participants or human data. See also policy information about sex, gender (identity/presentation), and sexual orientation and race, ethnicity and racism.

Reporting on sex and gender	not applicable
Reporting on race, ethnicity, or other socially relevant groupings	not applicable
Population characteristics	not applicable
Recruitment	not applicable
Ethics oversight	not applicable

Note that full information on the approval of the study protocol must also be provided in the manuscript.

Field-specific reporting

Please select the one below that is the best fit for your research. If you are not sure, read the appropriate sections before making your selection.

☐ Life sciences ☐ Behavioural & social sciences ☒ Ecological, evolutionary & environmental sciences

For a reference copy of the document with all sections, see nature.com/documents/nr-reporting-summary-flat.pdf

Ecological, evolutionary & environmental sciences study design

All studies must disclose on these points even when the disclosure is negative.

Study description	This study provides a global, spatially explicit assessment of the current and future threats of mining and quarrying activities to biodiversity. We quantify the overlap between mining footprints and Conservation Priority Zones (CPZs) and assess species extinction risks (MER) across 14 biomes. Additionally, we evaluate the potential future conflicts between mining expansion and biodiversity conservation using future mining potential areas.
Research sample	The study integrates multiple large-scale datasets covering: mining footprints across all continents.<br>Future mining potential areas from Oakleaf et al. (2019).
Sampling strategy	Mining footprint data were compiled from three major sources (Maus et al., 2022; Tang et al., 2023; OpenStreetMap, 2024). Using QGIS software, we processed these datasets to remove overlaps, creating a seamless global map of mining footprints.<br>Future mining potential areas were extracted from global-scale data (Oakleaf et al., 2019).
Data collection	Mining footprint data were derived from remote sensing databases and geospatial surveys (refs 5, 50, 68).<br>Future mining potential areas from Oakleaf et al. (2019) (ref 32).<br>Biodiversity data were obtained from global conservation databases (IUCN, BirdLife International, WDPA, WWF Wildfinder, China Nature Reserve Specimen Resource Sharing Platform, www.kursatozcan.com).<br>Data on the distribution of deposits for various minerals are listed in Supplementary Table 7.
Timing and spatial scale	Temporal scale: data collected up to March 2024.<br>Spatial scale: global
Data exclusions	No data were excluded from the analysis
Reproducibility	All of the results generated in this study are based on publicky-avaiable datasets and are reproducible.
Randomization	Not applicable. This study is based on global-scale spatial analyses using comprehensive datasets, not experimental or randomized sampling.
Blinding	Not applicable. This study relies on publicly available datasets and does not involve subjective measurements requiring blinding.

Did the study involve field work? ☐ Yes ☒ No

Reporting for specific materials, systems and methods

We require information from authors about some types of materials, experimental systems and methods used in many studies. Here, indicate whether each material, system or method listed is relevant to your study. If you are not sure if a list item applies to your research, read the appropriate section before selecting a response.

Materials & experimental systems

n/a	Involved in the study
☒	☐ Antibodies
☒	☐ Eukaryotic cell lines
☒	☐ Palaeontology and archaeology
☒	☐ Animals and other organisms
☒	☐ Clinical data
☒	☐ Dual use research of concern
☒	☐ Plants

Methods

n/a	Involved in the study
☒	☐ ChIP-seq
☒	☐ Flow cytometry
☒	☐ MRI-based neuroimaging

Plants

Seed stocks	Not applicable
Novel plant genotypes	Not applicable
Authentication	Not applicable
