# Supplementary material for: Post-2020 conservation priorities are threatened by global mining
Source: Nat Commun. 2026 Sep 21;17:11007. doi: 10.1038/s41467-026-77626-6 (PMC13687551; doi:10.1038/s41467-026-77626-6)
Supplement: Supplementary file 3 [file 41467_2026_77626_MOESM3_ESM.pdf]

nature portfolio

Peer Review File

Post-2020 conservation priorities are threatened by global mining

Corresponding Author: Dr Kaixuan Pan

This manuscript has been previously reviewed at another journal. This document only contains information relating to versions considered at Nature Communications.

This file contains all reviewer reports in order by version, followed by all author rebuttals in order by version.

Version 0:

Reviewer comments:

Reviewer #1

(Remarks to the Author)
Understanding the impacts of mining on biodiversity is an important question. The paper uses a comprehensive collation of mining footprint data and overlaps those mine footprints with conservation priority zones. The authors also calculate a mean extinction risk score for vertebrate species that have mining as a threat within their IUCN Red List species assessment. Within the manuscript, the authors also compare the proportions of 14 biomes that overlap with mine footprints. They also overlap areas of potential expansion of the metals, non-metals and coal with CPZs.

General comment: Please use line numbers on your submitted manuscript to aid Reviewer's

Major comments

(1) The manuscript lacks clarity in presenting the results of the paper. There are lots of spatial overlaps between mining footprints and different conservation areas but is hard to follow in places and could generate confusion. The language describing the results likely needs to change substantially within the manuscript.

For examples: P6 Paragraph 2 "The highest pressure on CPZs occurs in Southeast Asia (e.g. Indonesia, Malaysia and Philippines)". What is meant by the highest pressure in this result? Does this mean the largest area of overlap of mine footprints with CPZ or area mines are overlapping with highest level CPZ, or is it both?

"Under the scenario where these impacts spread just 1 km beyond the footprint, the range of threats to CPZs and MER could increase by 4–8 times and 7–66 times, respectively, assuming total habitat destruction with no remaining ecological functionality (Supplementary Table 4). If the impact radius expands to 5 km, the effects could escalate to 27–54 times and 53–562 times the current mining footprint's threats (Supplementary Table 4). This underscores that strictly containing mining impacts within designated areas can significantly reduce potential threats to biodiversity." Again, it is hard to know what this means from just the main text. Does 'effects' mean the area of CPZ's that overlap with mining area/buffers or is it related to the level of CPZ's and MER?

"For mining footprints inside protected areas, the highest threats to CPZs is observed in Tropical & Subtropical Moist Broadleaf Forests (11.4%), with 3.2% in zero extinction zones (Fig. 2b)." This is confusing in its wording, which makes it difficult to understand what the percentages are referring to. We assume they are referring to the percentages of the mine footprint that are within PAs and within Trop Moist Broadleaf that overlap with CPZs. It would also be good to know the actual of mine footprint that these numbers represent as you mention mines within PAs do not contribute much in terms of total footprint as shown in the supplementary information.

(2) The statistical analysis is poorly explained and we suspect is not sufficiently robust (leading into Figs 2de). The authors are listing habitats with highest MER within and outside PA ("Mining's threat to species extinction, as measured by MER, is highest within protected areas in…AND "Outside protected areas, the highest MER is observed in….). This is encouraging readers to make an implicit comparison between the two spatial locations of mines (in / out of PA) and impacts on biomes –

one that cannot be made using the analysis conducted -- and it fails to really get at the important question of whether mining inside PAs is driving more outcomes in certain biomes than is mining outside of PAs. The authors need to directly contrast within and outside PAs within one model, probably accounting for socio-economic and biophysical variables. There is phylogenetic non-independence in these species data that needs to be taken account of.

More broadly, there are other areas where it would be prudent to do some analysis. E.g. are mines really more over-represented in one region than another? The authors use % to make this point, but we'd like to know whether certain biomes are statistically more at risk than others.

(3) We recommend including a visual methods to help explain and visualise the overlaps of data the authors use these overlaps in combination.

Minor comments
Abstract. (4) Avoid use of 'However'

(5) "threatening over 2,300 species, including many classified as threatened". States threatening for 2300 species, but if some of these species are not classified threatened, then we are not convinced that the authors can claim that mining is threatening those species (perhaps some benefit from mining, perhaps some are not impacted). Surely these are overlapping with?

(6) Please reword: "These threats span 14 biomes, with the greatest risks in fragile ecosystems such as Mangroves and Mediterranean." These biomes are disproportionately affected but greatest risk is likely within biomes with more overall mining such as Tropical moist broadleaf forest and Desert and Xeric shrubland.

Introduction
(7) The final paragraph would benefit from clearly outlining study objectives. There is more on the methods and some conclusive points (that lack specificity) than the actual questions

Results
(8) P7 Mangroves and the Mediterranean biome show the highest and 3rd highest percentages of extraction, respectively (Fig. 2a, Supplementary Fig. 5). "Percentage of extraction" makes it sound like they are the 1st and 3rd largest producers not that they are the highest in terms of relative proportion of extraction. Might be good to highlight any studies that focus on mining impacts from mining in mangroves, or the lack of studies if that's the case.

(9) The result in Figure 3b where the majority of overlap of future non-metal mineral deposits and CPZ and MER in North America is somewhat surprising, as these minerals are extracted globally in lots of highly biodiverse regions. Perhaps this is due to some bias within the Oakleaf 2019 dataset, as the maps are proxies created from density of mapped deposit? It would not be surprising that North America has more detailed maps of deposits than many regions. Either way, as the authors recommend these regions to be excluded from future development it is important to further explain this result and address this possibility.

Discussion
(10) Lamb et al. use IUCN threat assessments to derive hotspots of risk from mining. How do the findings in your study match to those? Might also be worth mentioning this study in the Introduction given that both use IUCN as a method of assessing risk, with this study making an advance by using mine polygons.

Figures
(11) Would recommend changing the colour scheme in the Figures isn't particularly colour blind friendly. Might benefit from reducing the line width or removing country borders, so that the detail of the small pixels is easier to see.

Methods
(12) What about species ranges that are passage or introduced etc., were these species included in the analysis? "Species range data were obtained from the IUCN Red List and BirdLife International61,66. We focused on species threatened by mining activities, excluding species classified as Data Deficient (DD) or Extinct (EX, EW). The final dataset includes 3,094 species across five taxa: 754 amphibians, 751 plants, 697 reptiles, 463 mammals, and 429 birds. This dataset was used to assess species distributions that overlap with mining footprints and to evaluate species currently covered and not covered by protected areas."

(13) "Each mining polygon was assigned a value based on CPZ levels and mean extinction risks (MER) of imperilled species. MER was calculated by averaging assigned values from IUCN Red List categories within each polygon:" Unclear, do the authors mean assigned the value of CPZ that intersects with the mining footprint?

(14) A reference for the method of assigning commodities to mines by using underlying deposit maps would be useful. There is also no comment on the potential errors that may occur from this.

Reviewer #2

(Remarks to the Author)
I co-reviewed this manuscript with one of the reviewers who provided the listed reports. This is part of the Nature Communications initiative to facilitate training in peer review and to provide appropriate recognition for Early Career Researchers who co-review manuscripts.

Reviewer #3

(Remarks to the Author)
Review NCOMMS-25-18071-T

Summary and General comments:

This paper examines current and future mining threats to biodiversity "hotspots" on a global scale using a spatially explicit approach. The authors achieve this by integrating various mining footprint datasets with mineral deposit data and conducting overlay analyses alongside biodiversity datasets.

The resulting biome-specific assessments of potential future conflicts between mining and biodiversity are highly relevant to the conservation and sustainable mining communities, as well as to global environmental protection stakeholders and activists. To the best of my knowledge, this is the first study to comprehensively compile a spatially explicit, global-scale dataset linking mining threats and biodiversity hotspots. The authors have utilized the most currently available datasets, and I did not identify any significant omissions in terms of mining footprints, mineral deposits, or biodiversity hotspots.

There is one other recent similar study that is probably coarser and not as comprehensive as this one but should be included in this paper.

Reference: Lamb, Ieuan P. et al. Global threats of extractive industries to vertebrate biodiversity. Current Biology, Volume 34, Issue 16, 3673 - 3684.e4. DOI: 10.1016/j.cub.2024.06.077.

Thus, I recommend highlighting more the novelty and added value of this particular study compared to other recent studies (e.g. by Lamb et al. (2024), Sonter et al.2018, 2020, 2022) having focused on the same topic.

The weakest part of this paper is the final section on "challenges and solutions" for achieving biodiversity-inclusive mining. While the authors' call for action is justified (i.e. halt mining in biodiversity hotspots; coordinated global actions to reconcile resource extraction with biodiversity conservation), the proposed solutions remain broad and lack specificity. For example, strengthening governance frameworks and addressing illegal mining activities are mentioned to be of high priority. Advanced technologies and nature-based solutions are mentioned to be effective impact mitigation instruments. The recommendations are very general which limits the section's added value. Questions to be addressed are : What kind of actions have been effective in the recent past? Provide concrete examples supported by references. By whom do they need to be implemented and accepted in order to be successful? For example, in the recent past local communities often were left out in so called "biodiversity offsetting" projects implemented by mining companies, which may lead to new conflicts of interest between conservation and local communities, besides the original one between mining and conservation. To strengthen this part of the paper, the authors should include concrete, evidence-based examples, where mining, biodiversity conservation, and both national and local interests successfully align to enhance the substance and credibility of this section.

Particularly, the role of local communities in integrated, sustainable land and biodiversity management is notably absent and should be addressed to provide a more comprehensive and inclusive perspective.

Methodologically I did not notice any flaws. Some method. details are missing here and there (see detailed comments) and I miss a sensitivity analysis (or other justification, e.g. strong references) to support the two buffers distances that were chosen. No flaws were found in the interpretation of the results.

The paper is well written and structured.

Detailed comments:

Note: Continuous line numbering was applied to the main manuscript to point out specific lines. Further, citations of manuscript text in this review is formatted in Times New Roman font, suggestions made by the reviewer are formatted in Aptos font.

Line 45:"…China, Brazil and Indonesia are mentioned as particularly affected." I miss a "most affected country (or countries) on the African continent, which also envisage economic growth but have weak social and environmental governance structures.
Line 57: "To date, no spatially explicit, global-scale analysis has been conducted, hindering the development of effective conservation strategies at both regional and global scales." Please consider mentioning Sonter et al. (2022) and Lamb et al. (2024) who performed similar studies (although maybe less comprehensive) and concretize the added value and novelty of your study. (see my general comment above).
Line 62: "Future mining is heavily influenced by socio-economic, geopolitical and technological trends, such as the global push for renewable energy and infrastructure development, which drive demand for specific materials like energy resources,

metals, and non-metals. To address the diverse impacts of these materials on biodiversity, material-specific analyses are needed. This will enable…". How? It is unclear how "specific analyses" will help developing effective strategies. Please be more specific.
Line 78-81: "Ultimately, our findings can guide conservation investments by identifying and evaluating where and how to manage trade-offs between mining activities and biodiversity conservation, while mitigating future pressures." I do agree that this study provides new results on the "where" mining threats to biodiversity are highest but not on the "how". The current version of this paper does not provide new promising recommendations on how conservation of biodiversity could become more effective. In my view this is one of the main weaknesses of this paper.
Line 90: "…Southern Hemisphere. One-third of these mining areas…" It is unclear whether "one-third" refers to the previous sentence (i.e. Southern Hemisphere) or not. Please clarify in the text.
Line 99: "…beyond their immediate footprint…such as illegal hunting and deforestation." This is indeed a very important point. Off-site as well as indirect negative effects on biodiversity, such as air, water and soil contamination/pollution should be emphasized more as they are often neglected as well as underestimated. The authors mention other effects later on line 242, page 11, which I think is important and required. Maybe this could be highlighted a bit more.
Line 101ff: To account for offsite impacts ("impact spread") the authors use buffers of 1km and 5km. Please provide statistical evidence that supports your choice of buffer distances (e.g. perform a sensitivity analysis with varying buffer distances and provide these results in the supplementary material), or, in addition to this, provide references that support the choice of these buffer distances. Other studies focussing on off-site impacts have found effects on vegetation (Liu et al. 2022; Ecological Indicators) and water quality/river sediments (Lin et al. (2022; Environmental Research) and deforestation (Giljum et al. 2022) farther away than 5km from mining sites. Note: these studies are just a non-representative, random selection.
Line 111ff: "Most mining occurs outside protected areas (Supplementary Fig. 2a,b), with a small percentage taking place within CPZs (Supplementary Fig. 3a). However, within protected areas, over 60% of mining footprints threaten CPZs, including 11% in zero-extinction zones (CPZ level 4) (Supplementary Fig. 3a)." Do I understand correctly, that within PAs 60% of mining footprints overlaps with CPZ and 11% of those 60% are within CPZ level 4? Not easy to grasp and understand this sentence. Also, I am wondering whether it would make sense to provide absolute numbers in brackets? Absolute numbers are provided in the Abstract and in the "future threats" section but not the "global distribution" section. Please check and adjust accordingly to be consistent throughout the entire manuscript.
Line 133 and Line 146: 0.96% and 9.5% and other percentages provided here. Please consider providing the absolute area number as well.
Line 151: (>=50%) Here, as it is written now, it is a bit unclear what the 50% are referring to. Please consider rewriting this sentence to improve clarity.
Line 185: "primarily located in Russia, Indonesia, China, Australia, Southern Africa, and parts of the Americas". Can you name most affected countries also for "the Americas", which is a very large area compared to the others that you mention to keep a similar level of spatial detail in the description of the most affected countries/regions.
Line 191: "Without proactive planning and management, mining expansion could jeopardize conservation initiatives and undermine global biodiversity objectives." The results of this study justify a stronger wording here. I would argue that mining expansion not only undermines global biodiversity objectives but could lead to increased biodiversity loss.
Line 225 - 239: This entire paragraph as part of the section on "Achieving biodiversity-inclusive mining, challenges and solutions" remains rather general. It requires improvement to be of added value. Please see my previous comment and also under "General comments". Furthermore, I elaborated some my points here once again. I hope it helps the authors and apologize for any repetition.
The authors call for a halt of mining activities in biodiversity hotspot which sounds great on paper but they do not provide pathways of how this could finally realized. The recommendations they make remain general and are well known (e.g. "strengthening governance frameworks, …addressing illegal mining activities in conservation-priority areas…"). The question is: how can frameworks become more effective?
Another example: "By embedding biodiversity safeguards throughout the lifecycle of mining projects, it is possible to align resource extraction with conservation priorities, fostering a sustainable future that supports both nature and human well-being." Can you be more concrete and provide references of success stories/examples (from the global North and South) that show that resource extraction can go together with effective conservation measures. More concretely I miss important aspects: engaging of local stakeholders, true involvement of local communities towards more inclusive, effective and meaningful biodiversity offsetting programs. Regulations require financial and human resources to be implemented and enforced. Thus, mining companies should have to engage more in this.

Line 251: "..urgent need for a coordinated global framework to systematically monitor all mining and quarrying activities." Well, what exactly do you mean with that? I don't think this will address biodiversity conservation effectively. There exist already several global instruments, i.e forums and standards with the goal to ensure sustainable mining (IGF, EITI, IRMA Standard, etc.). I don't think another one is required.
Line 253ff:The last paragraph sounds similarly superficial and is not convincing.

Figures:
Figure 2 caption: Please format d, e (bold).
Figure 3: I suggest adding a figure d) showing the same map for critical minerals in addition to the a) metal, b) non-metal, and c) coal maps. While you mention this category in Supplementary Figure 9 and in the text you do not provide a map for this category. I believe this would be of high interest to the readers of this paper. (see also my comment in the methods part on a potential consistency issue.)

Methods:
Line 441, Line 442: Here you mention in brackets that 76% of the total footprint area is covered by Maus et al.'s dataset. Similarly, Tang et al., and OSM cover 64%, respectively. However, in Supplementary Table 6 these numbers are different

(12%, 6% and 16%, respectively). Please clarify, harmonize or correct, either in the table or in the methods part.
Line 446: “By complementing satellite imagery datasets such as those from Maus et al. (2022)49 and Tang et al. (2023)50, OSM enhances…” The datasets are not satellite imagery but are footprints derived from satellite imagery. Please correct the sentence to “By complementing mining footprint datasets derived from satellite imagery”.
Line 448: It seems that you just added up the two binary datasets using a boolean OR. If this is not the case, please describe the overlay functions that you used more specifically. Also, please mention the date/year of each dataset. It is important to know what mining footprint year they represent. (Illegal) mining processes are very fast land change processes. The used datasets might already be outdated by now and underestimate footprints by the time this paper is published.
Line 469: You write that you joined “deposit type” with your merged mining footprint dataset. For the deposits you used twelve (Supplementary Table 7) different datasets. I have two method. questions regarding the deposit assignment: a) How did assign the deposit type in case of multiple minerals and/or non-minerals present at one location, and b) how did you assign it, in cases of different datasets listing different minerals, i.e. disagreeing in the deposit minerals?
Line 476: What is the percentage of “unknown” deposit type per mining footprint of the total number (or area) of mining footprints?
Line 506: “A gap analysis was conducted to identify CPZs that are covered and not covered by existing protected areas.” Unclear formulation, consider rephrasing.
Line 535: Besides covering socio-economic factor(s) I suggest to cover also governance factors. Thus, in addition to income, I would add one of the six WorldBank governance indicators (https://www.worldbank.org/en/publication/worldwide-governance-indicators).
Line 547: Please add the abbreviation definitions for LC, NT, VU, EN and CR species.
Line 557: see my previous comment on the justification of your choice of buffer distances. Besides strengthening it with references I suggest you perform a sensitivity analysis where you perform your calculations for a range of distances, e.g. 0 – 30 km with increment of 5km, or similar. The results can be presented as part of Supplementary Table 4.
Line 571: “ten equal categories based on quartiles”. This categorization is useful when datasets are NOT skewed. This is the case for the global mining footprint (visible in Supplementary Figure 1) but how about the CPZs and MER?

Supplementary Figure 8 caption: There is currently the same caption text (i.e. copy/paste) for panels a, b and c. Please correct.

Supplementary Figure 9: Here you show results for the additional category “critical minerals”, which is interesting. Is it possible to include this category for some of your main results/figures as well? If so, please add (see one of my previous comments on Figure 3). Similarly, Supplementary Table 1: Is it possible to add the category “critical minerals” here as well? Critical minerals are of high interest to readers and it would increase consistency throughout your entire study results.

Version 1:

Reviewer comments:

Reviewer #1

(Remarks to the Author)
The authors have made several changes to the manuscript in dealing with the comments, including some new analysis and several tweaks to the text to improve clarity. However, I am left with a substantial concern about the novelty of the work – the revisions if anything have not helped here and I feel the opportunity to really identify the novelty and how this work differs with others has been missed. I also feel that the authors had the opportunity to go further in their analysis to provide real inference (as was requested in the first review). Lastly, I am not keen on having three Figures, all maps. This limits reader’s ability to extract meaningful patterns and also limits the study’s novelty by preventing explanation of why there are particular findings made.

(1) With respect to the novelty of this study vs Lamb et al. This was a concern in the first review and was also highlighted also by Ref 3. Unfortunately, the authors response to these issues has highlighted less novelty in their study relative to Lamb et al, not more.

First, there is a degree of conceptual inconsistency in the new text in the Introduction. Lamb et al used IUCN threat listings to map hotspots or mining risk, and the authors now state “but these approaches are typically based on point data3 or expert-derived threat data applied at coarse spatial resolutions18, limiting their ability to inform targeted conservation action for specific sites”. The first part is true, but I would disagree with the second part. More importantly, the authors’ statement undermines the utility of their own analysis, which also applied the use of the same IUCN range maps, threat assessment, and threat score to generate “conservation priority zones and species-level extinction risk metrics”. They cannot have it both ways and suggest that these are poor data for assessing mining impacts, but then promote them as being rigorous for their own analysis.

In fact, CPZ integrates four datasets, at least one of which (Key Biodiversity Areas-KBAs) is entirely derived from IUCN range mapping and threat assessments. How the other three datasets generate understanding of where species have range is also an important question (ie did they use the IUCN to do so? I suspect so in e.g. AZE). Similarly, the species-level extinction risk metrics are entirely derived from using data IUCN too. Therefore, there is much similarity with the Lamb et al. study, with the only substantial difference being that in this paper the authors have used precise mine locations against which to map their largely IUCN-derived metrics.

It would have been a lot more helpful for readers to know why mapping precise locations was more informative and then how findings from this study differed (if at all) from the core hotspots of mining risk revealed in Lamb et al. That would go a lot further in convincing of novelty than saying that Lamb et al. used “expert-derived threat data applied at coarse spatial resolutions18” [Ref 18 is Lamb et al.] but then doing so themselves.

(2) Absent of this comparison, I have contrasted the mean extinction risk (MER) map (Fig 1b) with those in Lamb et al, and there are a few key differences, especially in that this study only highlighted a few tiny points in the Americas as being of high MER (ie blue to pink). I have to ask why this has occurred, because it is quite a strange finding and one that does not match with Fig 1a either.

I think it could be through the use of the Meng et al. scoring approach to LC to CR. They use 0 to 4 scale whereas other authors (eg Strona et al. 2018 PNAS; Wang et al. 2020 Curr Biol) used scales that upweight very rare species relative to commoner ones by a far larger degree. Anyway, this needs exploration for me to have confidence in the findings.

(3) Re. contrasting the percentage of mines inside and outside PAs, this has not actually used any formal statistical interrogation but this was something highlighted in the first review as being important. Rather than offering statements of weak confidence i.e. “When comparing mining inside and outside protected areas within the same biome, the proportional overlap of mining footprints with CPZs is generally higher inside protected areas, although similar in Mangroves and Tropical & Subtropical Coniferous Forests”, the authors should have conducted an analysis and then been explicit in saying that mines in X Y Z biomes were located significantly more inside than outside PAs.

(4) The requested analysis from the first review identifying socio-environmental predictors reveals interesting findings, but these are not mentioned in the main text, just pointed to in a supplementary table. What readers should glean about these patterns should have been spelled out and potentially accompanied by Figures.

(5) Figure 2 – why use an image of a golden-winged warbler everywhere. First it is not an endemic and second it doesn’t live globally either. At least an endemic species could be pictured.

Reviewer #2

(Remarks to the Author)
I co-reviewed this manuscript with one of the reviewers who provided the listed reports. This is part of the Nature Communications initiative to facilitate training in peer review and to provide appropriate recognition for Early Career Researchers who co-review manuscripts.

Reviewer #3

(Remarks to the Author)
Reply to Authors Revised Manuscript (NCOMMS-25-18071A)

Dear Editor, dear Authors,

I would like to thank the authors for the effort they invested in revising the manuscript to further enhance its clarity and robustness. Whenever feasible, they have adequately addressed my suggestions.

Please find below one remaining minor request and a few more specific replies to the reviewers:

Reviewer 3:
Comment 30 (Pages 14–15 in the rebuttal document):
The authors addressed this comment by performing a sensitivity analysis and adding the results in Supplementary Table 4. The sensitivity analysis is described in the Methods section but it does not yet explicitly refer to Supplementary Table 4. I suggest adding “(Supplementary Table 4)”. It will help readers easily locate the corresponding sensitivity analysis results in the supplementary materials.

Comment 21:
I would like to thank the authors for considering my suggestion and assessing the feasibility of developing a prospectivity map for critical minerals. It is unfortunate that the necessary data are not available from Oakleaf et al. (2019).
It may be worthwhile to highlight this data gap or limitation more explicitly in the Methods section (near lines 669–682 of the revised manuscript).
I also thank the authors for adding the available information on critical minerals where possible (Supplementary Table 1), i.e. adding this information on current critical mineral mining footprints.

Version 2:

Reviewer comments:

Reviewer #1

(Remarks to the Author)
The authors have engaged with my request for a formal analysis to back up their patterns and have strengthened their presentation of several other findings. I have a one remaining issue, which can be simply solved by deleting some text, and a couple of minor edits.

The 5 km buffer analysis of “total habitat destruction” as a multiplicative of mine footprint is quite extreme but makes an important point (“Under the scenario where these impacts spread just 5 km beyond the footprint, the area of overlap with various levels of CPZs and MER could increase by 28-55 times and 53-566 times, respectively, assuming total habitat destruction with no remaining ecological functionality”). However, “If the impact radius expands to 30 km, the affected area overlapping with different levels of CPZs and MER could escalate to 240-324 times and 269-751 times the current mining footprint’s threats (Supplementary Table 4)” moves towards hyperbole and I suggest deleting it – I cannot think of a single mine that has destroyed ecological function in a 30 km radius.

L51 paragraph is very long, split.

L218-219, join as a single paragraph.

Figure 1: there is no need for the panels C and D to be so deep, there is just a lot of wasted whitespace.

Open Access This Peer Review File is licensed under a Creative Commons Attribution 4.0 International License, which permits use, sharing, adaptation, distribution and reproduction in any medium or format, as long as you give appropriate credit to the original author(s) and the source, provide a link to the Creative Commons license, and indicate if changes were made.
In cases where reviewers are anonymous, credit should be given to 'Anonymous Referee' and the source.
The images or other third party material in this Peer Review File are included in the article’s Creative Commons license, unless indicated otherwise in a credit line to the material. If material is not included in the article’s Creative Commons license and your intended use is not permitted by statutory regulation or exceeds the permitted use, you will need to obtain permission directly from the copyright holder.
To view a copy of this license, visit https://creativecommons.org/licenses/by/4.0/

Response to the reviewers' comments:

General remarks:

We sincerely thank the reviewers for their constructive and insightful comments. Their feedback has significantly improved the clarity and overall quality of the manuscript. We have carefully revised the manuscript in response to all comments. All modifications are highlighted in **yellow** in the revised manuscript. In this response letter, we provide point-by-point replies to each comment (reviewer comments in **black**, our responses in **blue**), and where relevant, we include the revised text from the manuscript (shown in **blue italics**). We hope these revisions satisfactorily address all concerns raised.

REVIEWER COMMENTS

Reviewer #1 (Remarks to the Author):

Understanding the impacts of mining on biodiversity is an important question. The paper uses a comprehensive collation of mining footprint data and overlaps those mine footprints with conservation priority zones. The authors also calculate a mean extinction risk score for vertebrate species that have mining as a threat within their IUCN Red List species assessment. Within the manuscript, the authors also compare the proportions of 14 biomes that overlap with mine footprints. They also overlap areas of potential expansion of the metals, non-metals and coal with CPZs.

General comment: Please use line numbers on your submitted manuscript to aid Reviewer's

Response**:** Thank you for pointing this out. We have added line numbers in the revised manuscript to facilitate the review process.

Comment(1) The manuscript lacks clarity in presenting the results of the paper. There are lots of spatial overlaps between mining footprints and different conservation areas but is hard to follow in places and could generate confusion. The language describing the results likely needs to change substantially within the manuscript.

Response**:** We appreciate this feedback and have revised the results section throughout for clarity and precision. Specifically, we now define more clearly what is meant by terms such as "highest pressure" and "effects" in each relevant sentence, and we clarify whether percentages refer to the total mining footprint overlapping with CPZs or specific CPZ levels. Detailed responses to the reviewer's examples are provided below.

For example: P6 Paragraph 2 "The highest pressure on CPZs occurs in Southeast Asia (e.g. Indonesia, Malaysia and Philippines)". What is meant by the highest pressure in this result? Does this mean the largest area of overlap of mine footprints with CPZ or area mines are overlapping with highest level CPZ, or is it both?

Response**:** In the revised manuscript, we have clarified that by "the highest pressure on CPZs," we refer specifically to the overlap between mining footprints and high-level CPZs.

The revised sentence now reads (modifications highlighted in yellow): "*The highest pressure on CPZs, defined as the overlap of mining activities with the highest-level conservation priority zones, occurs in Southeast Asia (e.g. Indonesia, Malaysia and Philippines), Australia, New Zealand, Middle East, Southern Europe, North and West Africa, and parts of the Americas (e.g. Brazil, Chile, Colombia, Mexico, and the eastern and western United States) (Fig. 1a).*" (pages 6-7, lines 123-128).

"Under the scenario where these impacts spread just 1 km beyond the footprint, the range of threats to CPZs and MER could increase by 4-8 times and 7-66 times, respectively, assuming total habitat destruction with no remaining ecological functionality (Supplementary Table 4). If the impact radius expands to 5 km, the effects could escalate to 27-54 times and 53-562 times the current mining footprint's threats (Supplementary Table 4). This underscores that strictly containing mining impacts within designated areas can significantly reduce potential threats to biodiversity." Again, it is hard to know what this means from

just the main text. Does ‘effects’ mean the area of CPZ’s that overlap with mining area/buffers or is it related to the level of CPZ’s and MER?

Response**:** Here the ‘effects’ refer to ‘area overlapping with different levels of CPZ and MER’. We have revised the relevant sentence to improve clarity and avoid confusion.

Now the sentence reads: “*Under the scenario where these impacts spread just 5 km beyond the footprint, the area of overlap with various levels of CPZs and MER could increase by 28-55 times and 53-566 times, respectively, assuming total habitat destruction with no remaining ecological functionality (Supplementary Table 4). If the impact radius expands to 30 km, the affected area overlapping with different levels of CPZs and MER could escalate to 240-324 times and 269-751 times the current mining footprint’s threats (Supplementary Table 4).*” (page 6, lines 114-120).

“For mining footprints inside protected areas, the highest threats to CPZs is observed in Tropical & Subtropical Moist Broadleaf Forests (11.4%), with 3.2% in zero extinction zones (Fig. 2b).” This is confusing in its wording, which makes it difficult to understand what the percentages are referring to. We assume they are referring to the percentages of the mine footprint that are within PAs and within Trop Moist Broadleaf that overlap with CPZs. It would also be good to know the actual of mine footprint that these numbers represent as you mention mines within PAs do not contribute much in terms of total footprint as shown in the supplementary information.

Response**:** Yes, in this context, the term “percentages” refers to “the proportion of mining footprints located within protected areas and, within each biome, overlapping with Conservation Priority Zones (CPZs).” However, to align with the reviewer’s question in **Comment (2) below**, specifically, “whether mining within protected areas (PAs) results in higher extinction risks than outside”, we conducted a new analysis to assess whether mining within PAs shows a disproportionately higher overlap with CPZs compared to mining outside PAs. The paragraph has been revised accordingly and now reads:

“*When comparing mining inside and outside protected areas within the same biome, the proportional overlap of mining footprints with CPZs is generally higher inside protected areas, although similar in Mangroves and Tropical & Subtropical Coniferous Forests (Supplementary Figure 6).*” (page 9, lines 179-182).

Comment(2) The statistical analysis is poorly explained and we suspect is not sufficiently robust (leading into Figs 2de). The authors are listing habitats with highest MER within and outside PA (“Mining’s threat to species extinction, as measured by MER, is highest within protected areas in…AND “Outside protected areas, the highest MER is observed in….). This is encouraging readers to make an **i**mplicit comparison between the two spatial locations of mines (in / out of PA) and impacts on biomes - one that cannot be made using the analysis conducted -- and it fails to really get at the important question of whether mining inside PAs is driving more outcomes in certain biomes than is mining outside of PAs. The authors need to directly contrast within and outside PAs within one model, probably accounting for socio-economic and biophysical variables. There is phylogenetic non-independence in these species data that needs to be taken account of.

Response**:** We thank the reviewer for this insightful comment. Several concerns are raised, which we address in detail below.

1. “The statistical analysis is poorly explained…”

Response**:** We have revised this paragraph to provide a clearer explanation. In addition, we clarify that the three key questions (as also suggested in **Comment (7)**) in this study are : “*(1) To what extent do current*

mining footprints overlap with international conservation priority zones (CPZs) and areas of elevated species mean extinction risk (MER)? (2) How do current mining-related impacts on CPZ and MER vary across biomes? (3) Where are the most at-risk regions from projected future exploitable mining areas?"(page 5, lines 84-87).

The objective of the corresponding section ("Biome-specific threats"), from which this comment originates, is to identify how extinction risk from mining differs among biomes and which biomes currently exhibit the highest MER, thereby informing decision-makers on which biomes should be prioritized for biodiversity conservation and impact mitigation efforts.

To address this, we performed a formal test (Kruskal-Wallis tests**)** followed by post hoc Dunn's tests to assess significant differences in MER across 14 biomes. This non-parametric approach is appropriate for comparing independent groups and is widely applied in ecological analyses. The results (Page 28, Fig.2) report degrees of freedom and *p*-values according to *Nature Communications* standards, and letters above each boxplot indicate statistically significant pairwise differences among biomes. We have simplified the analysis and revised Fig. 2 and the corresponding main text (page 9, lines 183-190) to better address the question, *"How do current mining-related impacts on CPZ and MER vary across biomes??"*, presenting a clearer and more straightforward interpretation.

Now the corresponding paragraph reads: "*The threat of species extinction from mining, as measured by MER, is highest in Tropical & Subtropical Grasslands, Tropical & Subtropical Dry Broadleaf Forests, Tropical & Subtropical Moist Broadleaf Forests, Mangroves, and Flooded Grasslands & Savannas (Fig. 2c). Furthermore, when comparing mining inside and outside protected areas within the same biome, MER is generally significantly higher inside protected areas (Supplementary Table 6). This finding underscores that even areas outside protected boundaries require stringent regulation, as many unprotected landscapes still function as critical habitats for threatened species[26].*"

2. On comparing inside vs. outside protected areas in one model

Response**:** We appreciate the reviewer's suggestion to explore whether mining within protected areas (PAs) results in higher extinction risks than outside, and to include socio-economic or biophysical variables in the analysis. In response, we conducted an additional generalized linear model (GLM) incorporating protection status, biome, and socio-economic variables to explore factors associated with biodiversity risk from mining. As biome categories already reflect major underlying biophysical gradients (Olson et al., 2001), no additional biophysical variables were included. The results of this exploratory model are now presented in the revised Methods (pages 41-42, lines 827-836) and Supplementary Table 6. Furthermore, we have added a statement in the main text. (page 9, lines 186-190).

3. On phylogenetic non-independence

Response**:** We acknowledge the reviewer's concern regarding potential phylogenetic non-independence among species. However, this issue is most relevant for species-level analyses. In our case, the analysis unit is the mining site, not individual species. MER is an aggregate metric, representing the mean IUCN threat score (ranging from LC = 0 to CR = 4) for all species overlapping a given mining footprint, in line with previous study (Meng et al., 2023). As such, we do not model individual species' extinction probabilities or test species-level predictors.

Furthermore, because MER averages threat status across multiple species spanning diverse taxa, any phylogenetic signal is effectively diluted and does not bias our results. Our objective is to quantify the

current distribution of extinction risk associated with mining, not to infer evolutionary or trait-based causality.

More broadly, there are other areas where it would be prudent to do some analysis. E.g. are mines really more over-represented in one region than another? The authors use % to make this point, but we'd like to know whether certain biomes are statistically more at risk than others.

Response**:** We thank the reviewer for the suggestion regarding statistical analysis. However, we believe formal tests are not appropriate here. As the reviewer notes, the relevant question is "are mines really more over-represented in one region than another?". This can be addressed directly by comparing the proportion of each biome's area affected by mining.

In addition, since each biome's dataset consists of a single aggregated value (total mining footprint area), there is no within-biome variation, making statistical significance testing unfeasible.

Instead, we use the percentage of biome area affected by mining to assess relative representation, a widely accepted approach in spatial and conservation analyses. For example, UNEP-WCMC and IUCN (2024) and TNFD (2023) use similar percentage-based metrics to evaluate ecological impacts. Recent studies (e.g., Aska et al., 2024; Ge et al., 2025) adopt the same approach.

To further clarify, we also calculated the average mining footprint percentage across biomes and identified those with above-average values (Supplementary Figure 4).

Comment(3) We recommend including visual methods to help explain and visualise the overlaps of data the authors use these overlaps in combination.

Response**:** We thank the reviewer for this helpful suggestion. We have created a schematic diagram (now Supplementary Figure 9) that visually summarizes our data integration and analysis workflow. We believe this addition enhances the transparency and clarity of our methodology for readers. (page 32, lines 600-604, Supplementary Figure 9)

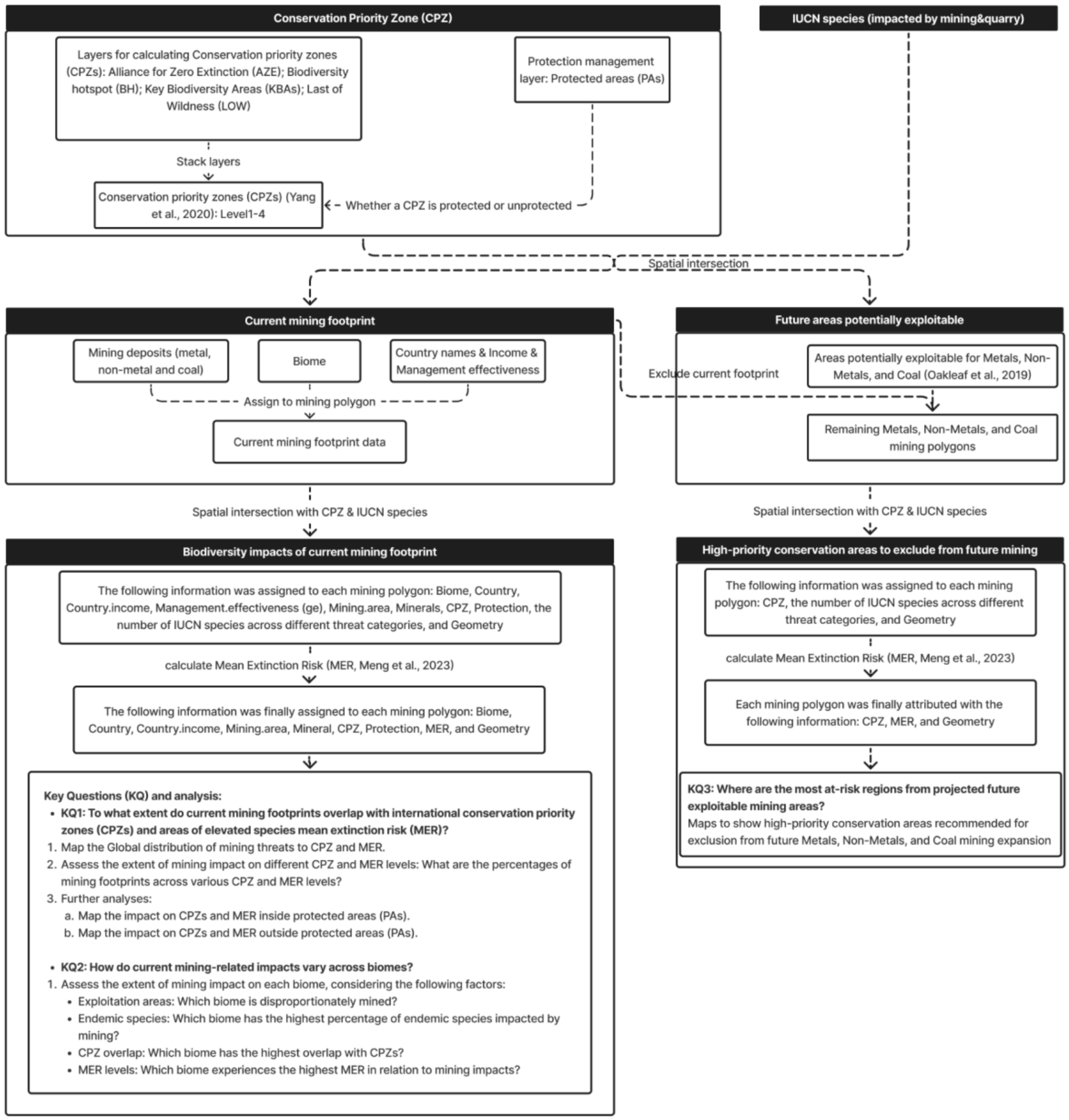

Supplementary Figure 9 Schematic diagram of the data integration and analysis workflow.

Minor comments

Abstract.

Comment(4) Avoid use of ‘However’

Response: We have reworded the sentence to avoid "However".

Now the sentence reads: "*The United Nations has committed to halting and reversing biodiversity loss by 2030. Despite this commitment, mining remains a major driver of biodiversity decline[1–4], particularly in conservation priority zones (CPZs) and areas with high species extinction risk.*" (page 2, line 13).

Comment(5) "threatening over 2,300 species, including many classified as threatened". States threatening for 2300 species, but if some of these species are not classified threatened, then we are not convinced that the authors can claim that mining is threatening those species (perhaps some benefit from mining, perhaps some are not impacted). Surely these are overlapping with?

Response: We agree that there may have been some confusion around our use of the term "threatening". To avoid misunderstanding, we have replaced "threatening" with "intersecting" in the relevant sentence, now reading: "*intersecting the ranges of more than 2,300 species, including many classified as threatened.*" (page 2, lines 18-19).

Comment(6) Please reword: "These threats span 14 biomes, with the greatest risks in fragile ecosystems such as Mangroves and Mediterranean." These biomes are disproportionately affected but greatest risk is likely within biomes with more overall mining such as Tropical moist broadleaf forest and Desert and Xeric shrubland.

Response: We have revised to "*These threats span 14 biomes, with disproportionately high relative impacts in fragile ecosystems such as Mangroves and the Mediterranean.*" (page 2, line 20).

Introduction

Comment(7) The final paragraph would benefit from clearly outlining study objectives. There is more on the methods and some conclusive points (that lack specificity) than the actual questions

Response: We thank the reviewer for this suggestion. We have revised the final paragraph of the Introduction to clearly articulate the study's primary objectives in the form of three specific research questions.

Now the last paragraph reads (objectives in yellow): "*In this study, we aim to provide a comprehensive global, spatially explicit analysis of how current and future mining activities intersect with areas of high biodiversity value. Specifically, we address three key questions: (1) To what extent do current mining footprints overlap with international conservation priority zones (CPZs) and areas of elevated species mean extinction risk (MER)? (2) How do current mining-related impacts on CPZ and MER vary across biomes? (3) Where are the most at-risk regions from projected future exploitable mining areas? By integrating high-resolution mining data with biodiversity and conservation datasets, we identify spatial priorities for mitigating biodiversity impacts and informing more sustainable mining practices. Our findings aim to support conservation planning by highlighting areas where trade-offs between mining and biodiversity protection are most pronounced, and where targeted management efforts may be needed to mitigate future ecological impacts.*" (page 5, lines 82-92).

Results

Comment(8) P7 Mangroves and the Mediterranean biome show the highest and 3rd highest percentages of extraction, respectively (Fig. 2a, Supplementary Fig. 5). "Percentage of extraction" makes it sound like they are the 1st and 3rd largest producers not that they are the highest in terms of relative proportion of extraction. Might be good to highlight any studies that focus on mining impacts from mining in mangroves, or the lack of studies if that's the case.

Response**:** We reworded the sentence to (revisions in bold): "*Mangroves and the Mediterranean biome show the highest and 3rd highest percentages of biome area affected by mining, respectively (Fig. 2a, Supplementary Fig. 4).*" (page 8, lines 158-160), to clarify that this refers to the relative proportion of each biome impacted by mining.

Regarding mining impacts in mangrove ecosystems, we have cited several relevant studies in the main text. However, to the best of our knowledge, while some local case studies and reviews exist, there is still a lack of systematic and spatially explicit assessments of biodiversity impacts across mangrove biomes. We now highlight this research gap in the revised manuscript as follows: "*The overexploitation of these biomes can lead to the irreversible loss of unique and vital ecosystems and biodiversity*[34–37]. *Nevertheless, systematic and spatially explicit studies on the biodiversity impacts of mining in mangroves and other fragile biomes, as well as comprehensive spatial planning efforts, remain limited.*" (page 8, lines 164-168).

Comment(9) The result in Figure 3b where the majority of overlap of future non-metal mineral deposits and CPZ and MER in North America is somewhat surprising, as these minerals are extracted globally in lots of highly biodiverse regions. Perhaps this is due to some bias within the Oakleaf 2019 dataset, as the maps are proxies created from density of mapped deposit? It would not be surprising that North America has more detailed maps of deposits than many regions. Either way, as the authors recommend these regions to be excluded from future development it is important to further explain this result and address this possibility.

Response**:** We appreciate the reviewer's insightful comment. In the revised manuscript (pages 11-12, lines 220-242), we now explicitly address this result and its implications for spatial planning and decision-making:

"We acknowledge that the spatial distribution of non-metal mineral deposits in our analysis may reflect underlying biases in the Oakleaf et al. (2019) dataset. Countries with more extensive geological survey coverage, such as the United States, are likely overrepresented, while regions with limited or inconsistent reporting may be underrepresented. Consequently, the apparent concentration of future non-metal mineral deposits overlapping high biodiversity risk areas in North America may be partly attributable to data availability rather than actual resource distribution.

Our prioritization maps are designed to serve as global screening tools that inform early planning rather than impose fixed development restrictions. In situations where high-risk areas cannot be avoided due to legal, economic, or geopolitical constraints, we recommend applying the mitigation hierarchy. This includes conducting site-specific biodiversity assessments, avoiding impacts where possible, and offsetting unavoidable residual impacts.

We propose a flexible approach to applying our framework. Where planning is based on the Oakleaf et al. (2019) dataset, we recommend avoiding the top 30% of areas identified as high biodiversity risk. If avoidance is not possible, decisions should be guided by thorough site-level assessments and mitigation strategies. For developments in regions not covered by the Oakleaf dataset, we suggest benchmarking new sites against the biodiversity risk values in our maps to prioritize lower-impact alternatives.

Finally, we emphasize the need for future research to expand global deposit coverage and improve the spatial resolution and consistency of mineral resource data. Addressing current data gaps will help reduce regional biases and enable more equitable, biodiversity-sensitive resource planning worldwide."

Discussion

Comment(10) Lamb et al. use IUCN threat assessments to derive hotspots of risk from mining. How do the findings in your study match to those? Might also be worth mentioning this study in the Introduction given that both use IUCN as a method of assessing risk, with this study making an advance by using mine polygons.

Response**:** Thank you for highlighting this important reference.

The relevant revised text in the Introduction now reads: "*Despite advances in understanding mining's threats to biodiversity*[2–4,17,18]*, key knowledge gaps persist. Global mining assessments typically rely on incomplete point-based data*[3,4,17]*, which fail to capture the full spatial extent of mining impacts and overlook site-specific variations in biodiversity effects*[5]*. Given the uneven global distribution of biodiversity*[19] *and the spatially variable nature of mining-related disturbance, there is a pressing need for high-resolution, spatially explicit analyses. Although some studies have assessed the local impacts of mining, they are often geographically limited and do not offer a global perspective on its threats to biodiversity*[1,20–22]*. Recent assessments have used IUCN threat listings*[18] *or modelled pressures based on deposit locations*[3]*, but these approaches are typically based on point data*[3] *or expert-derived threat data applied at coarse spatial resolutions*[18]*, limiting their ability to inform targeted conservation action for specific sites. To date, no global analysis has combined spatially explicit mining footprint data with conservation priority zones and species-level extinction risk metrics. This gap limits our ability to develop effective conservation strategies that account for real-world spatial overlap between mining and biodiversity values. In particular, our understanding of how mining overlaps with conservation priority areas and extinction risks across biomes remains limited, further hampering strategic conservation investments in critical habitats.*" (page 4, lines 51-67).

We also added this to the Discussion: "*Mining poses one of the most persistent and geographically widespread threats to biodiversity, yet global assessments have often lacked spatial precision or relied on coarse threat proxies*[3,4,17,18]*. Our study advances this field by directly integrating spatially explicit mining footprints with global biodiversity datasets, including species extinction risk and conservation priority zones, offering a more tangible and operational picture of where mining most threatens biodiversity. This approach enables a more detailed and localized assessment of mining pressure, allowing us to pinpoint critical biomes and areas at greatest risk and provides actionable insights for aligning conservation priorities with land-use decisions. Such geographic specificity and precision is particularly valuable as global conservation efforts increasingly prioritize spatially explicit strategies.*" (page 12, lines 245-254).

Figures

Comment(11) Would recommend changing the colour scheme in the Figures isn't particularly colour blind friendly. Might benefit from reducing the line width or removing country borders, so that the detail of the small pixels is easier to see.

Response**:** We have updated the colour palette in all maps to use colour-blind-friendly schemes, following the recommendations of Nature Communications (e.g., replacing red with magenta). Additionally, we minimised the thickness of country borders to enhance the visibility of fine-scale patterns in regions with dense mining activities, while retaining minimal outlines to help readers identify major geographic regions. (Fig.1, Supplementary Figure 2, 7)

Methods

Comment(12) What about species ranges that are passage or introduced etc., were these species included in the analysis? "Species range data were obtained from the IUCN Red List and BirdLife International61,66.

We focused on species threatened by mining activities, excluding species classified as Data Deficient (DD) or Extinct (EX, EW). The final dataset includes 3,094 species across five taxa: 754 amphibians, 751 plants, 697 reptiles, 463 mammals, and 429 birds. This dataset was used to assess species distributions that overlap with mining footprints and to evaluate species currently covered and not covered by protected areas."

Response**:** Thanks for raising this point. We clarify in the methods section that we have included species ranges that are passage or introduced, and why we include them within our assessment, in line with previous studies (Allan et al., 2022; Meng et al., 2023)

The revised sentences read: "*Species range data were obtained from the IUCN Red List and BirdLife International*[73,78,79]. *Following previous studies*[80,81]*, we: (1) included all documented ranges, including passage and introduced areas, as these can represent ecologically critical habitats for migration corridors, seasonal refugia, and ex-situ conservation populations*[82–84]*; 2) excluded species classified as Data Deficient (DD) or Extinct (EX, EW); and 3) identified species threatened by mining activities*." (page 37, lines 721-726).

Comment(13) "Each mining polygon was assigned a value based on CPZ levels and mean extinction risks (MER) of imperilled species. MER was calculated by averaging assigned values from IUCN Red List categories within each polygon:" Unclear, do the authors mean assigned the value of CPZ that intersects with the mining footprint?

Response**:** Yes. We clarified that mining footprints were intersected with CPZ layers, and for each intersected footprint, we assigned CPZ and MER scores based on the overlap. See revised sentence in the Methods section read: "*We assigned the value of CPZ that intersects with the mining footprint and calculated the mean extinction risks (MER) of imperilled species for this footprint*." (page 38, lines 756-758).

Comment(14) A reference for the method of assigning commodities to mines by using underlying deposit maps would be useful. There is also no comment on the potential errors that may occur from this.

Response**:** We thank the reviewer for this comment. A recently published study by Ge et al. (2025) employed a similar approach to ours by linking mining footprints to deposit types using spatial intersection. While their work validates our general method, we extend it by incorporating a more comprehensive global commodity database to assign commodity types to individual mining polygons. We have now cited Ge et al. (2025) in the revised Methods section (page 34, lines 648-649), where the text reads: "*We intersected these deposit types with the mining footprint dataset using the st_join function from the sf package*[68] *(version 1.0-16) to assign a deposit type to each mining footprint, following a similar approach to Ge et al., 2025*[37].". In addition, we have expanded the Methods section (pages 34-35, lines 665-682) to explicitly acknowledge the limitations and uncertainties of this approach. The revised text now reads:

"*While this method provides a global perspective on the potential biodiversity impacts of different mining commodities, several uncertainties remain. These include spatial mismatches between deposit maps and mining footprints, since the precise shapes and extents of many deposits are not available, as well as incomplete deposit and commodity records*[5]. *To account for these limitations, we introduced an 'unknown' category for mining footprints where a commodity type could not be reliably assigned, representing approximately 43% of the total mining footprint area (see Supplementary Table 1). Future improvements in deposit data resolution and completeness will enable more accurate identification of commodity-specific impacts*[5].

In addition, multiple commodities are often extracted from the same deposit, particularly for non-metals and coal. By aggregating commodities into three broad categories, metals, non-metals, and coal, we minimize classification uncertainty for this assessment. However, in our subsequent biodiversity impact assessment by mineral type, we acknowledge that assigning equal impacts across commodity categories within multi-commodity mining sites introduces potential bias, as different minerals may exert varying ecological pressure. Future improvements in global reporting standards and company-level production disclosures will enable more refined attribution of biodiversity impacts, allowing for a more accurate assessment of risk across commodities.".

Reviewer #2 (Remarks to the Author):

I co-reviewed this manuscript with one of the reviewers who provided the listed reports. This is part of the Nature Communications initiative to facilitate training in peer review and to provide appropriate recognition for Early Career Researchers who co-review manuscripts.

Response**:** Thank you for co-reviewing our manuscript. We appreciate the time and effort invested in the review process. We have addressed all comments raised in the report point by point and revised the manuscript accordingly.

Reviewer #3 (Remarks to the Author):

Review NCOMMS-25-18071-T

Summary and General comments:

This paper examines current and future mining threats to biodiversity "hotspots" on a global scale using a spatially explicit approach. The authors achieve this by integrating various mining footprint datasets with mineral deposit data and conducting overlay analyses alongside biodiversity datasets.

The resulting biome-specific assessments of potential future conflicts between mining and biodiversity are highly relevant to the conservation and sustainable mining communities, as well as to global environmental protection stakeholders and activists. To the best of my knowledge, this is the first study to comprehensively compile a spatially explicit, global-scale dataset linking mining threats and biodiversity hotspots. The authors have utilized the most currently available datasets, and I did not identify any significant omissions in terms of mining footprints, mineral deposits, or biodiversity hotspots.

There is one other recent similar study that is probably coarser and not as comprehensive as this one but should be included in this paper.

Reference: Lamb, Ieuan P. et al. Global threats of extractive industries to vertebrate biodiversity. Current Biology, Volume 34, Issue 16, 3673 - 3684.e4. DOI: 10.1016/j.cub.2024.06.077.

Comment1: Thus, I recommend highlighting more the novelty and added value of this particular study compared to other recent studies (e.g. by Lamb et al. (2024), Sonter et al.2018, 2020, 2022) having focused on the same topic.

Response**:** We thank the reviewer for highlighting this important reference. We have now cited Lamb et al. (2024) in the Introduction (page 4, lines 51-52, lines 58-67) and Discussions (page 12, lines 245-254). We have also highlighted the novelty and added values of our study in the Introduction and Discussion.

The relevant revised text in the Introduction now reads (modifications highlighted in yellow): "*Despite advances in understanding mining's threats to biodiversity*[2–4,17,18]*, key knowledge gaps persist. Global mining assessments typically rely on incomplete point-based data*[3,4,17]*, which fail to capture the full spatial extent of mining impacts and overlook site-specific variations in biodiversity effects*[5]*. Given the uneven global distribution of biodiversity*[19] *and the spatially variable nature of mining-related disturbance, there is a pressing need for high-resolution, spatially explicit analyses. Although some studies have assessed the local impacts of mining, they are often geographically limited and do not offer a global perspective on its threats to biodiversity*[1,20–22]*. Recent assessments have used IUCN threat listings*[18] *or modelled pressures based on deposit locations*[3]*, but these approaches are typically based on point data*[3] *or expert-derived threat data applied at coarse spatial resolutions*[18]*, limiting their ability to inform targeted conservation action for specific sites. To date, no global analysis has combined spatially explicit mining footprint data with conservation priority zones and species-level extinction risk metrics. This gap limits our ability to develop effective conservation strategies that account for real-world spatial overlap between mining and biodiversity values. In particular, our understanding of how mining overlaps with conservation priority*

areas and extinction risks across biomes remains limited, further hampering strategic conservation investments in critical habitats." (page 4, lines 51-67).

We also added this to the Discussion: "*Mining poses one of the most persistent and geographically widespread threats to biodiversity, yet global assessments have often lacked spatial precision or relied on coarse threat proxies*[3,4,17,18]*. Our study advances this field by directly integrating spatially explicit mining footprints with global biodiversity datasets, including species extinction risk and conservation priority zones, offering a more tangible and operational picture of where mining most threatens biodiversity. This approach enables a more detailed and localized assessment of mining pressure, allowing us to pinpoint critical biomes and areas at greatest risk and provides actionable insights for aligning conservation priorities with land-use decisions. Such geographic specificity and precision is particularly valuable as global conservation efforts increasingly prioritize spatially explicit strategies.*" (page 12, lines 245-254).

Comment2: The weakest part of this paper is the final section on "challenges and solutions" for achieving biodiversity-inclusive mining. While the authors' call for action is justified (i.e. halt mining in biodiversity hotspots; coordinated global actions to reconcile resource extraction with biodiversity conservation), the proposed solutions remain broad and lack specificity. For example, strengthening governance frameworks and addressing illegal mining activities are mentioned to be of high priority. Advanced technologies and nature-based solutions are mentioned to be effective impact mitigation instruments. The recommendations are very general which limits the section's added value. Questions to be addressed are : What kind of actions have been effective in the recent past? Provide concrete examples supported by references. By whom do they need to be implemented and accepted in order to be successful? For example, in the recent past local communities often were left out in so called "biodiversity offsetting" projects implemented by mining companies, which may lead to new conflicts of interest between conservation and local communities, besides the original one between mining and conservation. To strengthen this part of the paper, the authors should include concrete, evidence-based examples, where mining, biodiversity conservation, and both national and local interests successfully align to enhance the substance and credibility of this section.

Particularly, the role of local communities in integrated, sustainable land and biodiversity management is notably absent and should be addressed to provide a more comprehensive and inclusive perspective.

Response**:** We appreciate the reviewer's suggestion to strengthen this section. In response, we have significantly revised the "Challenges and Solutions" section, particularly the part on proposed solutions (now retitled "*Solutions for biodiversity-inclusive mining*" in the revised manuscript, pages 13-16) to address this concern.

Comment3: Methodologically I did not notice any flaws. Some method. details are missing here and there (see detailed comments) and I miss a sensitivity analysis (or other justification, e.g. strong references) to support the two buffers distances that were chosen. No flaws were found in the interpretation of the results. The paper is well written and structured.

Response**:** Thank you for the assessment of our methodology. We have addressed the specific methodological details noted in your detailed comments (please see our point-by-point responses below).

In response to the suggestion regarding the buffer distances, we have now included a sensitivity analysis using buffer distances ranging from 0 to 30 km in 5 km increments to assess how overlap with CPZs and mean extinction risk (MER) changes across spatial scales, as also suggested in **Comment11** and

Comment30 below. These results are provided in Supplementary Table 4 and discussed briefly in the Methods section:

"*Rather than attempting to predict indirect effects across diverse contexts, we conducted a sensitivity analysis to examine how biodiversity threats escalate as mining impacts extend beyond the footprint into surrounding areas. Specifically, we evaluate scenarios where mining threats spill over into surrounding habitats at distances of 5, 10, 15, 20, 25, 30 km, under the assumption that habitats within these buffers are entirely degraded and ecologically non-functional, which is a conservative worst-case scenario that establishes an upper bound on potential impacts. The selected distances encompass the range of off-site effects reported in previous studies. For example, Sonter et al. (2020)*[3] *applied a 10 km buffer as a conservative estimate of mining's impacts, while de Castro Pena et al. (2017)*[85] *identified indirect effects typically extending 5-10 km from mining sites.*" (pages 39, lines 771-780).

Detailed comments:

Comment4: Note: **C**ontinuous line numbering was applied to the main manuscript to point out specific lines. Further, citations of manuscript text in this review is formatted in Times New Roman font, suggestions made by the reviewer are formatted in Aptos font.

Response**:** We thank the reviewer for this helpful clarification. In the revised manuscript, we have ensured that continuous line numbers are included to facilitate easy cross-referencing of comments and revisions. We also appreciate the reviewer's clear formatting of suggestions, which helped us address each point effectively.

Comment5: Line 45:"…China, Brazil and Indonesia are mentioned as particularly affected." I miss a "most affected country (or countries) on the African continent, which also envisage economic growth but have weak social and environmental governance structures.

Response**:** We have included Congo, which is affected due to increasing mining pressures in contexts of economic growth and variable governance capacity.

Now the revised sentence reads: "*The threats are particularly severe in the biodiversity hotspots of countries like China, Brazil, Congo, and Indonesia*" (page 3, line 46).

Comment6: Line 57: "To date, no spatially explicit, global-scale analysis has been conducted, hindering the development of effective conservation strategies at both regional and global scales." Please consider mentioning Sonter et al. (2022) and Lamb et al. (2024) who performed similar studies (although maybe less comprehensive) and concretize the added value and novelty of your study. (see my general comment above).

Response**:** As we replied to the general comment above (**Comment1**), we have now cited Sonter et al., 2022 and Lamb et al. 2024 in the Introduction and Discussions. We have also highlighted the novelty and added values of our study in the Introduction and Discussion.

Comment7: Line 62: "Future mining is heavily influenced by socio-economic, geopolitical and technological trends, such as the global push for renewable energy and infrastructure development, which drive demand for specific materials like energy resources, metals, and non-metals. To address the diverse impacts of these materials on biodiversity, material-specific analyses are needed. This will enable…".

How? It is unclear how "specific analyses" will help developing effective strategies. Please be more specific.

Response**:** We have revised the text to clarify this: "*Since different commodities follow distinct future demand trajectories and spatial extraction patterns, material-specific and spatially disaggregated analyses are essential. Quantifying the biodiversity impacts of different commodities can help identify where the greatest benefits may be achieved through demand-reduction policies and support more sustainable development. Furthermore, understanding where different minerals occur and how their extraction intersects with biodiversity can guide more targeted, risk-informed strategies. For instance, minimising biodiversity loss from coal versus emerging metal mining may require prioritising conservation action in different regions. Such differentiation is key to planning sustainable mining futures that avoid or reduce biodiversity impacts.*" (pages 4-5, lines 72-81)

Comment8: Line 78-81: "Ultimately, our findings can guide conservation investments by identifying and evaluating where and how to manage trade-offs between mining activities and biodiversity conservation, while mitigating future pressures." I do agree that this study provides new results on the "where" mining threats to biodiversity are highest but not on the "how". The current version of this paper does not provide new promising recommendations on how conservation of biodiversity could become more effective. In my view this is one of the main weaknesses of this paper.

Response**:** We thank the reviewer for this important observation. In response, we revised the sentence in question to focus on the "where" aspect: "Our findings aim to support conservation planning by highlighting areas **where** trade-offs between mining and biodiversity protection are most pronounced, and **where** targeted management efforts may be needed to mitigate future ecological impacts." (page 5, lines 90-92).

Additionally, we have substantially expanded the "Challenges and Solutions" section to provide more concrete and actionable strategies for how to address mining-biodiversity trade-offs, in line with the reviewer's general comment (**Comment2**).

Comment9: Line 90: "…Southern Hemisphere. One-third of these mining areas…" It is unclear whether "one-third" refers to the previous sentence (i.e. Southern Hemisphere) or not. Please clarify in the text.

Response**:** We have revised the sentence for clarity to specify that the one-third refers to the global total mining footprint, not just the Southern Hemisphere. Now it reads: "there are several notable peaks of mining in certain regions of the Southern Hemisphere. **Globally**, approximately one-third of **all mining areas**, spanning ~50,000 km^2, an area similar to the size of Costa Rica, overlap with international CPZs" (page 6, lines 100-103).

Comment10: Line 99: "…beyond their immediate footprint…such as illegal hunting and deforestation." This is indeed a very important point. Off-site as well as indirect negative effects on biodiversity, such as air, water and soil contamination/pollution should be emphasized more as they are often neglected as well as underestimated. The authors mention other effects later on line 242, page 11, which I think is important and required. Maybe this could be highlighted a bit more.

Response**:** We revised the relevant paragraph in the Introduction to explicitly mention these broader ecological effects, and it reads: "*Notably, mining activities can also trigger off-site and indirect ecological impacts beyond their immediate footprint, including not only deforestation and illegal hunting, but also air, water, and soil contamination*[1,23–25]*. These effects can extend across landscapes and compromise biodiversity well beyond mined areas, particularly due to regulatory failures*[1]" (page 6, lines 110-114).

Comment11: Line 101ff: To account for offsite impacts ("impact spread") the authors use buffers of 1km and 5km. Please provide statistical evidence that supports your choice of buffer distances (e.g. perform a sensitivity analysis with varying buffer distances and provide these results in the supplementary material), or, in addition to this, provide references that support the choice of these buffer distances. Other studies focussing on off-site impacts have found effects on vegetation (Liu et al. 2022; Ecological Indicators) and water quality/river sediments (Lin et al. (2022; Environmental Research) and deforestation (Giljum et al. 2022) farther away than 5km from mining sites. Note: these studies are just a non-representative, random selection.

Response**:** We appreciate the reviewer's suggestion to justify the buffer distances used in our analysis. For a detailed response to this point, please refer to our reply to **Comment3**.

Comment12: Line 111ff: "Most mining occurs outside protected areas (Supplementary Fig. 2a,b), with a small percentage taking place within CPZs (Supplementary Fig. 3a). However, within protected areas, over 60% of mining footprints threaten CPZs, including 11% in zero-extinction zones (CPZ level 4) (Supplementary Fig. 3a)." Do I understand correctly, that within PAs 60% of mining footprints overlaps with CPZ and 11% of those 60% are within CPZ level 4? Not easy to grasp and understand this sentence. Also, I am wondering whether it would make sense to provide absolute numbers in brackets? Absolute numbers are provided in the Abstract and in the "future threats" section but not the "global distribution" section. Please check and adjust accordingly to be consistent throughout the entire manuscript.

Response**:** We thank the reviewer for the close reading. We confirm that both percentages refer to the total mining footprint within protected areas. We have revised the sentence in the main text to clarify this: "*However, within protected areas, over 60% (~7,000 km^2) of mining footprints threaten CPZs, and 11% (~1,000 km^2) directly intersect zero-extinction zones (CPZ level 4) (Supplementary Fig. 3a).*" (pages 7, lines 131-133).

In addition, we have added the corresponding absolute areas to ensure transparency and consistency throughout the manuscript. See also **Comment 13**.

Comment13: Line 133 and Line 146: 0.96% and 9.5% and other percentages provided here. Please consider providing the absolute area number as well.

Response**:** We have now included absolute area values alongside the percentages throughout the relevant sections of the Results text, including the statements in lines 133 (now, page 8, lines 154-157) and 146 (now pages 8-9, lines 170-173).

Comment14: Line 151: (>=50%) Here, as it is written now, it is a bit unclear what the 50% are referring to. Please consider rewriting this sentence to improve clarity.

Response**:** We have revised the sentence to clarify that this percentage reflects the proportion of each biome's mining footprint that overlaps with conservation priority zones (CPZs). The revised sentence now reads: "*Across several biomes, more than 50% of the total mining footprint overlaps with CPZs.*" (page 9, line 176).

Comment15: Line 185: "primarily located in Russia, Indonesia, China, Australia, Southern Africa, and parts of the Americas". Can you name most affected countries also for "the Americas", which is a very

large area compared to the others that you mention to keep a similar level of spatial detail in the description of the most affected countries/regions.
Response**:** We appreciate the reviewer's suggestion to improve the consistency and geographic specificity of our descriptions. We have revised the sentence to include specific countries in the Americas: "*For coal, the overlap with biodiversity hotspots is primarily located in Russia, Indonesia, China, Australia, Southern Africa, and parts of the Americas, including Brazil, Peru, Colombia, Ecuador, Chile and the United States (Fig. 3c).*" (page 10, lines 210-211).

Comment16: Line 191: "Without proactive planning and management, mining expansion could jeopardize conservation initiatives and undermine global biodiversity objectives." The results of this study justify a stronger wording here. I would argue that mining expansion not only undermines global biodiversity objectives but could lead to increased biodiversity loss.
Response**:** We agree with the reviewer that a stronger phrasing is justified to better reflect the magnitude of potential biodiversity impacts. We have revised the sentence to better capture the urgency and severity of the threat, as follows: "*Without proactive planning and management, mining expansion could drive significant biodiversity loss, compromising conservation efforts and threatening progress toward global biodiversity goals.*" (pages 10, lines 217-219).

Comment17: Line 225 - 239: This entire paragraph as part of the section on "Achieving biodiversity-inclusive mining, challenges and solutions" remains rather general. It requires improvement to be of added value. Please see my previous comment and also under "General comments". Furthermore, I elaborated some my points here once again. I hope it helps the authors and apologize for any repetition.
The authors call for a halt of mining activities in biodiversity hotspot which sounds great on paper but they do not provide pathways of how this could finally realized. The recommendations they make remain general and are well known (e.g. "strengthening governance frameworks, …addressing illegal mining activities in conservation-priority areas…"). The question is: how can frameworks become more effective?
Another example: "By embedding biodiversity safeguards throughout the lifecycle of mining projects, it is possible to align resource extraction with conservation priorities, fostering a sustainable future that supports both nature and human well-being." Can you be more concrete and provide references of success stories/examples (from the global North and South) that show that resource extraction can go together with effective conservation measures. More concretely I miss important aspects: engaging of local stakeholders, true involvement of local communities towards more inclusive, effective and meaningful biodiversity offsetting programs. Regulations require financial and human resources to be implemented and enforced. Thus, mining companies should have to engage more in this.
Response**:** We thank the reviewer again for these insightful suggestions and further explanations. Please find our replies to **Comment2**.

Comment18: Line 251: "..urgent need for a coordinated global framework to systematically monitor all mining and quarrying activities." Well, what exactly do you mean with that? I don't think this will address biodiversity conservation effectively. There exist already several global instruments, i.e forums and standards with the goal to ensure sustainable mining (IGF, EITI, IRMA Standard, etc.). I don't think another one is required.
Response**:** We thank the reviewer for raising this important clarification. Our intention was not to suggest that a completely new global framework is needed, but rather to emphasize the need to integrate biodiversity

considerations more systematically into existing frameworks. In response to this comment, we have revised the sentence to 1) Clarify that we refer to integration, not duplication. 2) Acknowledge existing initiatives, such as IGF, EITI, and IRMA. 3) Emphasize the current gap in biodiversity-specific monitoring and transparency. The revised sentences read: "*These gaps highlight the urgent need to strengthen and harmonize biodiversity-specific monitoring within existing global mining transparency initiatives, such as the Intergovernmental Forum on Mining (IGF), the Extractive Industries Transparency Initiative (EITI), and the Initiative for Responsible Mining Assurance (IRMA). While these platforms address environmental and social safeguards, biodiversity considerations are often insufficiently integrated. Systematic, spatially explicit monitoring of mining's impacts on biodiversity, particularly in terms of species extinction risk and critical habitat loss, would greatly enhance transparency, strengthen accountability, and support conservation-aligned decision-making.*" (page 17, lines 376-384)

Comment19: Line 253ff:The last paragraph sounds similarly superficial and is not convincing.
Response: The last paragraph was intended as a brief conclusion. We have revised the paragraph. (pages 17-18, lines 385-392)
It now reads: "*By grounding our analysis in actual spatial extents of mining, we provide a more tangible and operationally relevant picture of mining's biodiversity impacts, one that better supports targeted conservation action and spatial planning. This level of geographic precision is particularly valuable as global conservation efforts increasingly prioritize spatially explicit strategies, such as area-based targets under the Kunming-Montreal Global Biodiversity Framework. Our findings contribute to the growing body of evidence underscoring the need for biodiversity-inclusive land-use planning and the importance of aligning mining activities with conservation priorities at both national and global scales.*"

Figures:
Comment20: Figure 2 caption: Please **format d, e** (**bold**).
Response: Thanks, we have bolded it in the Figure 2 caption (Fig. 2).

Comment21: Figure 3: I suggest adding a figure d) showing the same map for critical minerals in addition to the a) metal, b) non-metal, and c) coal maps. While you mention this category in Supplementary Figure 9 and in the text you do not provide a map for this category. I believe this would be of high interest to the readers of this paper. (see also my comment in the methods part on a potential consistency issue.)
Response: We thank the reviewer for this thoughtful suggestion. We agree that including critical minerals is highly relevant given their strategic importance for renewable energy and technological transitions. However, we would like to clarify that the spatial data used to generate Figure 3 (**future** mining threats) are based on mineral prospectivity maps from Oakleaf et al. (2019), which categorize deposits only into **metals, non-metals, and coal**. These data do not explicitly differentiate critical minerals as a separate class. In contrast, our Supplementary Figure 9 focuses on **current** mining footprints (we have specified this in the Supplementary Figure 9 caption "*This figure corresponds to current mining footprints (main text Fig. 1)*"). For this analysis, we were able to spatially join known deposit types (including critical minerals) with mapped mining footprints and CPZs to assess their current overlap, because existing mines and deposits report extracted ore types and deposit classifications. This allows robust assessment of current impacts.
Nonetheless, to explore the reviewer's suggestion, we attempted an additional analysis by intersecting Oakleaf et al.'s data with our compiled dataset of critical mineral deposits. Only a limited number of polygons were identified as overlapping. This reflects the current lack of globally consistent, spatially

explicit information on critical mineral occurrences within undeveloped prospective mining regions. As a result, constructing a meaningful and interpretable map equivalent to Figure 3 for critical minerals is not feasible with available data.

Methods:

Comment22: Line 441, Line 442: Here you mention in brackets that 76% of the total footprint area is covered by Maus et al.'s dataset. Similarly, Tang et al., and OSM cover 64%, respectively. However, in Supplementary Table 6 these numbers are different (12%, 6% and 16%, respectively). Please clarify, harmonize or correct, either in the table or in the methods part.

Response**:** The values in Supplementary Table 6 (now Supplementary Table 8) reflect all combinations of dataset overlap used in assembling the mining footprint. For example, the row "Maus et al. & Tang et al. & OSM" (38%) indicates areas detected by all three sources, while "Maus et al. only" (12%) indicates areas found exclusively by Maus et al. To calculate the total area contributed by a given dataset (e.g., Maus et al.), we summed all rows that include that source. Specifically:

Maus et al. appear in 4 rows: 38% + 18% + 12% + 8% = 76%

Tang et al. appear in 4 rows: 38% + 18% + 6% + 2% = 64%

OSM appears in 4 rows: 38% + 16% + 8% + 2% = 64%

We have revised the caption of Supplementary Table 8 for clarification: "*This table presents the percentage of mining footprint areas attributed to different combinations of data sources, including overlaps among Maus et al. (2022), Tang et al. (2023), and OpenStreetMap (OSM, 2024). Values represent the share of the global mining footprint that falls within each source or combination of sources. Because many polygons are captured by multiple datasets, the row values are not mutually exclusive. To calculate the total contribution of each dataset (e.g., Maus, Tang, OSM), one must sum all rows in which that dataset appears. These totals reflect the overall coverage from each source used in constructing the mining footprint dataset and correspond to the percentages cited in the main text.*" (Supplementary Table 8).

Comment23: Line 446: "By complementing satellite imagery datasets such as those from Maus et al. (2022)49 and Tang et al. (2023)50, OSM enhances…" The datasets are not satellite imagery but are footprints derived from satellite imagery. Please correct the sentence to "By complementing mining footprint datasets derived from satellite imagery".

Response**:** Thank you for pointing this out. We have revised the sentence to more accurately reflect the data sources, as suggested. Now the sentence reads: "*By complementing mining footprint datasets derived from satellite imagery, such as those from Maus et al. (2022)*[61] *and Tang et al. (2023)*[62]*, OSM enhances the spatial comprehensiveness of global mining assessments.*" (page 32, lines 617-619).

Comment24: Line 448: It seems that you just added up the two binary datasets using a boolean OR. If this is not the case, please describe the overlay functions that you used more specifically. Also, please mention the date/year of each dataset. It is important to know what mining footprint year they represent. (Illegal) mining processes are very fast land change processes. The used datasets might already be outdated by now and underestimate footprints by the time this paper is published.

Response**:** These polygon datasets were merged in QGIS using a 'dissolve' operation, which removes spatial overlaps between polygons and avoids double-counting in overlapping areas. Therefore, if a mining area was present in more than one dataset, it was counted only once in the final footprint.

We have updated the Methods section (pages 32-33, lines 629-634) to reflect this polygon-based integration and specified the reference period that the mining footprint represents to improve transparency: "*The datasets were merged in QGIS software[67], and spatial overlaps between mining polygons were resolved using the 'dissolve' function, ensuring that overlapping features across datasets were counted only once. This approach allowed us to integrate data from multiple sources without duplication. The reference years are as follows: Maus et al. and Tang et al. derive from Sentinel-2 imagery collected between 2019 and 2021, while the OSM dataset was downloaded in January 2024*." (pages 32-33, lines 621-626)

Comment25: Line 469: You write that you joined "deposit type" with your merged mining footprint dataset. For the deposits you used twelve (Supplementary Table 7) different datasets. I have two method. questions regarding the deposit assignment: a) How did assign the deposit type in case of multiple minerals and/or non-minerals present at one location, and b) how did you assign it, in cases of different datasets listing different minerals, i.e. disagreeing in the deposit minerals?

Response**:** Regarding the reviewer's two questions, in the revised manuscript, our process followed the steps below:

a) Multiple minerals at one location:

When multiple mineral types (e.g., metal and non-metal) were listed within the same deposit or overlapped spatially with a mining polygon, all relevant mineral categories were assigned to that footprint (page 34, lines 654-660). For instance, a mining polygon containing both coal and non-metal deposits was classified under both categories. Consequently, the biodiversity impact of that polygon contributes to both commodity types in the analysis. We acknowledge that this approach assumes equal biodiversity impacts across commodity types, which may introduce some uncertainty since different minerals can vary in ecological impact intensity. This limitation has been clarified in the revised Methods section (pages 34-35, lines 665-682).

b) Conflicts across datasets:

Rather than treating differing commodity information from various datasets as conflicting, we interpreted them as **c**omplementary due to differences in coverage (page 34, lines 656-660). Therefore, we retained all reported commodities for a given location and integrated them into the assignment logic above. This method minimizes potential omission of valid deposit data and allows more inclusive characterization of mining activities.

Additionally, we have now cited **Ge et al. (2025)**, which recently employed a similar method of linking deposit types to mining footprints, further validating our approach. Our study extends this method by incorporating a more comprehensive and harmonized global commodity database (page 34, line 649).

Comment26: Line 476: What is the percentage of "unknown" deposit type per mining footprint of the total number (or area) of mining footprints?

Response**:** We have now clarified this point in the Methods section. Approximately 43% of the total mining footprint area could not be assigned a known commodity type due to a lack of overlap with deposit datasets. This has been noted in the revised sentence: "*Any mining footprint that did not overlap with a specific deposit type was categorized as 'unknown', accounting for approximately 43% (62,000 km^2) of the total mining footprint (Supplementary Table 1).*" (page 34, lines 652-654).

Comment27: Line 506: "A gap analysis was conducted to identify CPZs that are covered and not covered by existing protected areas." Unclear formulation, consider rephrasing.
Response**:** We have now revised the sentence for clarity to indicate that the analysis assessed which conservation priority zones (CPZs) are currently included within protected areas and which remain unprotected.
Now the sentence reads: "*We further analysed which CPZs are currently covered by existing protected areas and which remain outside formal protection.*" (page 36, lines 708-709)

Comment28: Line 535: Besides covering socio-economic factor(s) I suggest to cover also governance factors. Thus, in addition to income, I would add one of the six WorldBank governance indicators (https://www.worldbank.org/en/publication/worldwide-governance-indicators).
Response**:** We appreciate the reviewer's suggestion. Revisions have been made to the Methods (page 38, lines 741-751) and Results sections (pages 7-8, lines 143-150) and Supplementary Table 5.
In the Methods section, it now read: "*To assess the influence of national contexts on mining-related biodiversity threats, we incorporated country-level socioeconomic and governance factors. Income classifications were obtained from the World Bank (https://www.worldbank.org), which classifies countries into different groups based on gross national income per capita. Among the six World Bank governance indicators (https://www.worldbank.org), we selected government effectiveness as a representative measure, as it reflects the quality of public services and policy implementation that can influence environmental management. In addition, a Spearman correlation analysis showed strong intercorrelations ($|r_s| \geq 0.7$) between government effectiveness and the other governance indicators, supporting its use as a proxy. Therefore, income level and government effectiveness were used to represent national socio-economic and governance factors in this study.*".
In the Results section, now it reads: "*When aggregating mining threats to CPZs and MER at the national scale, regions facing the highest pressures with the highest MER, are predominantly found in low- and middle-income countries, including China, Indonesia, Brazil, Colombia, and Mexico (Supplementary Table 5). These regions are biodiversity-rich but often lack stringent regulatory oversight [16], which exacerbate species extinction risks. However, we didn't find significant influence of management effectiveness on mining biodiversity risks (Supplementary Table 5). We neither found significant differences in overlap between mining and CPZs areas across countries with different levels of income or management effectiveness.* ".

Comment29: Line 547: Please add the abbreviation definitions for LC, NT, VU, EN and CR species.
Response**:** Thank you for this helpful suggestion. We have now included the full definitions of IUCN Red List categories for clarity.
Now the sentence reads: "*where MER represents the mean extinction risks for mining driven imperilled species, and N_{LC}, N_{NT}, N_{VU}, N_{EN} and N_{CR} represent the number of Least Concern (LC), Near Threatened (NT), Vulnerable (VU), Endangered (EN), and Critically Endangered (CR) species, respectively.*" (pages 38-39, lines 761-763).

Comment30: Line 557: see my previous comment on the justification of your choice of buffer distances. Besides strengthening it with references I suggest you perform a sensitivity analysis where you perform

your calculations for a range of distances, e.g. 0 - 30 km with increment of 5km, or similar. The results can be presented as part of Supplementary Table 4.
Response**:** In response to the reviewer's suggestion, as noted in our previous reply to **Comment 3**, we have now included a sensitivity analysis.

Comment31: Line 571: "ten equal categories based on quartiles". This categorization is useful when datasets are NOT skewed. This is the case for the global mining footprint (visible in Supplementary Figure 1) but how about the CPZs and MER?
Response**:** For MER, which exhibits a right-skewed distribution, we revised our classification approach to improve interpretability. Instead of using ten equal-width bins based on quartiles, we now apply natural breaks corresponding to ecologically meaningful intervals (0-1, 1-2, 2-3, 3-4). This revision ensures a more accurate representation of extinction risk gradients and avoids effects caused by the skewed data distribution. For CPZs, skewness is not a concern because CPZ is inherently a categorical variable with four ordered classes (1-4), each representing an ecologically meaningful level of biodiversity conservation priority. Therefore, reclassification is neither necessary nor appropriate.
This is clarified in the revised Methods section (page 40, lines 789-795) "*We created global bivariate maps to visualise the relationship between mining footprints and biodiversity hotspots, specifically focusing on CPZs and MER. To do so, we reclassified mining footprint areas into ten equal categories based on quartiles. In contrast, CPZs were maintained in their original categorical structure (levels 1-4), reflecting increasing conservation priority. Similarly, for MER, we applied natural breaks corresponding to ecologically meaningful intervals (0-1, 1-2, 2-3, 3-4), which provides an interpretable representation of extinction risk gradients.*", and we updated the relevant figures (e.g., Fig. 1, Supplementary Fig. 2) to reflect this change.

Comment32: Supplementary Figure 8 caption: There is currently the same caption text (i.e. copy/paste) for panels a, b and c. Please correct.
Response**:** We thank the reviewer for spotting this oversight. We have corrected the caption of Supplementary Figure 8 (it is now Supplementary Figure 7) to accurately describe each panel (a, b, and c). Now the Supplementary Figure 7 (panels a, b and c) caption reads: "a, CPZs versus MER in regions potentially exploitable for future metal mining. b, CPZs versus MER in regions potentially exploitable for future non-metal mining. c, CPZs versus MER in regions potentially exploitable for future coal mining.".

Comment33: Supplementary Figure 9: Here you show results for the additional category "critical minerals", which is interesting. Is it possible to include this category for some of your main results/figures as well? If so, please add (see one of my previous comments on Figure 3). Similarly, Supplementary Table 1: Is it possible to add the category "critical minerals" here as well? Critical minerals are of high interest to readers and it would increase consistency throughout your entire study results.
Response**:** Thanks for the suggestion. Please find our replies to **Comment21** regarding the map of future exploitable areas for critical minerals. Concerning the addition of the "critical minerals" category in Supplementary Table 1, we have included it. This is because the table refers to current mining footprints, and sufficient information on the exploration and reporting of critical minerals is available to include this category.

Reference:

Olson, D. M. et al. Terrestrial Ecoregions of the World: A New Map of Life on Earth. BioScience 51, 933; 10.1641/0006-3568(2001)051[0933:TEOTWA]2.0.CO;2 (2001).

Meng, Z., Dong, J., Ellis, E. C., Metternicht, G., Qin, Y., Song, X. P., ... & Xiao, X. (2023). Post-2020 biodiversity framework challenged by cropland expansion in protected areas. Nature Sustainability, 6(7), 758-768.

UNEP-WCMC and IUCN (2024). Protected Planet Report 2024. UNEP-WCMC and IUCN: Cambridge, United Kingdom; Gland, Switzerland.

TNFD (2023). The TNFD Nature-related Risk and Opportunity Management and Disclosure Framework.

Aska B, Franks DM, Stringer M, Sonter LJ. Biodiversity conservation threatened by global mining wastes. Nature Sustainability. 2024 Jan;7(1):23-30.

Ge N, Xin Y, Tian P, Yu Y, Zhang Z, Zhong H, Feng K. Mapping mangrove deforestation and blue carbon loss in global supply chains between 2000 and 2019. One Earth. 2025 Jul 7.

Bisht A, Martinez-Alier J. Coastal sand mining of heavy mineral sands: Contestations, resistance, and ecological distribution conflicts at HMS extraction frontiers across the world. Journal of Industrial Ecology. 2023 Feb;27(1):238-53.

Purnama MF, Prayitno SB, Muskananfola MR, Suryanti S. Existing Conditions of Gastropod Communities in Areas Affected by Nickel Mining Overburden in the Mangrove Ecosystem of Dawi-Dawi, Southeast Sulawesi. Biotropia. 2024 Aug 7;31(2):266-76.

Sari SP, Rosalina D. Mapping and monitoring of mangrove density changes on tin mining area. Procedia Environmental Sciences. 2016 Jan 1;33:436-42.

Allan, J. R., Possingham, H. P., Atkinson, S. C., Waldron, A., Di Marco, M., Butchart, S. H., ... & Watson, J. E. (2022). The minimum land area requiring conservation attention to safeguard biodiversity. Science, 376(6597), 1094-1101.

Studds CE, Kendall BE, Murray NJ, Wilson HB, Rogers DI, Clemens RS, Gosbell K, Hassell CJ, Jessop R, Melville DS, Milton DA. Rapid population decline in migratory shorebirds relying on Yellow Sea tidal mudflats as stopover sites. Nature communications. 2017 Apr 13;8(1):14895.

Hayes FP, Berger J. Inadvertent climate refugia. Conservation Letters. 2024 Nov;17(6):e13063.

Hong Y, Yuan Z, Liu X. Global drivers of the conservation-invasion paradox. Conservation Biology. 2024 May 6:e14290.

Response to the reviewers' comments:

General remarks:

We sincerely thank the reviewers for their constructive and insightful comments, which have substantially improved the clarity and overall quality of the manuscript. We have carefully revised the manuscript in response to all comments. All modifications are highlighted in **yellow** in the revised manuscript. In this response letter, we provide point-by-point replies to each comment (reviewer comments in **black**, our responses in **blue**), and where relevant, we include the revised text from the manuscript (shown in **blue italics**). We hope these revisions satisfactorily address all concerns raised.

Summary of revisions

In response to the reviewers' comments, we have made the following key revisions:

Reviewer 1, Comment (1): We clarified the novelty of our study relative to Lamb et al. (2024), explicitly explaining the added value of spatially explicit mining data and addressing the reviewer's question of why precise mining information enables more informative, site-level assessments. We also highlight additional novel contributions beyond Lamb et al. (2024).

Reviewer 1, Comment (2): We provided a detailed explanation for the differences between our MER map and that of Lamb et al. (2024).

Reviewer 1, Comment (3): We conducted a formal statistical analysis to rigorously test "whether the overlap of mining footprints with CPZs is significantly higher within protected areas than outside at the biome level".

Reviewer 1, Comment (4): We expanded the interpretation of socio-environmental variables in the main text.

Reviewer 1, Comment (5): We have replaced the bird species icons in Fig. 2 with endemic species icons.

Reviewer 2, Comments (1-2): We revised the Methods section to explicitly reference Supplementary Table 4 for the sensitivity analysis, improving clarity and traceability. We also more clearly highlight the data gap related to mapping future critical mineral threats, as suggested.

REVIEWER COMMENTS

Reviewer #1 (Remarks to the Author):

The authors have made several changes to the manuscript in dealing with the comments, including some new analysis and several tweaks to the text to improve clarity. However, I am left with a substantial concern about the novelty of the work – the revisions if anything have not helped here and I feel the opportunity to really identify the novelty and how this work differs with others has been missed. I also feel that the authors had the opportunity to go further in their analysis to provide real inference (as was requested in the first review). Lastly, I am not keen on having three Figures, all maps. This limits reader's ability to extract meaningful patterns and also limits the study's novelty by preventing explanation of why there are particular findings made.

Response: We sincerely thank the reviewer for suggestions to improve the clarity and analytical rigour of the manuscript. Concerns regarding novelty and statistical inference are addressed in detail in our responses to **Comments (1)** and **(3)**. Regarding the figures, we appreciate the suggestion to improve interpretability. We have added new statistical panels to Fig. 1 to complement the spatial maps, which also address **Comment (4)**. Fig. 2 already integrates both spatial and statistical information, while Fig. 3 mainly illustrates future spatial risk patterns. Therefore, these two figures remain unchanged.

Comment (1) With respect to the novelty of this study vs Lamb et al. This was a concern in the first review and was also highlighted also by Ref 3. Unfortunately, the authors response to these issues has highlighted less novelty in their study relative to Lamb et al, not more.

First, there is a degree of conceptual inconsistency in the new text in the Introduction. Lamb et al used IUCN threat listings to map hotspots or mining risk, and the authors now state "but these approaches are typically based on point data3 or expert-derived threat data applied at coarse spatial resolutions18, limiting their ability to inform targeted conservation action for specific sites". The first part is true, but I would disagree with the second part. More importantly, the authors' statement undermines the utility of their own analysis, which also applied the use of the same IUCN range maps, threat assessment, and threat score to generate "conservation priority zones and species-level extinction risk metrics". They cannot have it both ways and suggest that these are poor data for assessing mining impacts, but then promote them as being rigorous for their own analysis.

In fact, CPZ integrates four datasets, at least one of which (Key Biodiversity Areas-KBAs) is entirely derived from IUCN range mapping and threat assessments. How the other three datasets generate understanding of where species have range is also an important question (ie did they use the IUCN to do so? I suspect so in e.g. AZE). Similarly, the species-level extinction risk metrics are entirely derived from using data IUCN too. Therefore, there is much similarity with the Lamb et al. study, with the only substantial difference being that in this paper the authors have used precise mine locations against which to map their largely IUCN-derived metrics.

It would have been a lot more helpful for readers to know why mapping precise locations was more informative and then how findings from this study differed (if at all) from the core hotspots of mining risk

revealed in Lamb et al. That would go a lot further in convincing of novelty than saying that Lamb et al. used "expert-derived threat data applied at coarse spatial resolutions18" [Ref 18 is Lamb et al.] but then doing so themselves.

Response: We acknowledge the reviewer's point regarding the need to clarify the novelty of our study relative to Lamb et al. (2024). Below, we provide a structured response addressing each point.

Table 1: Summary table of differences and overlap in our study compared to Lamb et al. 2024. Red indicates aspects not addressed; green highlights novel contributions uniquely addressed in each study. Detailed explanations are provided in the responses below.

**Category**	**Lamb et al., 2024**	**Our study**
Scope (geographic)	Global	Global
Scope (temporal)	Only present	Present and Future
Scope (species)	Terrestrial and Aquatic (including marine):<br>Mammal<br>Bird<br>Amphibian<br>Reptile<br>Fish<br>~~Plant~~	Terrestrial:<br>Mammal<br>Bird<br>Amphibian<br>Reptile<br>~~Fish~~<br>Plant
Scope (traits)	Assessed the effect of ecological traits on whether a species is threatened by mineral extraction, as identified by IUCN	Not addressed
Mapping impacts	Impact on all vertebrates<br>Impacts per vertebrate group<br>Impact on habitat not addressed	Impact on all species (MER; terrestrial vertebrates + plants)<br>Impact on habitats (CPZ)<br>Impact on CPZ and MER in/out protected areas
Resolution of mining impact analysis	110km * 110km (grid cell)<br><br>*[Coarse grid cell level assessment]*	Comprehensive global mining footprint (exact polygon)<br><br>*[We restrict impact to specific mining sites to get spatially-explicit impacts. This also allows us to assess impact at the biome and country level by aggregation]*<br>*Please refer to our responses below for clarification on why a precise mining footprint is important*

Sensitivity analysis of indirect impacts	Not addressed	We measured
Socio-economic assessment	Not addressed	We measured
Commodity-specific risk assessment	Not addressed	We measured
Biome-level analyses	Not addressed	We measured

i. Clarification of "why mapping precise locations was more informative" (novelty)

We acknowledge that Lamb et al. (2024) provide valuable global insights into mining-related extinction risk hotspots. However, the key distinction is not the biodiversity data (IUCN) used, but how mining impacts are represented. Lamb et al. (2024) primarily identify potential exposure of species to mining threats based on species distributions and IUCN threat classifications at coarse spatial resolutions, without incorporating spatially explicit mining polygons in their core analysis, which they also note may overestimate risks (i.e. "Because we do not identify precise locations of extraction-induced threat, in some areas we will overestimate potential risks"). In contrast, our study quantifies realised spatial overlap between mining activities and biodiversity by explicitly mapping the geographic footprint of mining. This shift from potential to realised impact enables a fundamentally different and more actionable understanding of mining-related biodiversity risk.

Building on this distinction, our approach provides several key advances:

From potential exposure to realised impact: By integrating spatially explicit mining footprints, our analysis captures where mining actually occurs relative to biodiversity features. This directly addresses a limitation acknowledged in Lamb et al. (2024) and enables assessment of realised biodiversity exposure, better reflecting spatial heterogeneity in both biodiversity and mining impacts (Maus et al., 2020; Tang et al., 2023; Maus & Werner, 2024).

Improved spatial accuracy and reduced bias: Coarse-resolution analyses (e.g., 110 × 110 km, comparable to the size of small countries such as Lebanon or half of Rwanda) are subject to scale-dependent biases that may distort risk patterns. They can **overestimate risk** (as stated in Lamb et al., 2024) by classifying cells as high risk due to the presence of large habitats supporting many threatened species, even when mining does not occur in those habitats, or at all within the cell. Conversely, they can also **underestimate risk** when cells appear low risk due to few threatened species and small species range contributions, despite mining, potentially small-scale or illegal, occurring in these sensitive areas.

In contrast, the footprint-level approach enables more accurate quantification of biodiversity exposure to mining and precise identification of high-risk overlaps. This reduces the likelihood of misleading conclusions, such as directing attention to mining areas with no actual overlap with conservation priorities while overlooking sites with high biodiversity risk.

Alignment with emerging global frameworks: Our approach is closely aligned with global frameworks such as the Taskforce on Nature-related Financial Disclosures (TNFD) and the Science Based Targets Network (SBTN), which explicitly require spatially explicit identification of impact drivers and evaluation of their biodiversity impacts (e.g., "Locate" and "Evaluate" steps in the TNFD LEAP framework) to inform targeted mitigation and restoration strategies (LEAP-P). By enabling location-specific assessment, our study provides a foundation for operational, policy-relevant biodiversity risk management.

Enhanced attribution and actionability: High-resolution mapping enables clear attribution of impacts to specific mining footprints and, potentially, their operators, supporting corporate reporting and risk management. It also allows precise identification of high-risk areas, including small-scale mining, and supports site-level prioritisation of mitigation and targeted conservation interventions, which is essential for effective conservation action.

Some of these points were noted in the Introduction (page 4, lines 51-56) and Discussion (pages 12-13, lines 267-281). In the revised manuscript, we have further strengthened and elaborated these aspects in the Discussion (pages 12-13, lines 267-281), specifically highlighting that "*Mining poses one of the most persistent and geographically widespread threats to biodiversity, yet global assessments have often lacked spatial precision or relied on coarse threat proxies3,4,17,18. Our study advances this field by directly integrating spatially explicit mining footprints with global biodiversity datasets, including species extinction risk and conservation priority zones, offering a more tangible and operational picture of where mining most threatens biodiversity, moving beyond coarse-scale or point-based assessments. This enables a shift from assessing potential exposure to quantifying realised spatial overlap between mining activities and biodiversity. By assessing footprint-level overlaps, our approach captures fine-scale spatial heterogeneity in biodiversity impacts5, avoiding potential over-estimation from coarse-resolution analyses18. It enables precise identification of high-risk sites and areas where conservation interventions are most needed. This also allows better attribution of impacts to specific mining sites and their operators, which is essential for guiding corporate and policy decisions aligned with frameworks such as TNFD. Such geographic specificity and precision is particularly valuable as global conservation efforts increasingly prioritise spatially explicit strategies.*"

ii. Additional novelties beyond Lamb et al. (2024)

Beyond the similarities between fig1b and Lamb et al. (2024)'s analysis, our study provides several entirely new insights:

Comprehensive mining footprint: We generate one of the most complete and spatially precise global mining footprint to date, including artisanal and small-scale mining, addressing gaps highlighted by Maus & Werner, 2024.

Indirect impact assessment: Sensitivity analyses, as suggested by the third reviewer, quantify how extinction risks extend beyond direct mining footprints, addressing a gap also noted by Lamb et al. (2024).

Conservation Priority Zone (CPZ) framework: Integrates multiple datasets to capture ecosystem-level conservation priorities, complementing MER (please see response **iv** for why CPZ differs from IUCN range maps).

Commodity-specific risk assessment: Identifies minerals posing different biodiversity risks, offering actionable insights for potential supply-chain or policy considerations, particularly in the context of the renewable energy transition.

Biome-level analyses: Reveals spatial heterogeneity in ecosystem vulnerability to mining, which is critical for prioritising global conservation of vulnerable ecosystems under the post-2020 Global Biodiversity Framework.

Prospective threat assessment: We assess not only current mining but also future risks, enabling forward-looking conservation and mining expansion planning.

Together, these innovations provide a comprehensive, actionable understanding of global mining-biodiversity interactions, from site-level to global patterns, direct to indirect impacts, and current to future threats, representing a substantial methodological and conceptual advance over Lamb et al. (2024) and directly supporting evidence-based conservation planning and mitigation at precise scales, aligned with TNFD and GBF.

iii. How findings from this study differed (if at all) from the core hotspots of mining risk revealed in Lamb et al.

This question overlaps with **Comment (2)**. Please refer to our detailed response there.

iv. Why CPZ is not based solely on IUCN range maps

We partially agree with the reviewer but would like to clarify. While Key Biodiversity Areas (KBAs) use IUCN species maps, they are not solely based on them. KBAs follow 11 specific criteria grouped into five higher-level categories (KBA Standards and Appeals Committee of IUCN SSC/WCPA, 2022, please refer to the guideline for details): A. Threatened biodiversity (A1 is species-based, while A2 is ecosystem-based), B. Geographically restricted biodiversity (also includes both species-based and ecosystem-based values), C. Ecological integrity, D. Biological processes, and E. Irreplaceability. Thus, KBAs consider not only species but also ecosystem-level values. Even for species-based criteria (e.g., A1 or B1), designation may consider not only species range but also the number of mature individuals, number of localities, or distinct genetic diversity. In contrast, other global conservation metrics, such as biodiversity hotspots or Last of the Wild, are entirely independent of IUCN range maps.

It is worth noting that biodiversity encompasses both species and ecosystems, both of which are crucial for conservation, as reflected in GBF targets. Accordingly, MER and CPZ capture complementary dimensions of biodiversity: CPZs identify areas of conservation priority based on multiple criteria, including species richness, endemism, habitat integrity, irreplaceability, and threatened biodiversity, while MER reflects the mean extinction risk of species present. Notably, these dimensions are not necessarily spatially congruent, as shown in previous studies (e.g., Orme et al., 2005; Jenkins et al., 2013).

We have clarified this distinction in the revised manuscript (<u>page 42, lines 855-857</u>) and emphasise that MER complements CPZs, together offering a more complete and nuanced representation of biodiversity patterns and conservation priorities. It reads: "*Importantly, CPZ and MER capture distinct dimensions of biodiversity, ranging from ecosystem-level to species-level values, and are therefore not necessarily spatially congruent.*"

Comment (2) Absent of this comparison, I have contrasted the mean extinction risk (MER) map (Fig 1b) with those in Lamb et al, and there are a few key differences, especially in that this study only highlighted a few tiny points in the Americas as being of high MER (ie blue to pink). I have to ask why this has occurred, because it is quite a strange finding and one that does not match with Fig 1a either.

I think it could be through the use of the Meng et al. scoring approach to LC to CR. They use 0 to 4 scale whereas other authors (eg Strona et al. 2018 PNAS; Wang et al. 2020 Curr Biol) used scales that upweight very rare species relative to commoner ones by a far larger degree. Anyway, this needs exploration for me to have confidence in the findings.

Response: We note the reviewer's concern about the observed differences in MER patterns and address each point below.

i. Influence of the extinction risk weighting approach

We appreciate the reviewer's suggestion. We tested the alternative weighting scheme (LC=2, NT=4, VU=8, EN=16, CR=32) and found that it does not substantially alter the spatial distribution of extinction risk hotspots (see Fig. 2 below, compared with Fig. 1-the map used in the main text). Notably, the weighting scheme used in our study is also consistent with guidelines used by the IUCN (Bubb et al., 2009).

However, we find that changing the formulation, specifically, using a summed score ($N_{LC}×0 + N_{NT}×1 + N_{VU}×2 + N_{EN}×3 + N_{CR}×4$) rather than an averaged score ($(N_{LC}×0 + N_{NT}×1 + N_{VU}×2 + N_{EN}×3 + N_{CR}×4)/(N_{LC} + N_{NT} + N_{VU} + N_{EN} + N_{CR})$), does alter the spatial pattern, resulting in slightly more pronounced hotspots in the Americas (see Fig. 3 below, compared with Fig. 1). This difference is consistent with the distinction between our MER metric and the MET approach used by Lamb et al. (2024), as discussed below ("Differences between Fig. 1b and Lamb et al.": Metric differences).

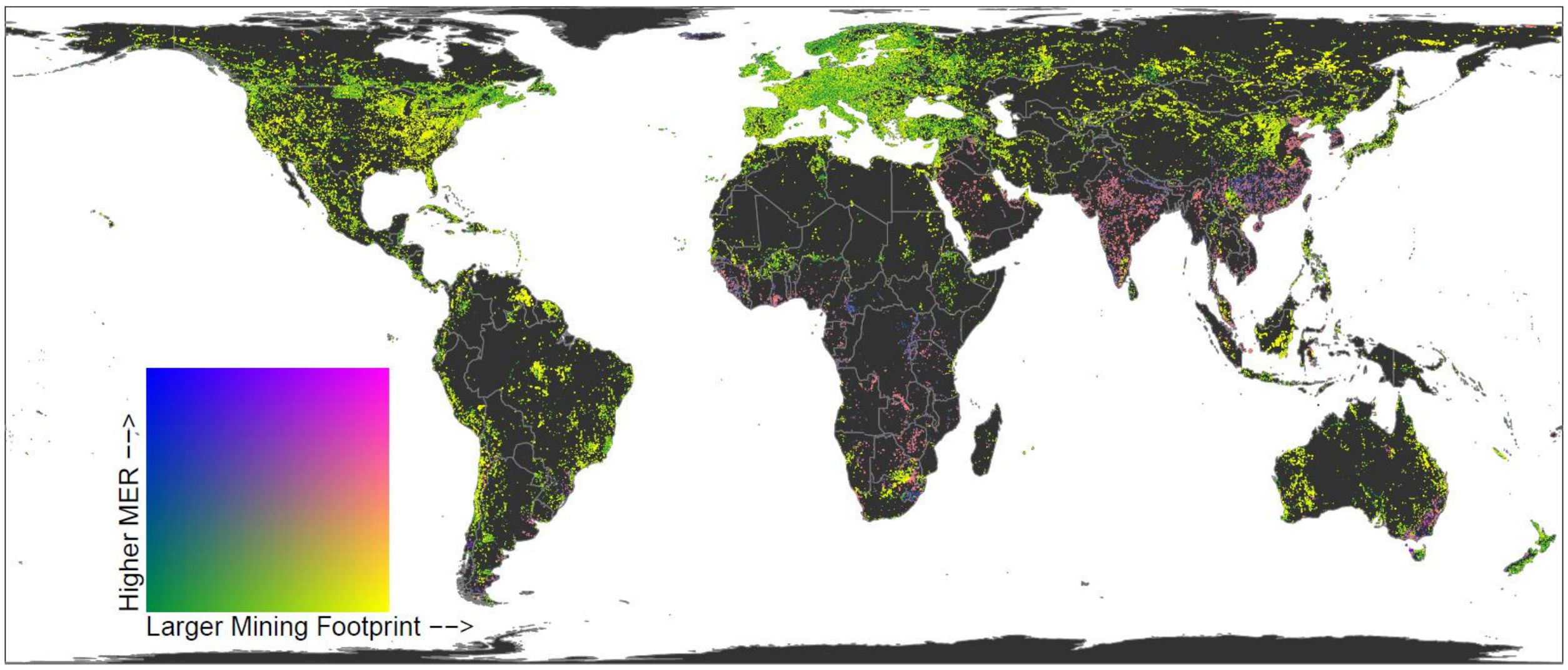

Fig.1 Map showing the relationship between MER and mining footprint polygon size (the map used in the main text).

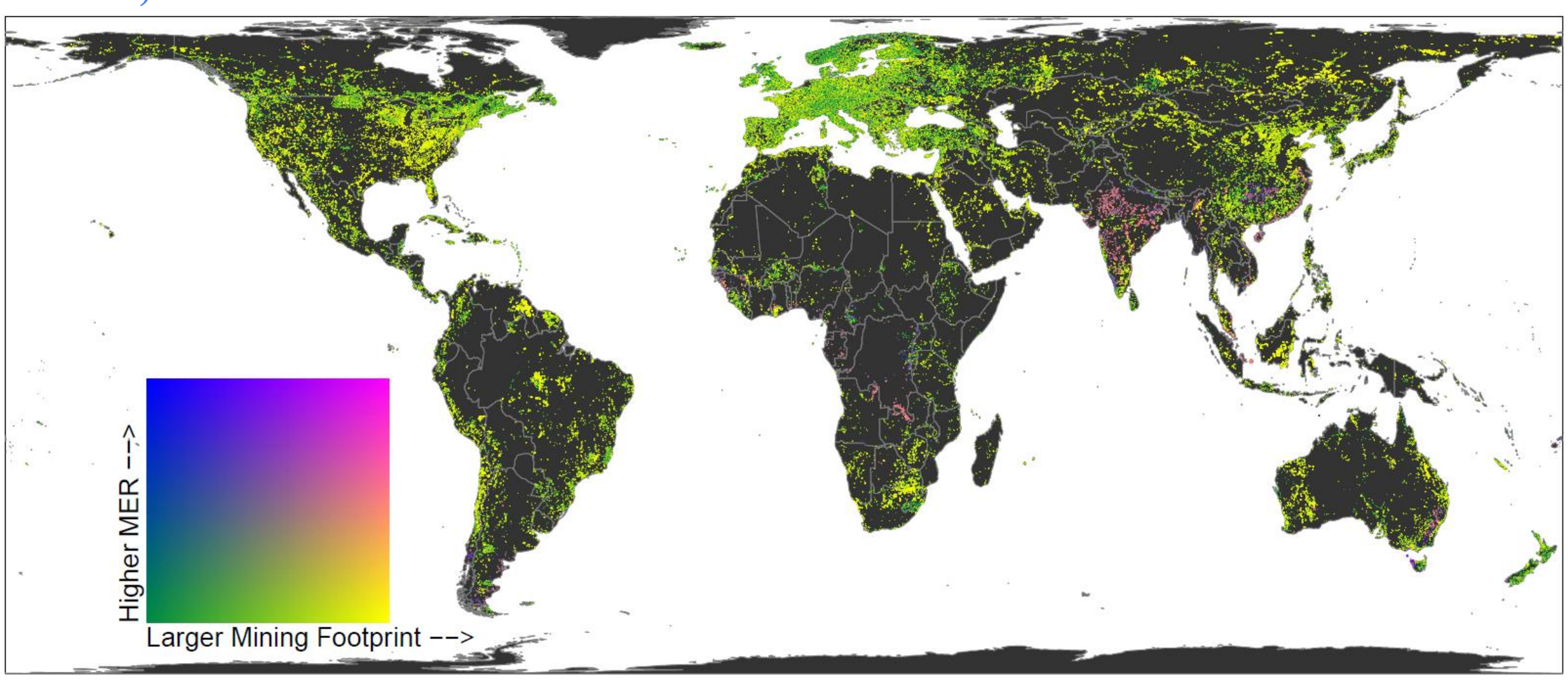

Fig.2 Map showing the relationship between MER and mining footprint polygon size, but using the alternative weighting scheme (LC=2, NT=4, VU=8, EN=16, CR=32) relative to Fig.1.

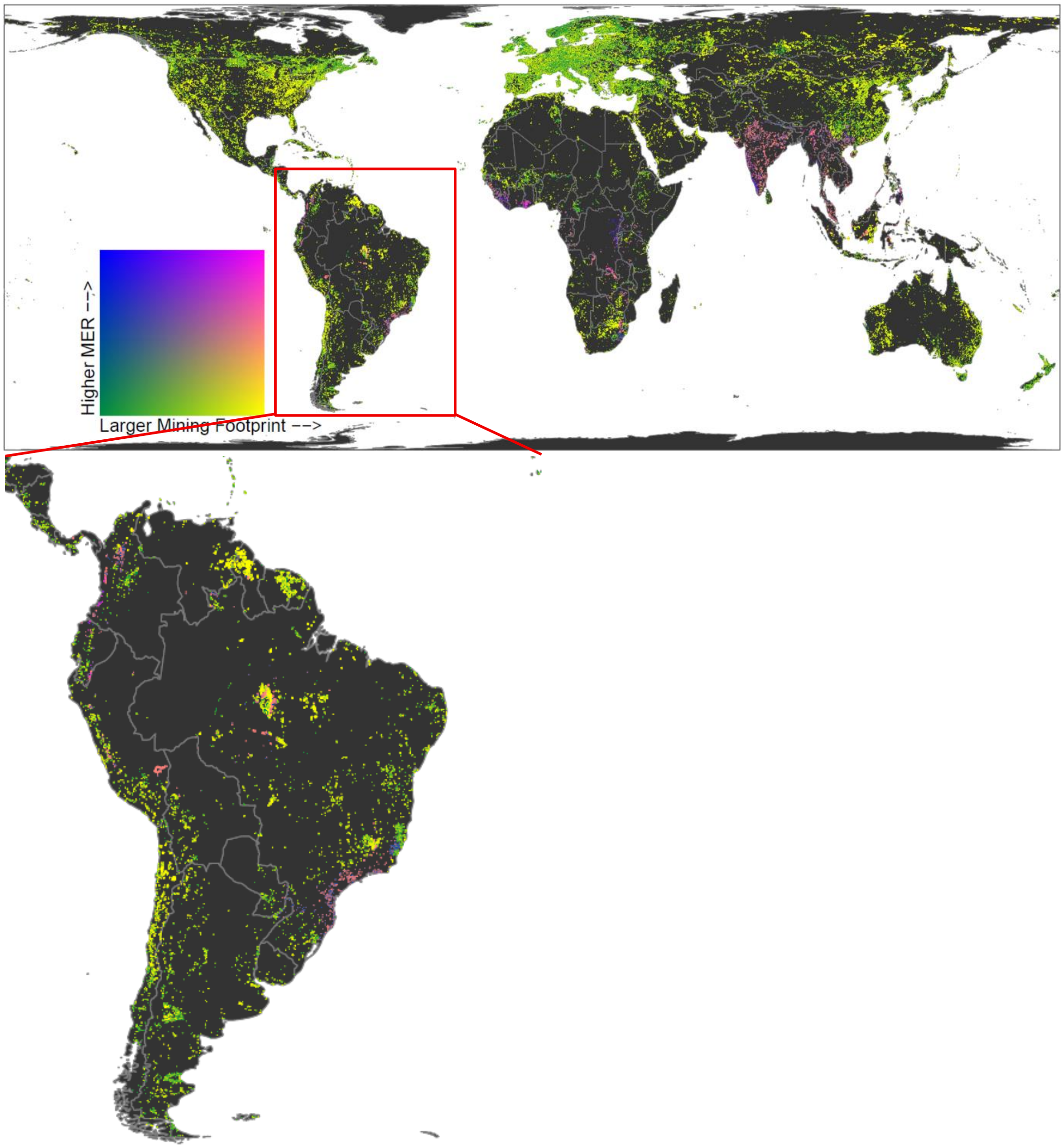

Fig.3 Map showing the relationship between MER and mining footprint polygon size, but using a summed score (N_{LC}×0 + N_{NT}×1 + N_{VU}×2 + N_{EN}×3 + N_{CR}×4) relative to Fig.1.

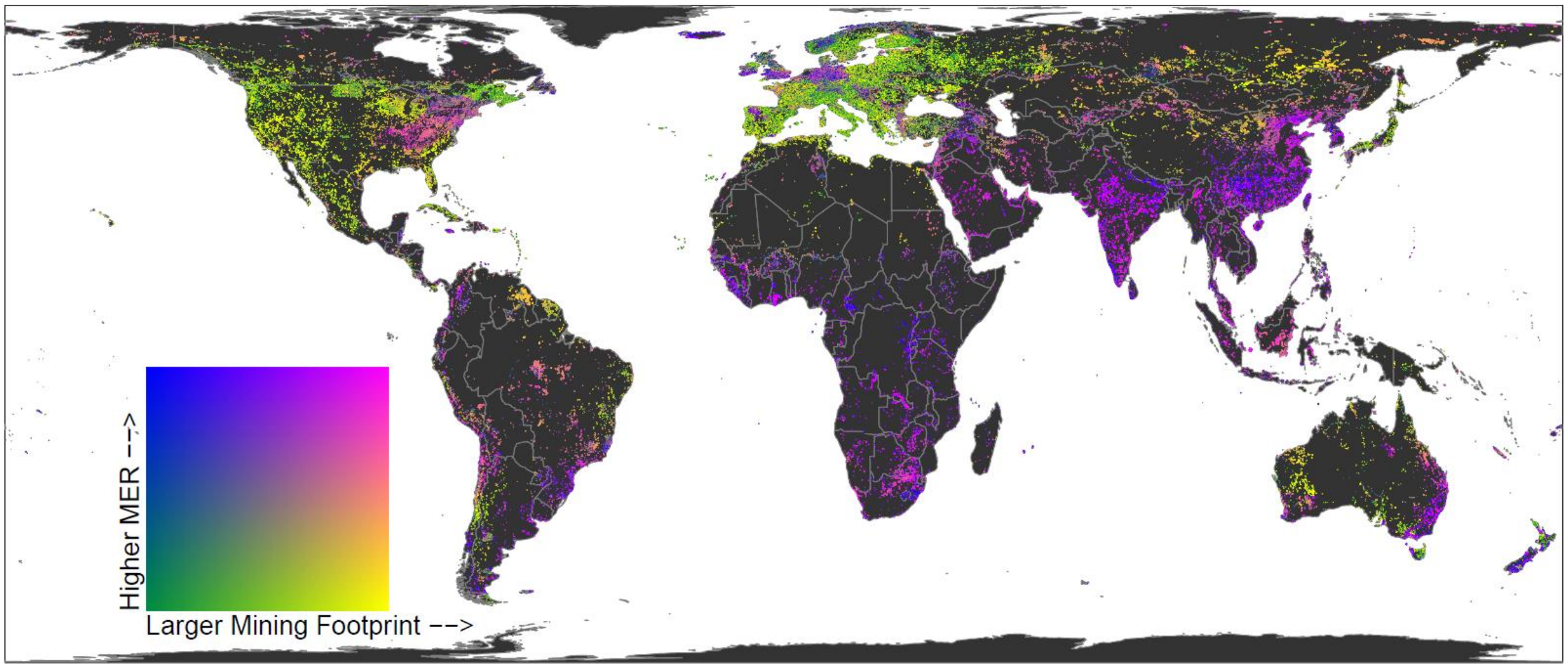

Fig.4 Map showing the relationship between MER and mining footprint polygon size, but using a quartile-based classification relative to Fig.1.

ii. Differences between Fig. 1b and Lamb et al.

The differences are primarily driven by: (1) different mapping approaches (different break points), (2) metric differences, (3) spatial resolution, and (4) data scope.

Mapping and classification methods: Classification strongly shapes spatial patterns. In our previous version, MER was visualised using a quantile-based approach (see Fig. 4 above, compared with Fig. 1), producing patterns more comparable to Lamb et al., who also applied a quartile/percentage-based method, including larger hotspots in the Americas. However, quantile-based methods can exaggerate high-risk areas in right-skewed data (MER), as noted by the third reviewer, as they assign a fixed proportion of observations to higher categories regardless of absolute values. Following reviewer suggestions, we therefore adopted a data-driven (natural breaks) classification based on absolute MER, which better reflects the underlying distribution of extinction. While this approach identifies absolute high-risk hotspots more accurately, it results in fewer areas classified as extreme and may overlook locations with lower risk values where a small proportion of threatened species still occurs.

Importantly, there are no perfect methods. To improve transparency, we have now further classified this in the figure title and it reads "*This MER classification approach highlights absolute high-risk hotspots but classifies fewer areas as extreme and may overlook lower-risk areas where a small proportion of threatened species still occurs; a quantile-based MER map is provided in the Supplementary Figure 9 for comparison and can additionally support percentage-based conservation targets*". (page 29, lines 612-616, Fig.1)

Spatial resolution and analytical scale: As we have explained in our response to **Comment (1)**, coarse resolution might overestimate the extinction risks, as also noted in Lamb et al., 2024.

Metric differences. Our MER quantifies the mean extinction risk of species threatened by mining at a location, emphasising the composition and relative share of species with different IUCN threat categories. In contrast, Lamb et al.'s MET reflects species richness potentially threatened by mining and their relative distribution across the globe, without weighting extinction risk (IUCN categories).

As a result, the two indicators capture different dimensions of biodiversity. For example, areas with high species richness potentially affected by mining, yet primarily consisting of lower-risk species relative to overall species composition, may exhibit high MET but relatively low MER.

Consequently, differences in metric design can lead to contrasting spatial patterns and interpretations of mining-related biodiversity impacts. This sensitivity to methodological choices is also evident in Lamb et al. (2024), where alternative approaches produce markedly different hotspot distributions (**see Lamb et al., 2024, Fig. 3, left vs. right panels**).

Differences in data scope: Our analysis focuses on terrestrial plants and vertebrates (birds, mammals, amphibians, and reptiles), whereas Lamb et al. focus on all vertebrates, including also freshwater and marine fishes, which they identify as having the highest number of species with METs and contributing substantially to global extinction risk patterns (Lamb et al., 2024). Excluding these groups in our study may reduce hotspot prominence in certain regions. The impacts on freshwater and marine species from water pollution or deep-sea (Lamb et al., 2024) mining are beyond our study. We have acknowledged this in the "Limitations and Conclusions" section: "*In addition, our analysis does not include freshwater and marine taxa (e.g., fishes), which have been identified as contributing substantially to global mining-related extinction risk patterns18. Assessing these aquatic systems requires distinct data and methods, such as hydrological, pollution, and offshore extraction models. As a result, risks to aquatic biodiversity may be underrepresented in our study, highlighting the need for future research specifically integrating freshwater and marine taxa.*" (page 18, lines 398-404)

Together, these differences in classification, scope, resolution, and metric design explain the contrasting spatial patterns between the two studies.

iii. Differences between Fig. 1a (CPZ) and Fig. 1b (MER)

As we explained in the response to **Comment (1)**, CPZ and MER capture distinct dimensions of biodiversity; thus, the two maps don't look the same (please see our response to **Comment 1**).

Comment (3) Re. contrasting the percentage of mines inside and outside PAs, this has not actually used any formal statistical interrogation but this was something highlighted in the first review as being important. Rather than offering statements of weak confidence i.e. "When comparing mining inside and outside protected areas within the same biome, the proportional overlap of mining footprints with CPZs is generally higher inside protected areas, although similar in Mangroves and Tropical & Subtropical Coniferous Forests", the authors should have conducted an analysis and then been explicit in saying that mines in X Y Z biomes were located significantly more inside than outside PAs.

Response**:** To more rigorously assess whether the proportional overlap of mining footprints with CPZs at the biome level is higher within protected areas than outside, we conducted a statistical analysis comparing mining overlap between protection categories across biomes.

Now the revised main text reads: "*When comparing mining inside and outside protected areas within the same biome, the proportional overlap of mining footprints with CPZs, aggregated at the biome level, is significantly higher inside protected areas than outside (Kruskal-Wallis test: $\chi^2 = 6.19$, d.f. = 1, $P = 0.01$; Supplementary Figure 6a). Polygon-level analyses further support this result, indicating that mining polygons are significantly more likely to overlap CPZs within protected areas than outside (Supplementary Table 5). This effect is consistent across most biomes (12 out of 14), where the probability of overlap is significantly higher within protected areas, while no significant differences are detected in Mangroves and Tropical & Subtropical Coniferous Forests (Supplementary Figure 6b). These results indicate that mining activities disproportionately overlap with high-priority biodiversity areas within existing protected areas,*

highlighting potential challenges for conservation effectiveness and the implementation of mitigation strategies." (pages 9-10, lines 192-203)

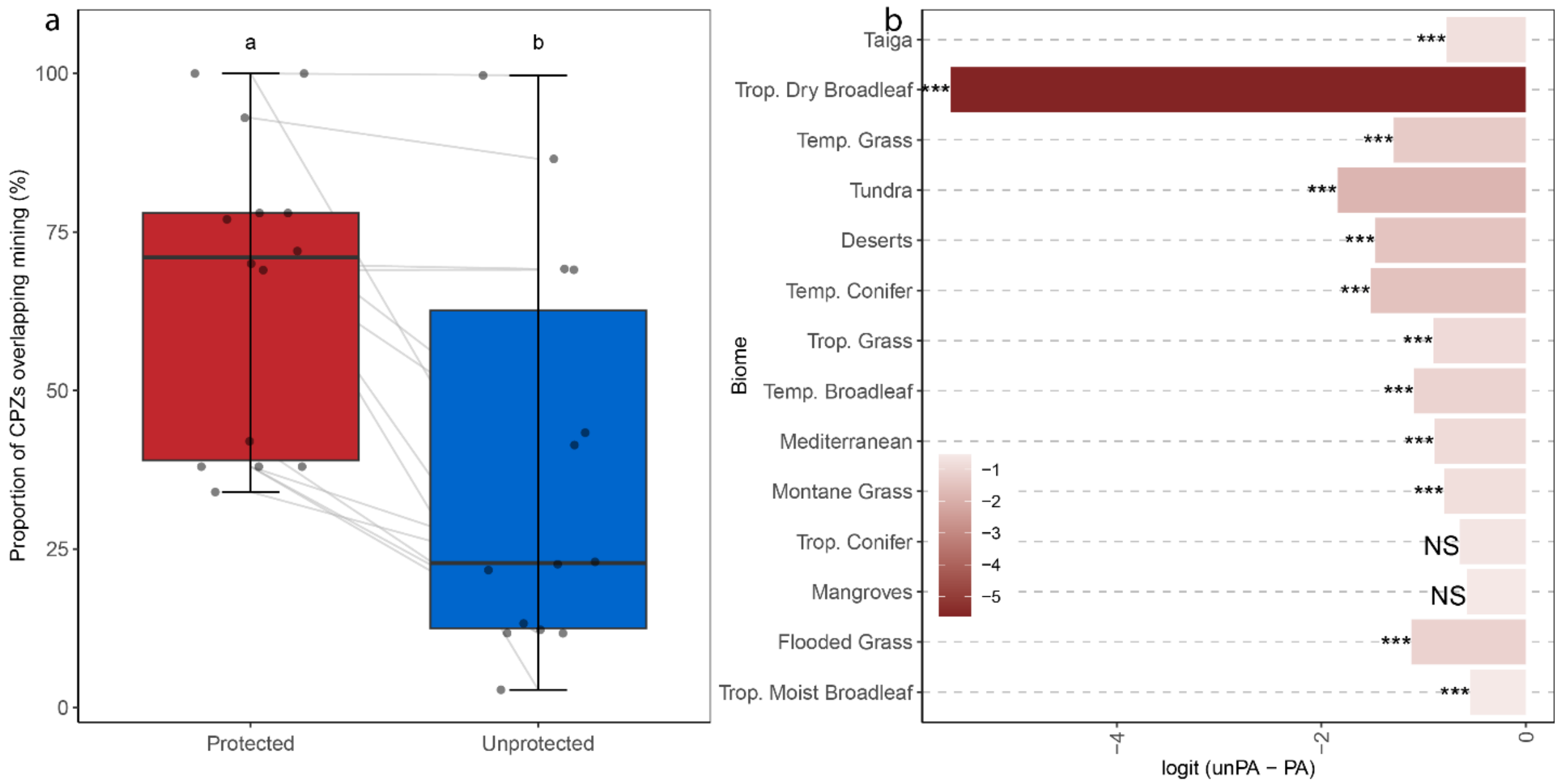

Supplementary Figure 6: Comparison of mining overlap with conservation priority zones (CPZs) inside and outside protected areas across biomes. a, *Boxplots show the distribution of the proportion of mining footprints overlapping CPZs across global biomes, separated by protection status (red: protected areas; blue: unprotected areas). Each point represents a biome. The central line indicates the median, boxes show the interquartile range, and whiskers extend to 1.5× the interquartile range. Letters above boxplots indicate significant differences between groups based on Kruskal-Wallis post-hoc tests ($\chi 2 = 6.19$, d.f. $= 1$, $P = 0.01$). Grey lines connect paired values for the same biome between protected and unprotected areas.* ***b***, *Bar plots showing differences in the probability of mining polygons overlapping CPZ between protected areas and unprotected areas across biomes. Negative values indicate a higher probability of overlap within protected Areas. Estimates were obtained from estimated marginal means of binomial GLMs (Protection × Biome + Income or ge). Statistical significance was assessed using Wald tests (*p < 0.05; **p < 0.01; **p < 0.001).*

The revised methods read: "*To evaluate whether mining overlap with CPZs at biome level differs between protected and unprotected areas, we performed a Kruskal-Wallis rank-sum test comparing the proportion of mining footprints overlapping CPZs between protection categories across biomes (Supplementary Figure 6a). As the analysis is conducted at the biome scale, with a single aggregated value per biome and protection category, formal statistical comparisons within individual biomes are not possible. To further evaluate the consistency of the observed pattern across biomes and enable statistical inference, we conducted a complementary analysis at the mining footprint (polygon) level. Specifically, we tested whether mining polygons are more likely to overlap CPZ within protected areas (PAs) than outside PAs. Each mining polygon was treated as a binary observation, coded as 1 if it intersected CPZ and 0 otherwise. We fitted a binomial generalized linear model (GLM) with a logit link, including protection status (Protection), biome (Biome), and their interaction. We also considered the potential effects of income level and government effectiveness (ge). However, these two variables are strongly correlated and capture*

overlapping dimensions of socioeconomic development (Supplementary Figure 12). Including both variables in a single model would introduce multicollinearity and complicate interpretation of their individual effects. Given prior evidence that national income influences biodiversity outcomes30, we included income level in the main model. To additionally assess whether governance exerts an independent effect, we fitted a separate supporting model including ge instead of income. This two-model approach allows for robust and interpretable inference by isolating the effects of income and governance, while avoiding instability arising from collinearity. The model formulations were as follows:

*Main model: Intersect CPZ ~ Protection * Biome + Income level*

*Supporting model: Intersect CPZ ~ Protection * Biome + ge*

To evaluate overall differences between protected and unprotected areas, we used estimated marginal means (EMMs) derived from the main model using the emmeans package91 (version 1.8.8), averaging the effect of protection status across all biomes (Supplementary Table 5). Statistical significance was determined using Wald tests. To assess biome-specific differences, we conducted pairwise comparisons of protected versus unprotected polygons within each biome using EMMs conditioned on biome identity (Supplementary Figure 6b). The marginal effect of income level was evaluated from its main effect in the main model, while the marginal effect of ge was assessed from its main effect in the supporting model (Supplementary Table 5)." (pages 43-44, lines 883-915)

Comment (4) The requested analysis from the first review identifying socio-environmental predictors reveals interesting findings, but these are not mentioned in the main text, just pointed to in a supplementary table. What readers should glean about these patterns should have been spelled out and potentially accompanied by Figures.

Response**:** We appreciate the reviewer's suggestion. This comment could lead to two points. While we suspect the reviewer was primarily referring to the second point (ii), we provide responses to both below.

i. MER patterns inside vs. outside PAs

This comment relates to the question raised in the first review: "To really answer the important question of whether mining within protected areas (PAs) drives higher MER in certain biomes compared to areas outside PAs, the authors should directly contrast within- and outside-PA effects within a single model, probably accounting for socio-environmental variables."

To address this, we conducted a formal statistical analysis directly comparing MER inside and outside PAs across biomes within one mode, while also accounting for the effects of income and governance effectiveness. This analysis provides a robust answer to whether mining within PAs is associated with higher MER in specific biomes relative to areas outside PAs. These patterns were already reported in the original manuscript. (page 10, lines 207-212)

ii. Effects of national income and governance effectiveness

We suspect the reviewer's comment regarding "patterns not mentioned in the main text" specifically refers to socio-environmental predictors of national income and government effectiveness, previously only reported in supplementary tables. We have now integrated these results into a dedicated paragraph in the section "Global distribution of mining and biodiversity threats", which discusses the influence of national income and governance on mining impacts on biodiversity (both MER and CPZ, pages 7-8, lines 142-163). This placement ensures that these important patterns are clearly presented in the main text and aligns better with the overall narrative and logical flow of the manuscript, than including them in the section "Biome-

specific threats.”. We also agree with the reviewer on the potential value of a figure. Accordingly, we have added two panels to Fig. 1 presenting the effects of income and governance on CPZ and MER separately. We believe this addition also strengthens the interpretation of meaningful patterns, complementing the spatial maps, and allows readers to directly see the influence of socio-environmental predictors on mining-related biodiversity risks.

It now reads: “*When summarising polygon-level mining threats to CPZs and MER at the national level, regions facing the highest pressures with the highest CPZs and MER, are predominantly found in lower-income countries, including China, Indonesia, Brazil, Colombia, and Mexico (Fig.1, Supplementary Table 5-6). This pattern aligns with previous studies showing that extinction risk tends to be higher in lower-income countries30,31. These regions are often biodiversity-rich, but face intersecting challenges, including a lack of stringent regulatory oversight, strong dependence on natural resource extraction, competing economic development priorities, and higher levels of economic inequality, all of which can exacerbate species extinction risks15,32–34. We also found significant influence of management effectiveness on mining biodiversity risks (Fig.1 c-d, Supplementary Table 5-6), which appears to conflict with some previous findings reporting no effect of management30. Management effectiveness is highly correlated with national income, and more effective governance can substantially reduce illegal mining, which is a major driver of biodiversity loss15,23. This pattern likely reflects that both national income and governance constrain the extent to which mining activities overlap high-priority conservation areas and regions of high extinction risk. It may also indicate that many conservation priority zones and habitats for threatened species are not fully integrated into national planning and regulatory frameworks globally35, particularly in lower-income or poorly governed countries, potentially leading to under-recognition of biodiversity risks and challenges for achieving global conservation targets such as the Global Biodiversity Framework. Together, these findings suggest that mining pressures on conservation priority areas and extinction risks are disproportionately severe in lower-income countries, highlighting the need for targeted mitigation and strengthened conservation efforts in these regions.*” (pages 7-8, lines 142-163)

Comment (5) Figure 2 – why use an image of a golden-winged warbler everywhere. First it is not an endemic and second it doesn’t live globally either. At least an endemic species could be pictured.

Response**:** We appreciate the reviewer’s suggestion. We have replaced the species image in Fig. 2 with endemic species icons.

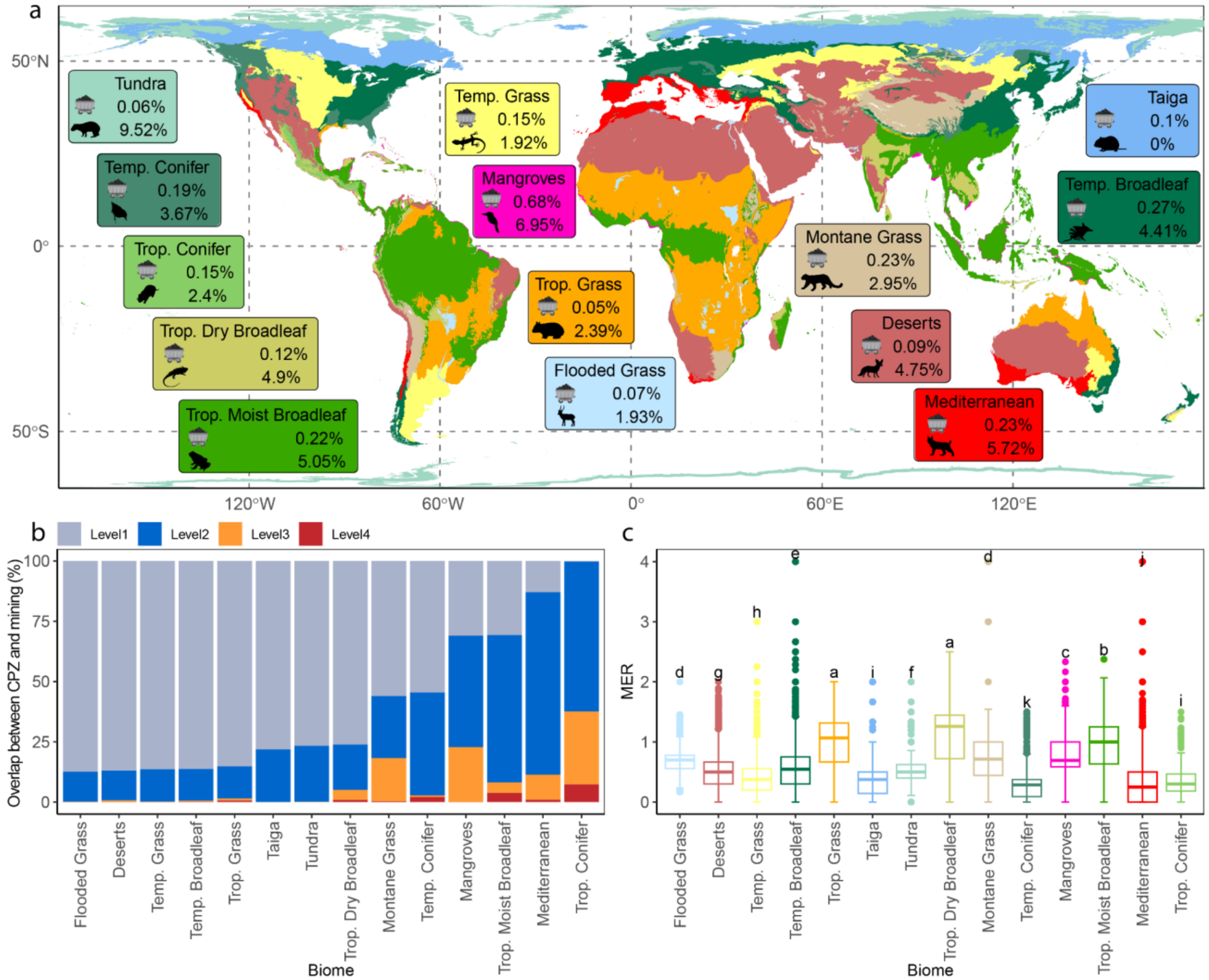
a
Tundra 0.06% 9.52%
Temp. Conifer 0.19% 3.67%
Trop. Conifer 0.15% 2.4%
Trop. Dry Broadleaf 0.12% 4.9%
Trop. Moist Broadleaf 0.22% 5.05%
Temp. Grass 0.15% 1.92%
Mangroves 0.68% 6.95%
Trop. Grass 0.05% 2.39%
Flooded Grass 0.07% 1.93%
Montane Grass 0.23% 2.95%
Deserts 0.09% 4.75%
Mediterranean 0.23% 5.72%
Taiga 0.1% 0%
Temp. Broadleaf 0.27% 4.41%
50°N
0°
50°S
120°W
60°W
0°
60°E
120°E
b
Level1 Level2 Level3 Level4
Overlap between CPZ and mining (%)
Biome
c
MER
Biome
Flooded Grass
Deserts
Temp. Grass
Temp. Broadleaf
Trop. Grass
Taiga
Tundra
Trop. Dry Broadleaf
Montane Grass
Temp. Conifer
Mangroves
Trop. Moist Broadleaf
Mediterranean
Trop. Conifer

Reviewer #2 (Remarks to the Author):

I co-reviewed this manuscript with one of the reviewers who provided the listed reports. This is part of the Nature Communications initiative to facilitate training in peer review and to provide appropriate recognition for Early Career Researchers who co-review manuscripts.
Response**:** We thank the reviewer for co-reviewing our manuscript as part of the Nature Communications peer review training initiative. We appreciate the time and effort invested in the review process. We have addressed all comments raised in the report point by point and revised the manuscript accordingly.

Reviewer #3 (Remarks to the Author):

Comment1 (**formerly Comment 30**): The authors addressed this comment by performing a sensitivity analysis and adding the results in Supplementary Table 4. The sensitivity analysis is described in the Methods section but it does not yet explicitly refer to Supplementary Table 4. I suggest adding "(Supplementary Table 4)". It will help readers easily locate the corresponding sensitivity analysis results in the supplementary materials.

Response**:** We thank the reviewer for this helpful suggestion. We have revised the Methods section to explicitly refer to the results of the sensitivity analysis (Supplementary Table 4). This addition improves clarity and helps readers easily locate the corresponding results in the Supplementary Information. (page 42, lines 850-851)

Comment2 (formerly Comment 21): I would like to thank the authors for considering my suggestion and assessing the feasibility of developing a prospectivity map for critical minerals. It is unfortunate that the necessary data are not available from Oakleaf et al. (2019).

It may be worthwhile to highlight this data gap or limitation more explicitly in the Methods section (near lines 669–682 of the revised manuscript).

I also thank the authors for adding the available information on critical minerals where possible (Supplementary Table 1), i.e. adding this information on current critical mineral mining footprints.

Response**:** We appreciate the reviewer's suggestion to explicitly acknowledge this data limitation. We have now revised the Methods section to clearly highlight this constraint and to emphasise the need for the development of globally consistent, spatially explicit data on critical minerals in undeveloped mining areas in future studies.

It reads: "*For future distribution of critical minerals, the used dataset (Oakleaf et al., 2019)46 provides information for metals, non-metals, and coal, but does not distinguish critical minerals as a separate category. Although we compiled data on current mines and deposits for 38 key critical minerals (Supplementary Table 10), generating a global prospectivity map by overlapping these deposits with potential future mining areas was not feasible. This limitation arises because many critical mineral datasets are point-based and largely based on currently exploited deposits rather than undiscovered or prospective resources. This highlights a key data gap: spatially explicit, globally consistent information on prospective critical mineral deposits is currently lacking. Addressing this gap will be important for assessing future expansion of critical mineral and its potential interactions with biodiversity conservation, particularly in the context of increasing demand for minerals required for the clean energy transition.*" (pages 46-47, lines 958-968)

Reference:

Maus V, Giljum S, Gutschlhofer J, da Silva DM, Probst M, Gass SL, Luckeneder S, Lieber M, McCallum I. A global-scale data set of mining areas. Scientific data. 2020 Sep 8;7(1):289.

Tang L, Werner TT. Global mining footprint mapped from high-resolution satellite imagery. Communications Earth & Environment. 2023 Apr 22;4(1):134.

Maus V, Werner TT. Impacts for half of the world's mining areas are undocumented. Nature. 2024 Jan 4;625(7993):26-9.

Lamb IP, Massam MR, Mills SC, Bryant RG, Edwards DP. Global threats of extractive industries to vertebrate biodiversity. Current Biology. 2024 Aug 19;34(16):3673-84.

KBA Standards and Appeals Committee of IUCN SSC/WCPA. Guidelines for using A global standard for the identification of Key Biodiversity Areas : version 1.2. 2022. Gland, Switzerland: IUCN.

Orme CD, Davies RG, Burgess M, Eigenbrod F, Pickup N, Olson VA, Webster AJ, Ding TS, Rasmussen PC, Ridgely RS, Stattersfield AJ. Global hotspots of species richness are not congruent with endemism or threat. Nature. 2005 Aug 18;436(7053):1016-9.

Jenkins CN, Pimm SL, Joppa LN. Global patterns of terrestrial vertebrate diversity and conservation. Proceedings of the National Academy of Sciences. 2013 Jul 9;110(28):E2602-10.

Bubb P, Butchart S, Collen B, Dublin H, Kapos V, Pollock C, Stuart S, Vié JC. IUCN Red List index: Guidance for national and regional use. Version 1.1. IUCN; 2009.

Response to the reviewer and editor's comments

General remarks:
We sincerely thank the reviewer and editor for their final suggestions. We have revised the manuscript in response to all remaining minor comments. In this response letter, we provide point-by-point replies to each comment (reviewer comments in **black**, our responses in **blue**). We hope these revisions satisfactorily address all concerns raised.

REVIEWER COMMENTS

Reviewer #1 (Remarks to the Author):

The authors have engaged with my request for a formal analysis to back up their patterns and have strengthened their presentation of several other findings. I have a one remaining issue, which can be simply solved by deleting some text, and a couple of minor edits.

Comment (1) The 5 km buffer analysis of "total habitat destruction" as a multiplicative of mine footprint is quite extreme but makes an important point ("Under the scenario where these impacts spread just 5 km beyond the footprint, the area of overlap with various levels of CPZs and MER could increase by 28-55 times and 53-566 times, respectively, assuming total habitat destruction with no remaining ecological functionality"). However, "If the impact radius expands to 30 km, the affected area overlapping with different levels of CPZs and MER could escalate to 240-324 times and 269-751 times the current mining footprint's threats (Supplementary Table 4)" moves towards hyperbole and I suggest deleting it – I cannot think of a single mine that has destroyed ecological function in a 30 km radius.
Response: We sincerely thank the reviewer for this suggestion. We have deleted this sentence from the main text. (lines 115-120)

Comment (2) L51 paragraph is very long, split.
Response: We have split the paragraph at L51 into three paragraphs to improve readability. (lines 50-81)

Comment (3) L218-219, join as a single paragraph.
Response**:** We have combined the two paragraphs at L218–219 into a single paragraph, as suggested. (lines 220-229)

Comment (4) Figure 1: there is no need for the panels C and D to be so deep, there is just a lot of wasted whitespace.
Response**:** We appreciate the reviewer's suggestion. We have reduced the height of panels C and D to minimise the unused whitespace and improve the overall layout of Figure 1.
